# Tunable solid-state fluorescent materials for supramolecular encryption

**DOI:** 10.1038/ncomms7884

**Published:** 2015-04-22

**Authors:** Xisen Hou, Chenfeng Ke, Carson J. Bruns, Paul R. McGonigal, Roger B. Pettman, J. Fraser Stoddart

**Affiliations:** 1 Department of Chemistry, Northwestern University, Evanston, Illinois 60208-3113, USA; 2 Cycladex, c/o Innovation and New Ventures Office, Northwestern University, 1800 Sherman Avenue, Suite 504, Evanston, Illinois 60201-3789, USA

## Abstract

Tunable solid-state fluorescent materials are ideal for applications in security printing technologies. A document possesses a high level of security if its encrypted information can be authenticated without being decoded, while also being resistant to counterfeiting. Herein, we describe a heterorotaxane with tunable solid-state fluorescent emissions enabled through reversible manipulation of its aggregation by supramolecular encapsulation. The dynamic nature of this fluorescent material is based on a complex set of equilibria, whose fluorescence output depends non-linearly on the chemical inputs and the composition of the paper. By applying this system in fluorescent security inks, the information encoded in polychromic images can be protected in such a way that it is close to impossible to reverse engineer, as well as being easy to verify. This system constitutes a unique application of responsive complex equilibria in the form of a cryptographic algorithm that protects valuable information printed using tunable solid-state fluorescent materials.

## Abstract (web summary)

Solid-state fluorescent materials show promise for potential applications in security and anti-counterfeiting technologies. Here, the authors report a heterorotaxane which has found application in security inks with highly tunable solid-state fluorescence through supramolecular encapsulation.

## Graphical abstract

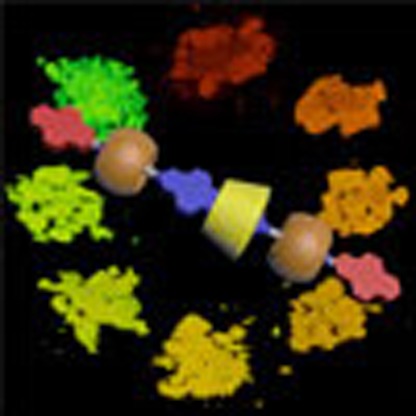

Photoluminescent solid-state materials have been widely applied in dye lasers^1^, organic light emitting diodes (OLEDs)^2^,^3^,^4^, data recording and storage^5^,^6^, and security printing^7^,^8^. These materials can be easily applied inexpensively to different surfaces and have been implemented widely as security inks to protect high-value merchandise, government documents and banknotes^9^. Materials with static luminescent outputs, however, are familiar to counterfeiters. In contrast, stimuli-responsive photoluminescent materials, which change their optical outputs in response to external stimuli, possess extra security features that are difficult to mimic, making them suitable for the next generation of security printing. In recent years, a series of stimuli-responsive photoluminescent materials, including those that are thermochromic^10^,^11^,^12^,^13^, photochromic^14^,^15^,^16^, mechanochromic^17^,^18^,^19^, solvochromic^20^,^21^,^22^,^23^ and electrochromic^24^,^25^, have been developed. These smart materials respond to external stimuli with reversible changes to their chemical constitutions or superstructures in the solid state, causing them to emit different luminescent colours. Coding these fluorescent colours in one or two dimensions –a practice which is similar to digital coding (for example, barcodes and QR codes) in computer science– has been suggested^26^ as a potential strategy to prevent tampering or counterfeiting. Current stimuli-responsive photoluminescent materials, however, can only provide a small matrix of colours as optical codes. Developing wide-spectrum tunable photoluminescent solid-state materials with multiple fluorescent emissions (a large matrix) remains a major challenge.

Herein, we report the unexpected discovery, during synthesis by a cooperative capture strategy^27^,^28^,^29^, of a hetero[4]rotaxane **R4**·4Cl, which contains pyrene stoppers and a diazaperopyrenium unit, derived from **1**·Cl and **2**·2Cl, respectively. Its fluorescent emission in the solid state can be fine-tuned rapidly and reversibly over a wide (∼100 nm) range of wavelengths as a result of stimuli-responsive aggregation and de-aggregation processes that are governed by a network of supramolecular equilibria. The unique features of this heterorotaxane include (i) the widely tunable colour of its fluorescent emission, providing a large analogue matrix of optical outputs, (ii) variations in fluorescent emission colour that occur when the heterorotaxane is deposited on different types of paper and (iii) the non-linear variation in its colour profile in response to simple chemical stimuli. These properties have allowed us to demonstrate a system of chemical encryption, in which the heterorotaxane is employed as a fluorescent security ink and its complex supramolecular equilibria serve as an encryption algorithm. By encrypting graphical information using these inks, printed images are nigh impossible to mimic, counterfeit and reverse engineer, yet can be easily verified on application of an appropriate authentication reagent, without revealing the original information.

## Results

### Synthesis of hetero[n]rotaxanes

**Figure 1:**
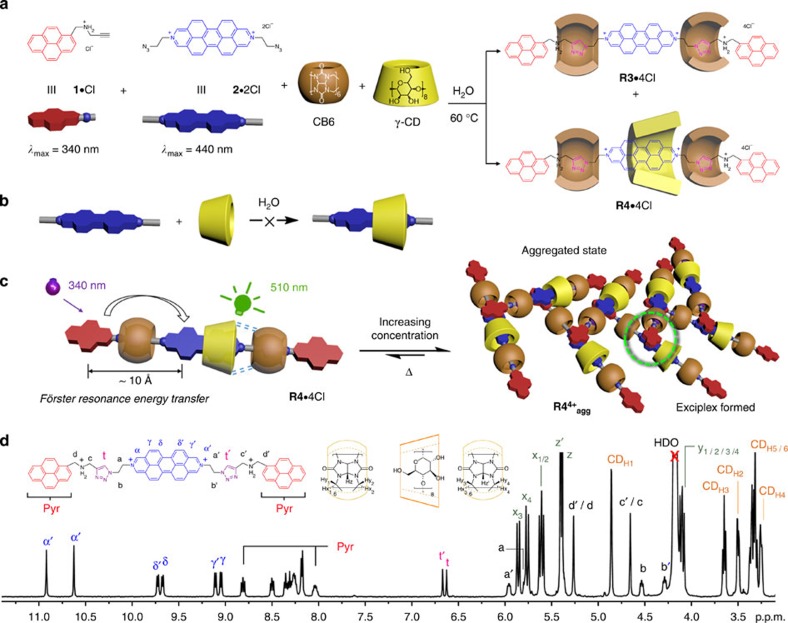
Synthesis and characterization of heterorotaxanes. (**a**) Synthesis of the heterorotaxanes **R3**·4Cl and **R4**·4Cl from the stopper **1**·Cl, the dumbbell precursor **2**·2Cl, CB6 and γ-CD. (**b**) No complexation was observed between **2**·2Cl and γ-CD. (**c**) Graphical representation of the aggregation of **R4**^4+^ monomers in response to changes in concentration or temperature. (**d**) ^1^H NMR spectrum (600 MHz) of **R4**·4Cl (1 mM) recorded in D_2_O at 80 °C.

The key compound in our investigations is the heterorotaxane **R4**·4Cl, which was isolated (Supplementary Figs 1−10) as an unexpected side product during the synthesis (Fig. 1a) of the heterorotaxane **R3**·4Cl (Supplementary Figs 11−14) from cucurbit[6]uril (CB6), γ-cyclodextrin (γ-CD) and two fluorescent precursors, one (**1**·Cl) derived from pyrene and the other (**2**·2Cl) from a diazaperopyrenium (DAPP) dication. In common with many fluorophores, DAPP exhibits a high fluorescence quantum yield^30^,^31^ in solution (*Φ*=53%) but not in the solid state (*Φ*=0%) as a result of aggregation-induced quenching. To ‘turn on' the fluorescence of DAPP in the solid state, we attempted to de-aggregate^32^ DAPP by encapsulating **2**·2Cl with γ-CD. No complexation, however, between **2**·2Cl and γ-CD was observed (Fig. 1b) in aqueous solution (Supplementary Figs 15−17). We anticipated that a more effective strategy would be to fix two bulky CB6 rings at the periphery of the DAPP dye as part of a mechanically interlocked molecule, formed by means of the highly efficient and rapid cooperative capture synthesis^27^,^28^,^29^ in aqueous solution. To our surprise, the reaction not only afforded the anticipated heterorotaxane **R3**·4Cl (yield=80%), but also another heterorotaxane **R4**·4Cl (yield=9%). The formation of **R4**·4Cl, in which γ-CD encircles the DAPP unit, seems to be at odds with the observation that γ-CD does not bind **2**·2Cl. The unfavourable energetics of threading γ-CD onto the dumbbell of the heterorotaxane are outweighed by the positive contribution from the hydrogen bonding network formed between γ-CD and the neighbouring CB6, an observation which is supported by molecular mechanics simulations (Supplementary Fig. 43), as well as the release of high-energy water molecules^33^ from the macrocycles. Increasing the amount of γ-CD to 10 equiv. in the reaction mixture favours the formation of **R4**·4Cl, which was isolated as the major product in 83% yield in 3 h (Supplementary Table 1). Although both heterorotaxanes dissolve to some extent in water, **R3**·4Cl, which lacks a solubilizing γ-CD ring, is poorly soluble and undergoes aggregation. In contrast, **R4**·4Cl exhibits significant water solubility of up to 3 mM at room temperature. ^1^H NMR spectroscopy reveals that, while **R4**·4Cl undergoes aggregation (Fig. 1c and Supplementary Figs 18−19) at room temperature, it experiences de-aggregation to its monomeric form (Fig. 1d) on heating to 80 °C. 2D-NOESY and variable temperature NMR experiments indicate (Supplementary Figs 20−21) that the γ-CD ring in **R4**·4Cl has a fixed orientation and position, and does not shuttle along the dumbbell rapidly on the ^1^H NMR timescale.

### Photophysical studies

**Figure 2:**
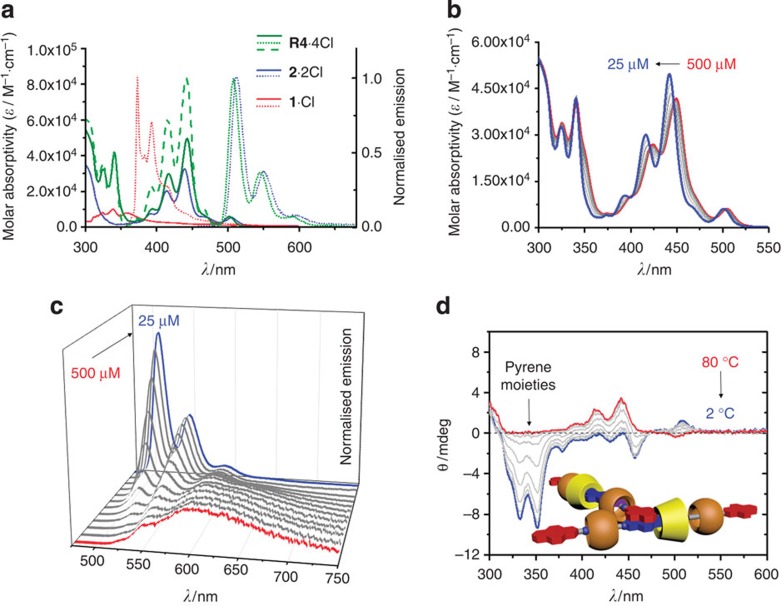
Photophysical studies of R4·4Cl. (**a**) UV/Vis absorption (solid lines) and normalised fluorescence spectra (excitation: dashed lines, emission: dotted lines) of aqueous solutions of **R4**·4Cl (green), stopper **1**·Cl (red) and dumbbell precursor **2**·2Cl (blue). (**b**) Concentration-dependent (25–500 μM) UV/Vis absorption spectra of **R4**·4Cl at 25 °C in water. (**c**) Normalised concentration-dependent (25–500 μM) fluorescence emission spectra (*λ*_excitation_=341 nm) of **R4**·4Cl at 25 °C in water. (**d**) Temperature-dependent (2–80 °C) ICD spectra (200 μM) of **R4**·4Cl in water.

The UV/Vis absorption spectrum (Fig. 2a) of **R4**·4Cl has two characteristic absorption bands at 341 and 443 nm, which can be attributed to electronic transitions in the pyrenyl and DAPP units, respectively. Despite the presence of the CB6 and γ-CD rings, **R4**·4Cl forms aggregates (**R4**^**4+**^_**agg**_) in water, as confirmed (Supplementary Fig. 22) by dynamic light scattering experiments. A blue shift (7 nm) of the absorption band near 450 nm is recorded (Fig. 2b) in the concentration-dependent UV/Vis absorption spectrum of **R4**·4Cl on dilution from 500 to 25 μM, while the shoulder evident at around 350 nm, arising from the pyrene stopper, diminishes. The isosbestic points observed (Fig. 2b) at 431 and 492 nm suggest^34^,^35^ that the aggregation is homogenous and non-cooperative^36^,^37^,^38^. By fitting the data to a dimerisation model^39^,^40^, the aggregation constant *K*_agg_ was determined (Supplementary Figs 23−25) to be 1.4 × 10^4^ M^−1^, indicating strong interactions between **R4**·4Cl monomers.

Irradiating a dilute solution (5 μM) of **R4**·4Cl at excitation wavelengths of either 340 or 443 nm results in identical fluorescence emission spectra (Supplementary Fig. 26), with an emission maximum (*Φ*=52.4%, Supplementary Table 2 and Supplementary Figs 27−44) at 510 nm. No emission is observed at 390 nm, indicating (Fig. 2a and Supplementary Fig. S41) the transfer of the excited state energy from pyrene to DAPP by a Förster resonance energy transfer (FRET) mechanism with near-quantitative (>99%) efficiency. This remarkable FRET efficiency can be rationalised by considering the geometric constraints enforced by the rings that impart (i) a close-to-ideal spatial separation (∼1.2 Å, calculated by molecular mechanics, Supplementary Figs 45−47) between the FRET donors (pyrene) and acceptor (DAPP) in **R4**·4Cl, while (ii) limiting conformational flexibility and (iii) preventing aggregation-induced quenching. These observations suggest that cooperative capture strategies have the potential to control the distances between fluorophores for applications such as bio-sensing^41^,^42^.

As **R4**·4Cl undergoes increased aggregation at higher and higher concentrations, its narrow emission band (Fig. 2c) at 510 nm is gradually replaced by a broad, featureless band around 610 nm, implying that either excimers (DAPP homodimers) or exciplexes (pyrenyl–DAPP heterodimers) are being formed in the excited state. Circular dichroism spectra reveal (Fig. 2d) that the aggregation of **R4**·4Cl (200 μM) is temperature dependent. As the temperature is lowered from 80 °C (monomeric state) to 2 °C (aggregated state), the positive induced circular dichroism (ICD) signals diminish, as a negative ICD peak^43^,^44^,^45^ attributable to the pyrene stoppers appears at around 350 nm, indicating that these stoppers congregate near the rims of the γ-CD ring. As γ-CD encircles DAPP, it follows that pyrene–DAPP heterodimers (Figs 1c and 2d), and their corresponding exciplexes, are responsible for the observed aggregation and emission behaviour of **R4**·4Cl.

### Dynamic supramolecular equilibria of the hetero[4]rotaxane

**Figure 3:**
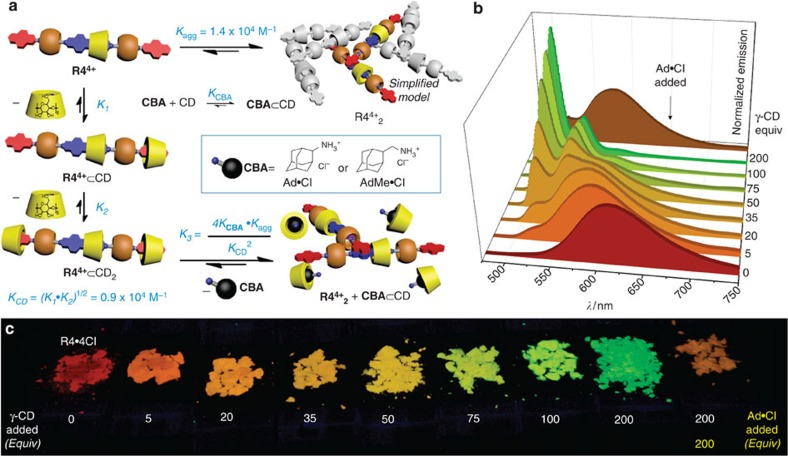
Equilibrium network and solid-state fluorescence studies. (**a**) Graphical representation of the equilibria involving **R4**^**4+**^ as its Cl^−^ salt in the presence of γ-CD and **CBA**s. (**b**) Solid-state fluorescence spectra (*λ*_excitation_=347 nm) of **R4**·4Cl on adding 0–200 equiv. of γ-CD, followed by 200 equiv. of Ad·Cl. (**c**) Powders obtained from homogeneous mixtures of **R4**·4Cl and varying amounts (0–200 equiv) of γ-CD and Ad·Cl (200 equiv) under UV light.

In aqueous solution, the disassembly (Fig. 3a) of aggregated heterorotaxanes (**R4**^**4+**^_**agg**_) is promoted by introducing γ-CD, which encircles the pyrene moieties of **R4**·4Cl and prevents aggregation. The stepwise encapsulation process, which occurs via the formation of a **R4**^**4+**^⊂CD intermediate, favours a monomeric **R4**^**4+**^⊂CD_2_ complex in the presence of excess of γ-CD, with an averaged equilibrium constant *K*_CD_=(*K*_1_·*K*_2_)^1/2^. As a result of the complex equilibria in solution, it is not easy to measure *K*_CD_ directly. To obtain a good estimate of the binding affinities between the pyrene moieties of **R4**·4Cl and γ-CD, a reference heterorotaxane **SR4**·4Cl was synthesised and the averaged binding affinity (0.9 × 10^4^ M^−1^) between its pyrene moiety and γ-CD was measured (Supplementary Figs 48−55) in D_2_O. This encapsulation process can be reversed (Fig. 3a) by introducing a competitive binding agent (**CBA**), which competes for γ-CD in solution with an association constant, *K*_**CBA**_. As the aggregation constant *K*_*ag*g_ and the encapsulation constant *K*_CD_ are of the same order of magnitude, addition of even a weak **CBA**, for example, 2-adamantylamine hydrochloride (Ad·Cl, *K*_**CBA**_=90 M^−1^, Supplementary Figs 56−59), will efficiently perturb the equilibria between **R4**^**4+**^_**agg**_, **R4**^**4+**^⊂CD_2_ and **R4**^**4+**^.

The dynamic nature of **R4**^**4+**^ in aqueous solution affords us the opportunity to customize an emission profile that remains preserved in the solid state after removal of the solvent. The fluorescent emission spectrum (Fig. 3b and Supplementary Figs 60−64) of the amorphous **R4**^**4+**^_**agg**_ (*λ*_max_=610 nm, *Φ*=7.7%) is very similar to its emission (Fig. 2c) in a concentrated aqueous solution (Supplementary Figs 65−66). On the addition of γ-CD, the solid-state fluorescent emission spectra become gradually blue-shifted to 510 nm (*Φ*=42.5%), with the emission colour (Fig. 3c) changing from red to green. Solid-state emission is also conserved from solution in the presence of a **CBA**. For example, the addition of 200 equiv. of Ad·Cl to a mixture comprising **R4**·4Cl:γ-CD (molar ratio: 1:200) results in a red-shift (Fig. 3b,c) of the emission back to *λ*_max_=580 nm. Thus, by changing the ratio of **R4**·4Cl, γ-CD and Ad·Cl, the solid-state fluorescence of the material can be tuned reversibly over a wide range of colours from green through to red. This stimulus-responsive tuning of fluorescence spectra in the solid state, over such a wide range of colours and under ambient conditions, is unique and holds promise for applications in security printing technology.

### Applications as fluorescent inks

**Figure 4:**
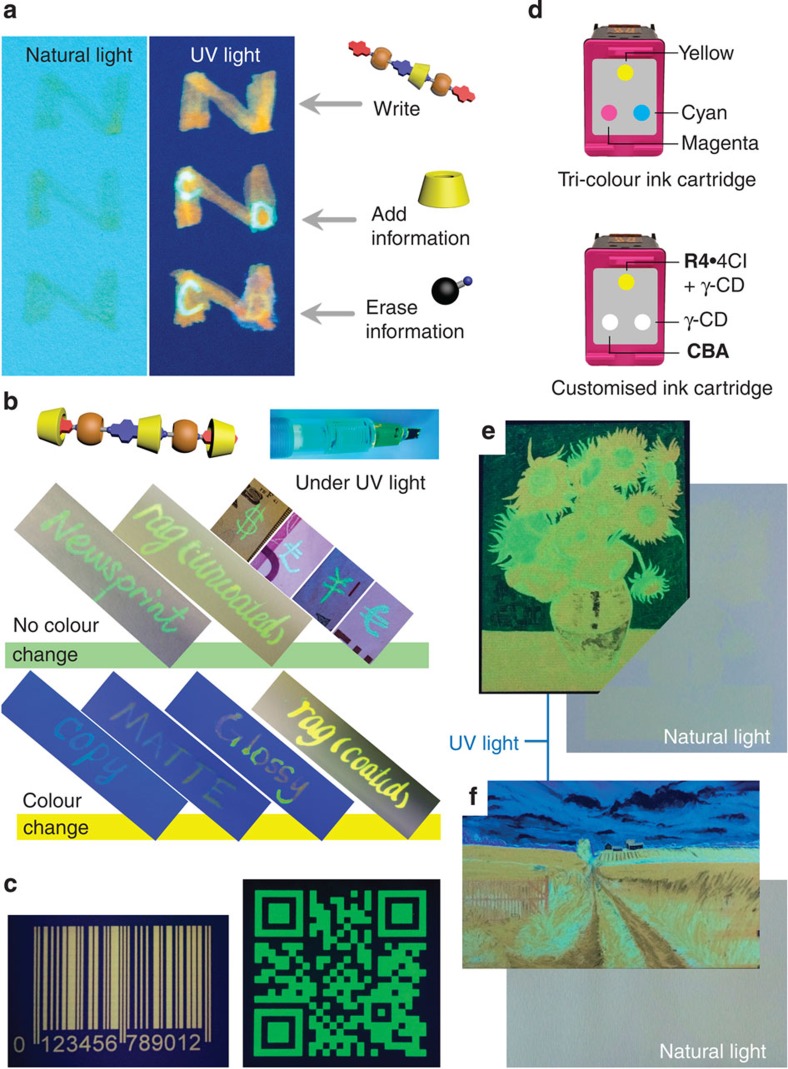
Security features of the heterorotaxane R4^4+^- and its complex R4^4+^⊂CD_2_-based fluorescent inks. (**a**) Reversibly adding and erasing information on the fluorescent ink with γ-CD and Ad·Cl aqueous solution. (**b**) Surface-dependent fluorescence of **R4**^**4+**^**⊂**CD_2_ ink on different paper media (newsprint, coated and uncoated rag paper, banknotes, copy, matte and glossy white paper) under UV light. (**c**) A UV barcode and a QR code under UV light printed using a customized black inkjet cartridge filled with **R4**^**4+**^ and **R4**^**4+**^⊂CD_2_ ink, respectively. (**d**) Graphical representations of a customized tri-colour inkjet cartridge, in which aqueous solutions of **R4**·4Cl/γ-CD (**R4**·4Cl: 1 mM, γ-CD: 200 mM), a **CBA** and γ-CD occupy the yellow, magenta and cyan colour channels, respectively. (**e**) Fluorescent replica of van Gogh's ‘Sunflowers' on rag paper printed using the customized tri-colour inkjet cartridge under UV and natural light. (**f**) Fluorescent image printed using an inkjet cartridge under UV and natural light, in which the cyan channel was loaded with γ-CD and PyMe·Cl.

By loading **R4**^4+^-based aqueous solutions (inks) into fountain pens, information can be written (Fig. 4), which is then revealed under UV light. By applying colourless γ-CD and Ad·Cl inks on top of the fluorescent **R4**·4Cl ink (Fig. 4a), additional information can be added (**R4**^**4+**^_**agg**_**→R4**^**4+**^⊂CD_2_) or erased (**R4**^**4+**^⊂CD_2_**→R4**^**4+**^_**agg**_) on pre-existing images that is only noticeable (Supplementary Fig. 67 and Supplementary Movie 1) under UV light. During handwriting experiments, we found that the **R4**^**4+**^⊂CD_2_ ink exhibits an unusual phenomenon, that is, the colour of its emission depends (Fig. 4b) on the type of paper (Supplementary Movie 2). For example, on rag paper, newsprint and banknotes, **R4**·4Cl appears reddish-orange and **R4**^**4+**^⊂CD_2_ appears green (Fig. 4b) under UV light, which is consistent (Fig. 3c) with the corresponding powders. On different kinds of ordinary white paper (Fig. 4b, Supplementary Figs 68−70 and Supplementary Table 3), however, both of these inks appear reddish-orange. The change of colour is most likely a result of noncovalent bonding interactions with papers of different compositions.

**R4**^**4+**^-based inks are also compatible with inkjet printing technology. A monochromic barcode and a QR code printed (Fig. 4c) on paper from an inkjet cartridge contains information that, although invisible under natural light, can be read (Supplementary Figs 71−72 and Supplementary Movie 3) on a smartphone under UV light. The supramolecular encapsulation/competition between **R4**^**4+**^, γ-CD and Ad·Cl is established rapidly (milliseconds) before the inks dry during the printing process, making it possible to print polychromic fluorescent images. By loading aqueous solutions of **R4**^**4+**^⊂CD_2_ (**R4**^**4+**^:γ-CD=1:50), γ-CD and Ad·Cl into a tri-colour inkjet cartridge (Fig. 4d and Supplementary Fig. 73), we have printed a fluorescent reproduction (Fig. 4e) of van Gogh's ‘Sunflowers' with good colour resolution. The colour range of the fluorescent inks can be expanded to accommodate RGB printing (Fig. 4f and Supplementary Figs 74-76) by choosing a fluorescent **CBA** with blue emission, such as the terminal fragment of **R4**·4Cl, 1-pyrenemethylamine hydrochloride (PyMe·Cl). It is worth noting that, by reducing the amount of **R4**·4Cl applied to the paper, the images produced are invisible to the naked eye (Fig. 4f, right) under natural light.

### Supramolecular encryption and authentication theory

**Figure 5:**
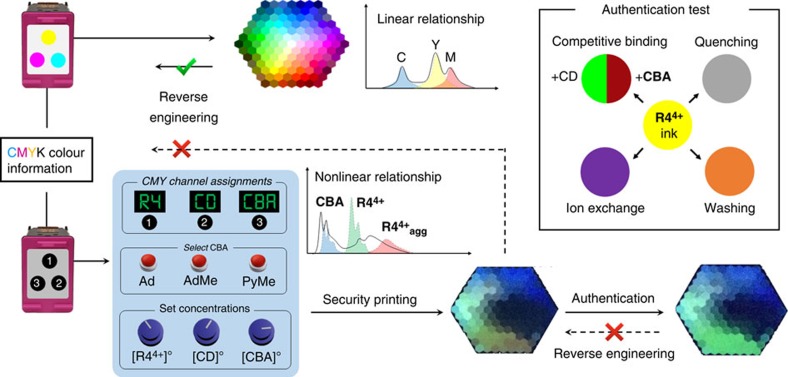
Supramolecular encryption and fraud detection using the heterorotaxane-based fluorescent security inks. A comparison between conventional cyan-magenta-yellow-black (CMYK) printing and supramolecular encrypted printing technology. Inset: possible mechanisms to verify the authenticity of the protected colour document.

At a fundamental level, the **R4**^**4+**^-based fluorescent inks provide an extensive fluorescent colour matrix for encryption coding. More importantly, the nonlinear dependence of this system's output on the concentrations of components and their equilibrium constants (Fig. 3a) points towards a general concept whereby complex supramolecular equilibria in aqueous solutions can be used as a chemical encryption method (Fig. 5). In principle, the colour of a dot printed by the customized tri-colour ink cartridge reflects a complex supramolecular equilibrium in the solution state, which can be simplified as


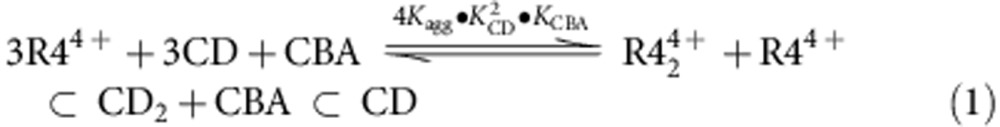


Since *K*_agg_ and *K*_CD_ are fixed, three key parameters control the supramolecular equilibria and the subsequent fluorescent colour (Fig. 5 and Supplementary Fig. 77) under UV light for a given dot after printing: (1) [**R4**^**4+**^]_0_, reflecting the absolute amount of **R4**^4+^ ink applied on paper, (2) [CD]_0_ and [**CBA**]_0_, reflecting the ratio of **R4**^**4+**^_**agg**_ and **R4**^**4+**^⊂CD_2_ on paper and (3) the chemical composition of **CBA**, reflecting a different *K*_**CBA**_ in the supramolecular equilibrium. These variables constitute the encryption settings, which can be defined by the user in charge of security printing. By simply varying (i) the sequence (Fig. 5) of inks in Channels , , , (ii) the chemical composition of the **CBA** and (iii) the concentration of the inks, it is possible to generate a large number of fluorescent colour combinations. It is also worth noting that, a combination of different **CBA**s could be loaded in channels and simultaneously, thus introducing even more variables to the supramolecular equilibria. In this manner, it would be challenging for counterfeiters to reproduce a printed colour palette even if they had access to the **R4**·4Cl ink, as they would also require a complete knowledge of (i) the **CBA**, of which there could be a large number of possibilities, as well as (ii) the paper media, (iii) channel assignments and (iv) initial ink concentrations. Even relatively small errors in initial concentrations can lead to obvious differences in the colour palette, by a margin which cannot be easily reverse engineered on account of the non-linearity of the equilibrium equations,


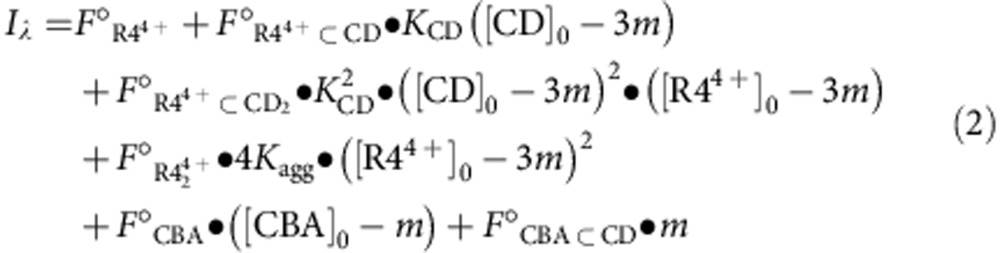


where the fluorescence intensity *I*_λ_ at a given wavelength *λ* is the sum of the emission intensities of each component in an aqueous solution containing **R4**·4Cl, γ-CD and **CBA**. *F*° is the molar fluorescence coefficient at wavelength *λ*, and *m* is the molar concentration of **CBA** being encapsulated by γ-CD. See Supplementary Discussion for detailed derivations.

The dynamic nature of the inks also makes them amenable to a variety of fraud detection mechanisms. In principle, applying a layer of authentication agent(s) can re-establish the supramolecular equilibria in solution and shift the colour outputs (Fig. 5) on paper non-linearly, as described by equations (1) and (2). As the colour-changing process is dynamic and depends on the amount of the authentication agent(s) that has been applied, it is close to impossible that the dynamic colour-changing process could be precisely mimicked. Apart from applying authentication agent(s), which could induce a supramolecular equilibrium shift, mechanisms such as counterion exchange and fluorescence quenching can also be utilized (Fig. 5 inset) to change emission colours in real time.

### Demonstration of the supramolecular encryption theory

**Figure 6:**
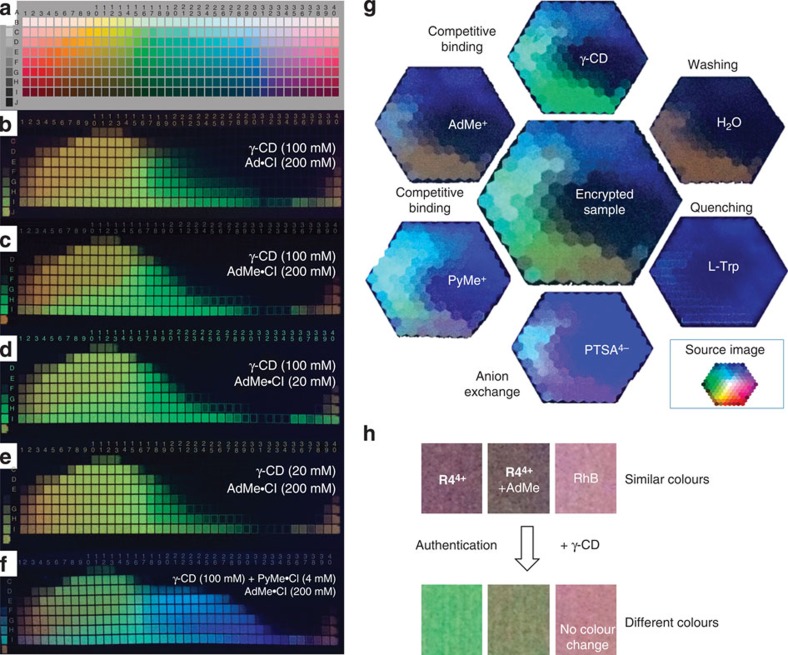
Demonstration of the supramolecular encryption and authentication using the heterorotaxane-based fluorescent security inks. (**a**) A standard colour palette. (**b**–**f**) Colour palette images produced using the customized tri-colour inkjet cartridge with (**b**) Ad·Cl (200 mM), (**c**) AdMe·Cl (200 mM) and (**d**) AdMe·Cl (20 mM) in channel , (**e**) γ-CD (20 mM) and (**f**) γ-CD (100 mM) with PyMe·Cl (4 mM) in channel , respectively. **R4**·4Cl+γ-CD (**R4**·4Cl: 1 mM, γ-CD: 40 mM) solution was loaded in channel in the tri-colour inkjet cartridge. (**g**) Encrypted polychromic colour palette samples produced by the customized inkjet cartridge (centre) and its derivatives (around the periphery, after printing a layer of authentication reagents) under UV light. (**h**) Similar colours produced by **R4^4+^**-based security inks have composition-dependent response after chemical authentication. No distinguishable colour change is observed after chemical authentication when rhodamine B (RhB) is applied as the fluorescent ink.

In an attempt to demonstrate the non-linear nature of this system, we have printed fluorescent colour palettes (Fig. 6a−f) using various ink concentrations and different **CBA**s. A broad range of colours from green to red can be printed (Fig. 6b) when Ad·Cl is used as the competitor. The colour palette is sensitive to the association strength of the competitor as predicted and evidenced by the differences between images printed from equally concentrated solutions of Ad·Cl (Fig. 6b) and a stronger-binding competitor (Fig. 6c), namely, 1-adamantanemethylamine hydrochloride (AdMe·Cl, *K*_AdMe_=127 M^−1^, Supplementary Figs 58−59). Reducing the concentrations of either **CBA** (Fig. 6c,d) or γ-CD (Fig. 6c,e) redistributes the colour spectrum in the yellowish-red region or greenish yellow region, respectively. The colour spectrum is expanded (Fig. 6f) by choosing a blue fluorescent **CBA**, for example, PyMe·Cl. The ability to exchange fluorescent and non-fluorescent **CBA**s in a modular and user-controlled manner elevates the anti-counterfeiting features possessed intrinsically by this new security ink.

### Demonstration of authentication methods

Authentication mechanisms have also been demonstrated. For example, exposure of an encrypted image (Fig. 6g, centre) to aqueous solutions of non-fluorescent AdMe·Cl or γ-CD changes the existing colour gradient of the image, while printing fluorescent PyMe·Cl aqueous solution creates new colours, such as blue and purple by shifting the complex equilibria. Printing 1,3,6,8-pyrenetetrasulfonic acid tetrasodium (PTSA·4Na) solution gives rise to new colours (Fig. 6g) as a result of counterion exchange. A characteristic colour change can also be brought about through the application of a quencher, such as tryptophan, or even by simply soaking the printed image in water for as little as 1−2 min, during which time γ-CD and the **CBA** are washed away to some extent, thus shifting the equilibria. These authentication agents can discriminate, not only between images produced by the **R4**^**4+**^-based ink and other fluorescent dyes, but also between images produced using different **R4**^**4+**^/**CBA/**γ-CD ink formulations. In a blind test, blocks of a given fluorescence colour (Fig. 6h, top), which appear almost identical to one another under UV light, but are formulated differently, were found to result in noticeably different colours (Fig. 6h bottom) after the application of the same amount of authentication agents. For further details, see Supplementary Fig. 78. As hundreds of chemicals meet the criteria to be **CBA**s, an extremely large library of different ink systems and authentication tests using this supramolecular encryption method can, in principle, be generated.

## Discussion

In summary, we have developed a stimulus-responsive solid-state fluorescent heterorotaxane, which is easily prepared from simple starting materials in high yield by a cooperative capture method. We have applied it as a component of fluorescent security inks with built-in supramolecular encryption. The inks are well-placed for assimilation into a commercial setting on account of the simple and high-yielding synthesis of the heterorotaxane from commodity chemicals. The solid-state emission of these security inks can be fine-tuned over a wide emission range (∼100 nm) with rapid response (milliseconds) to chemical stimuli. Printed information is encrypted in a chemical language based on a nonlinear equation that describes the dynamic equilibrium network. A potentially enormous library of different fluorescent colour combinations can be generated. In contrast with conventional dyes, the encrypted information printed using the heterorotaxane inks can be verified by chemical authentication methods without revealing the original colour image information. The interplay of fluorescence output with dynamic supramolecular equilibria that we observed quite fortuitously could be a general phenomenon that is not exclusive to the heterorotaxane, or even to mechanically interlocked molecules. It opens up a new way to encrypt and protect information in a manner that is far from easy to mimic.

## Methods

### Synthesis and characterization of the heterorotaxane R4·4Cl

Stopper precursor **1**·Cl (67 mg, 0.22 mmol), dumbbell precursor **2**·2Cl (54 mg, 0.10 mmol) and γ-CD (1287, mg, 1.00 mmol) were mixed in H_2_O (35 ml) and stirred at 60 °C for 10 min before CB6 (250 mg, 0.25 mmol) was added. The reaction mixture was stirred at 60 °C for 3 h. Insoluble residues were filtered off from the reaction mixture. The filtrate was loaded directly onto a reverse phase C18 column (150 gram, RediSep Rf Gold C18Aq) on an automatic column chromatographic system (Combiflash Rf200, Teledyne Isco) and chromatographed in H_2_O/MeCN/0.1% TFA with a gradient from 0 to 60% MeCN over 40 min at a flow rate of 85 ml min^−1^. Fractions containing **R4**^**4+**^ were collected and the counterions of **R4**^**4+**^ were exchanged to PF_6_^−^ on addition of an excess of aqueous NH_4_PF_6_. The product **R4**·4PF_6_, which precipitates on removal of MeCN from these fractions under reduced pressure, was collected by vacuum filtration and washed extensively with H_2_O. The hetero[4]rotaxane **R4**·4Cl was obtained after a second counterion exchange by precipitation from an MeCN solution of **R4**·4PF_6_ with an excess of tetrabutylammonium chloride. The yellow precipitate was collected by vacuum filtration, washed with excess MeCN and dried under vacuum to afford **R4**·4Cl (371 mg, 83%) as an orange powder. ^1^H NMR (600 MHz, D_2_O, 353 K): δ=10.92 (s, 2H), 10.63 (s, 2H), 9.73 (d, *J*=9.5 Hz, 2H), 9.68 (d, *J*=9.4 Hz, 2H), 9.11 (d, *J*=9.3 Hz, 2H), 9.05 (d, *J*=9.3 Hz, 2H), 8.81 (dd, *J*=12.4, 9.3 Hz, 2H), 8.50 (dd, *J*=11.5, 7.9 Hz, 2H), 8.40–8.22 (m, 8H), 8.18 (d, *J*=8.9 Hz, 4H), 8.08–7.98 (m, 2H), 6.67 (s, 1H), 6.63 (s, 1H), 5.96 (t, *J*=8.8 Hz, 2H), 5.86 (d, *J*=15.3 Hz, 6H), 5.80 (m, 2H), 5.76 (d, *J*=15.5 Hz, 6H), 5.62 (d, *J*=15.5, 6H), 5.60 (d, *J*=15.5, 6H), 5.40 (s, 12H), 5.39 (s, 12H), 5.26 (s, 4H), 4.86 (d, *J*=3.8 Hz, 8H), 4.66 (s, 4H), 4.53 (t, *J*=9.0 Hz, 2H), 4.29 (t, *J*=8.9 Hz, 2H), 4.10 (m, 24H), 3.65 (t, *J*=9.5 Hz, 8H), 3.50 (dd, *J*=10.0, 3.8 Hz, 8H), 3.41–3.20 (m, 32H). HR-ESI-MS: calcd for [*M* − 4Cl]^4+^ *m/z*=1,074.8630, found *m/z*=1,074.8623; [*M* − H − 4Cl]^3+^ *m/z*=1,432.8149, found *m/z*=1,432.8123; [*M* − 3Cl]^3+^ *m/z*=1,445.1406, found *m/z*=1,445.1369.

Detailed synthesis and characterisation of stopper and rod precursors, rotaxanes **R3**·4Cl and **SR4**·4Cl are available in Supplementary Methods.

### Photophysical studies of the heterorotaxane R4·4Cl

The UV/Vis spectra of the sample solutions were measured on a Shimadzu UV/Vis/NIR spectrometer (UV 3600 model) with a cell temperature controller. Quartz cuvettes with 1 or 10 mm pathway were used to record the UV/Vis spectra. The fluorescence excitation and emission spectra of the sample solutions were recorded on a HORIBA fluorometer (fluoroMax-4 model). Circular dichroism spectra were recorded on a JASCO circular dichroism spectrophotometer (J-815 model) with a temperature controller. Solid-state UV/Vis spectra were recorded on a Perkin Elmer UV/Vis/NIR spectrometer (LAMBDA 1050 model) equipped with an integrating sphere. Solid-state fluorescence spectra were recorded on an ISS fluorometer (PC1 model) equipped with a variable-angle, front surface sample compartment.

### Ink writing tests

Four types of inks for pen writing were prepared using **R4**·4Cl (0.5 mM), Ad·Cl (100 mM), γ-CD (100 mM) and **R4**⊂CD_2_ (**R4**·4Cl=0.5 mM, γ-CD=100 mM) solutions, respectively. Typically, 0.5 ml of the ink was loaded in a fountain pen for writing tests. A wide selection of paper-based printing media has been tested, including copy papers (various brands and models), matte presentation paper (HP), glossy presentation paper (HP), resume paper (25 and 100% cotton), newsprint paper, rag paper (100% cotton, without optical brightener) and cigarette rolling paper. Banknote identification tests were performed on genuine banknotes of US dollars, British pounds sterling, Euros, Chinese Yuan and Japanese Yen. In these tests, the corresponding currency symbols ($, £, € and ¥) were drawn on the testing banknotes using the fountain pen filled with **R4**⊂CD_2_ ink.

### Ink printing tests

Printing tests were performed on an HP inkjet printer (Photosmart CP4780 model) and an HP colour laser printer (CP1025nw model) with customized ink cartridges and original toners, respectively. Rag paper (100% cotton, without optical brightener, no surface coating side) was chosen for most printing tests based on the ink writing test results.

Ink cartridges for printing tests were customized from HP black and tri-colour cartridges (HP60 model). The filled inks were removed from the cartridge, which was washed extensively with H_2_O and EtOH. Aqueous solutions of **R4**·4Cl (4 mL, 0.5 mM) and **R4**⊂CD_2_ (4 ml, **R4**·4Cl=0.5 mM, γ-CD=100 mM) were loaded in two empty, clean black ink cartridges, respectively, to perform the monochromic printing tests. In the polychromic printing tests, aqueous solutions of Ad·Cl (2 ml, 100 mM), **R4**/γ-CD (2 ml, **R4**·4Cl=0.5 mM, γ-CD=25 mM) and γ-CD (2 ml, 100 mM) were loaded in the magenta, yellow and cyan channels of the cleaned tri-colour ink cartridge, respectively. Fluorescent colour under UV light was tuned by controlling the proportion of the three inks in the customized tri-colour ink cartridge.

## Author contributions

C.K. and X.H. conceived the project, X.H. and C.K. performed the experiments and analysed the data under the direction of J.F.S., C.J.B. and P.R.M. gave suggestions to optimize the system, R.B.P. suggested the potential application as ink, and all authors contributed in the manuscript preparation.

## Additional information

**How to cite this article:** Hou, X. *et al*. Tunable solid-state fluorescent materials for supramolecular encryption. *Nat. Commun.* 6:6884 doi: 10.1038/ncomms7884 (2015).

## Supplementary Material

Supplementary Figures, Supplementary Tables, Supplementary Discussion, Supplementary Methods and Supplementary References Supplementary Figures 1-76, Supplementary Tables 1-3, Supplementary Discussion, Supplementary Methods and Supplementary References

Supplementary Movie 1 Fluorescence colour changing and recovery of "NU" written with **R4**^**4+**^⊂CD_2_ ink demonstrated by applying an aqueous solution of Ad·Cl and γ-CD consecutively. An inexpensive UV-LED light is used as the light source in the movie.

Supplementary Movie 2 Fluorescence colour changing of the **R4**^**4+**^⊂CD_2_ ink applied on different types of paper. An inexpensive UV-LED light is used as the light source in the movie.

Supplementary Movie 3 Fluorescent UV barcode and QR code produced using the **R4**^**4+**^⊂CD_2_ ink, which could be recognised by a smart phone camera only under UV light.
