# Supplementary material for: Tunable solid-state fluorescent materials for supramolecular encryption
Source: Nat Commun. 2015 Apr 22;6:6884. doi: 10.1038/ncomms7884 (PMC4423226; doi:10.1038/ncomms7884)
Supplement: Supplementary Figures, Supplementary Tables, Supplementary Discussion, Supplementary Methods and Supplementary References — Supplementary Figures 1-76, Supplementary Tables 1-3, Supplementary Discussion, Supplementary Methods and Supplementary References [file ncomms7884-s1.pdf]

Supplementary Figures

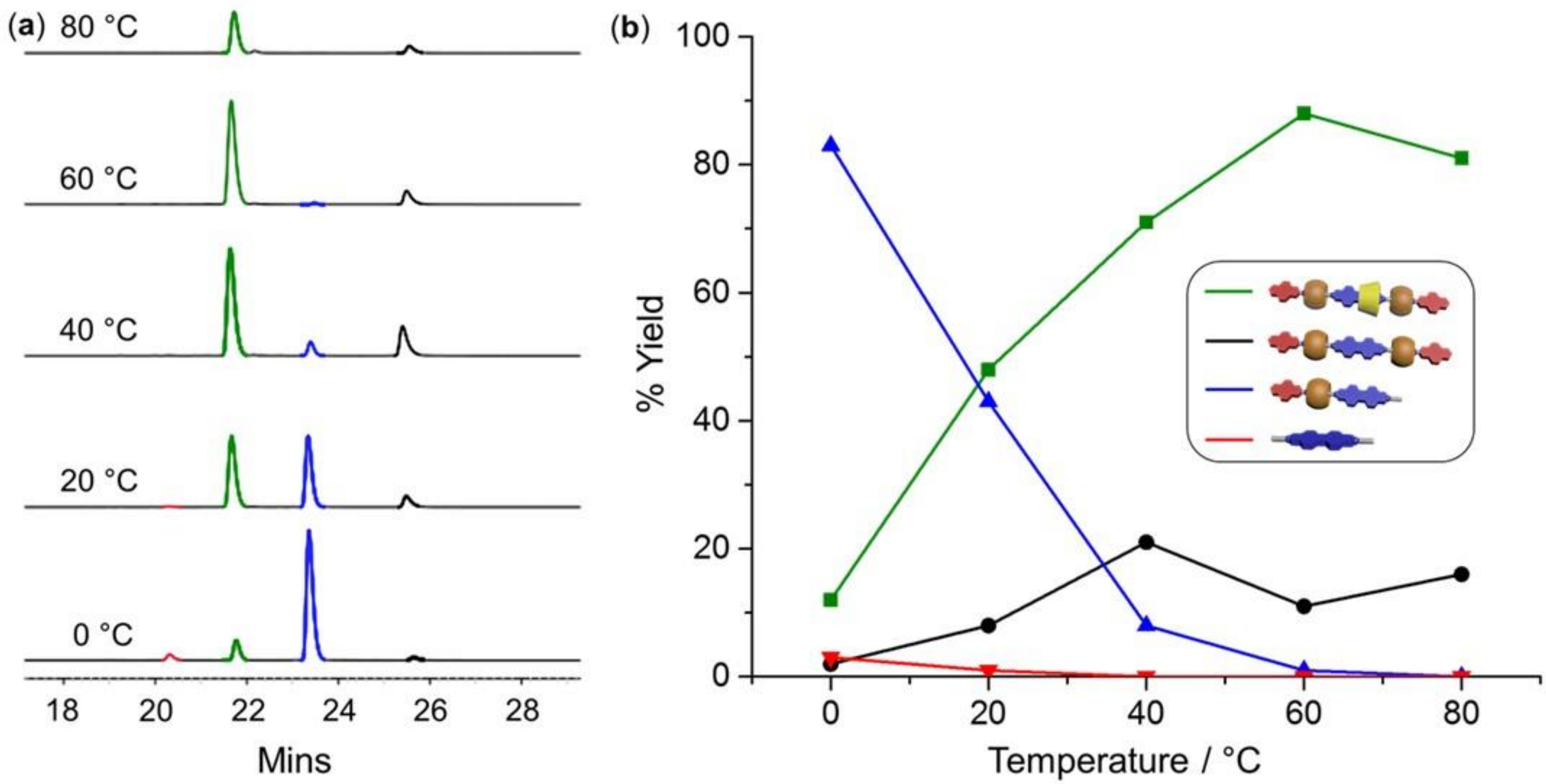

Supplementary Figure 1. (a) Reverse-phase HPLC profiles and (b) the percentage yields against-temperature diagram for the synthesis of the [4]rotaxane **R4**•4Cl at different temperatures.

NMR Spectroscopy and Mass Spectrometry

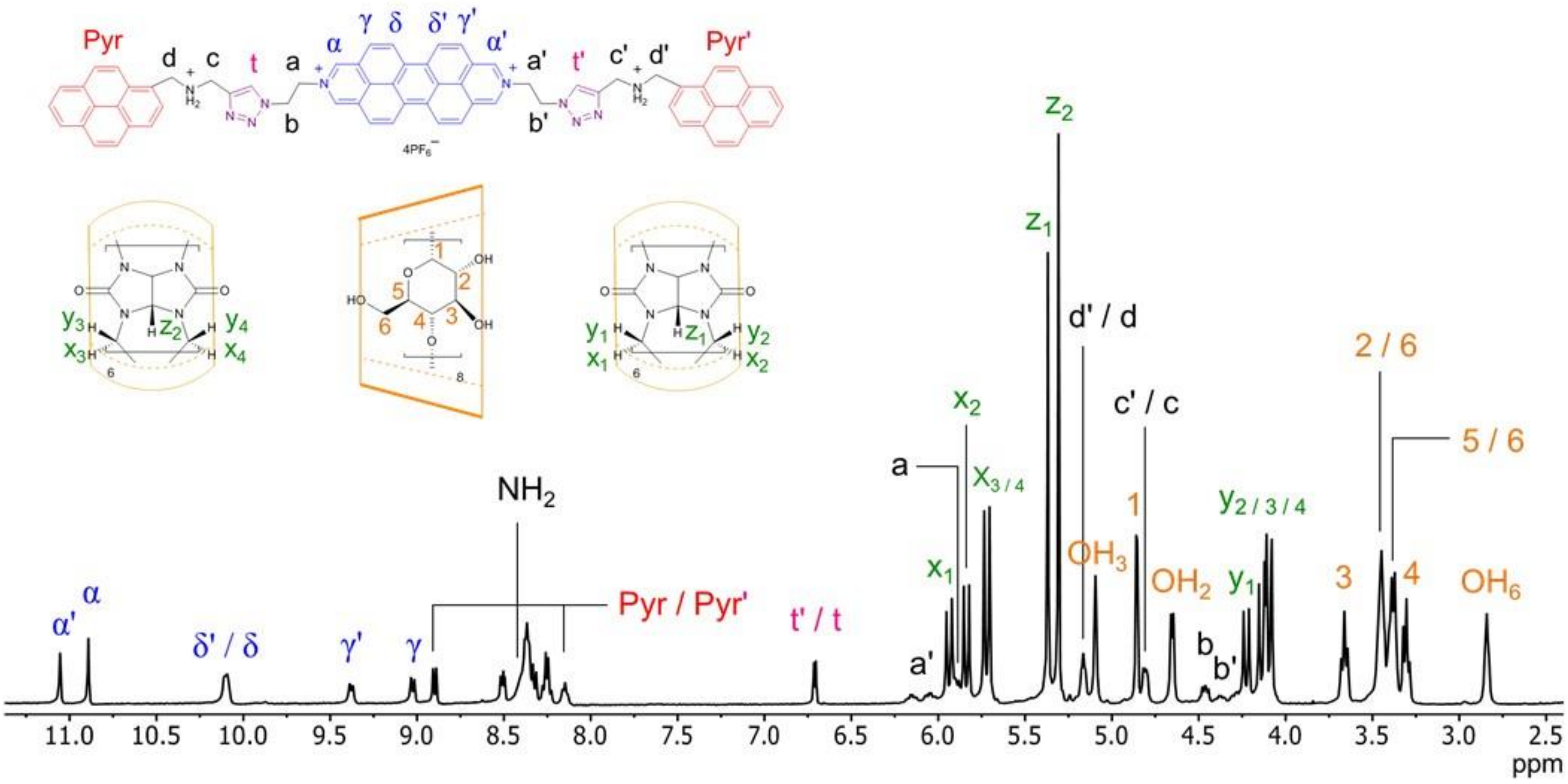

Supplementary Figure 2. ^{1}H NMR spectrum (500 MHz, CD_3CN, 25 °C) of **R4•**$4PF_6$. Rather than illustrate the structural formula of **R4•**$4PF_6$, its components with labeled protons are shown above.

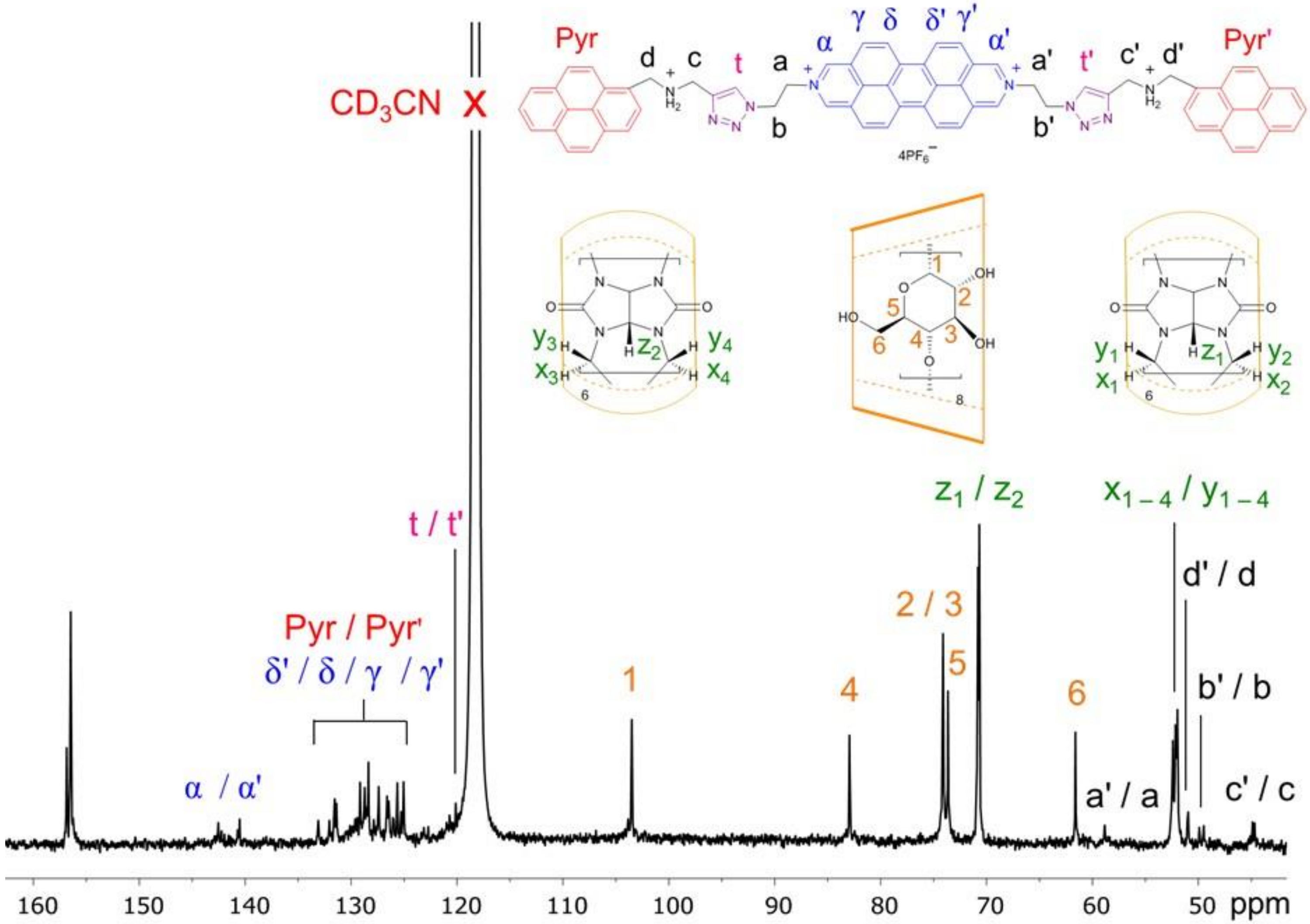

Supplementary Figure 3. ^{13}C NMR spectrum (500 MHz, CD_3CN, 25 °C) of **R4•**$4PF_6$. Rather than illustrate the structural formula of **R4•**$4PF_6$, its components with labeled protons are shown above.

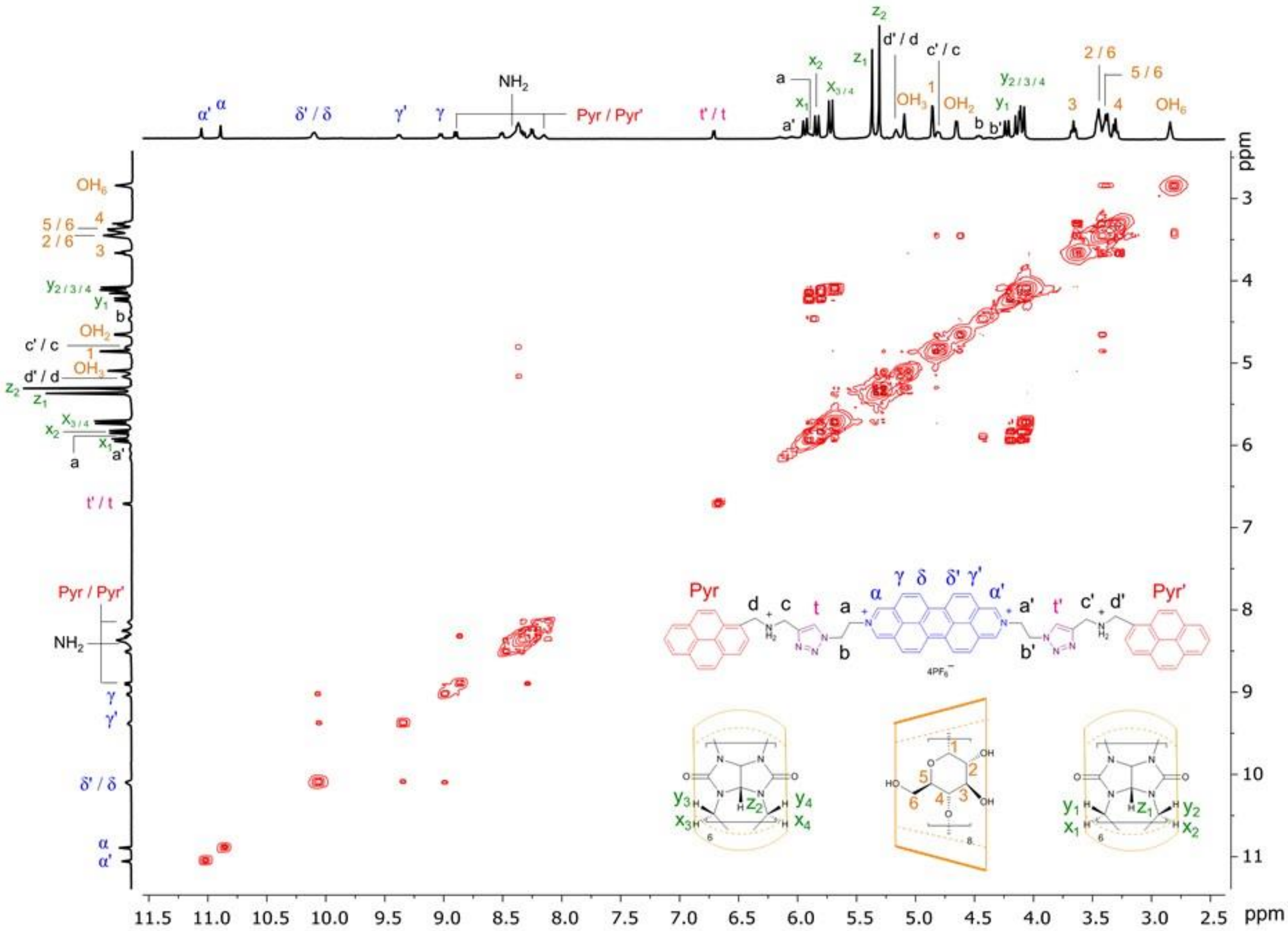

Supplementary Figure 4. ^{1}H-^{1}H COSY spectrum of **R4•**$4PF_6$ (500 MHz, CD_3CN, 25 °C). Rather than illustrate the structural formula of **R4•**$4PF_6$, its components with labeled protons are shown above.

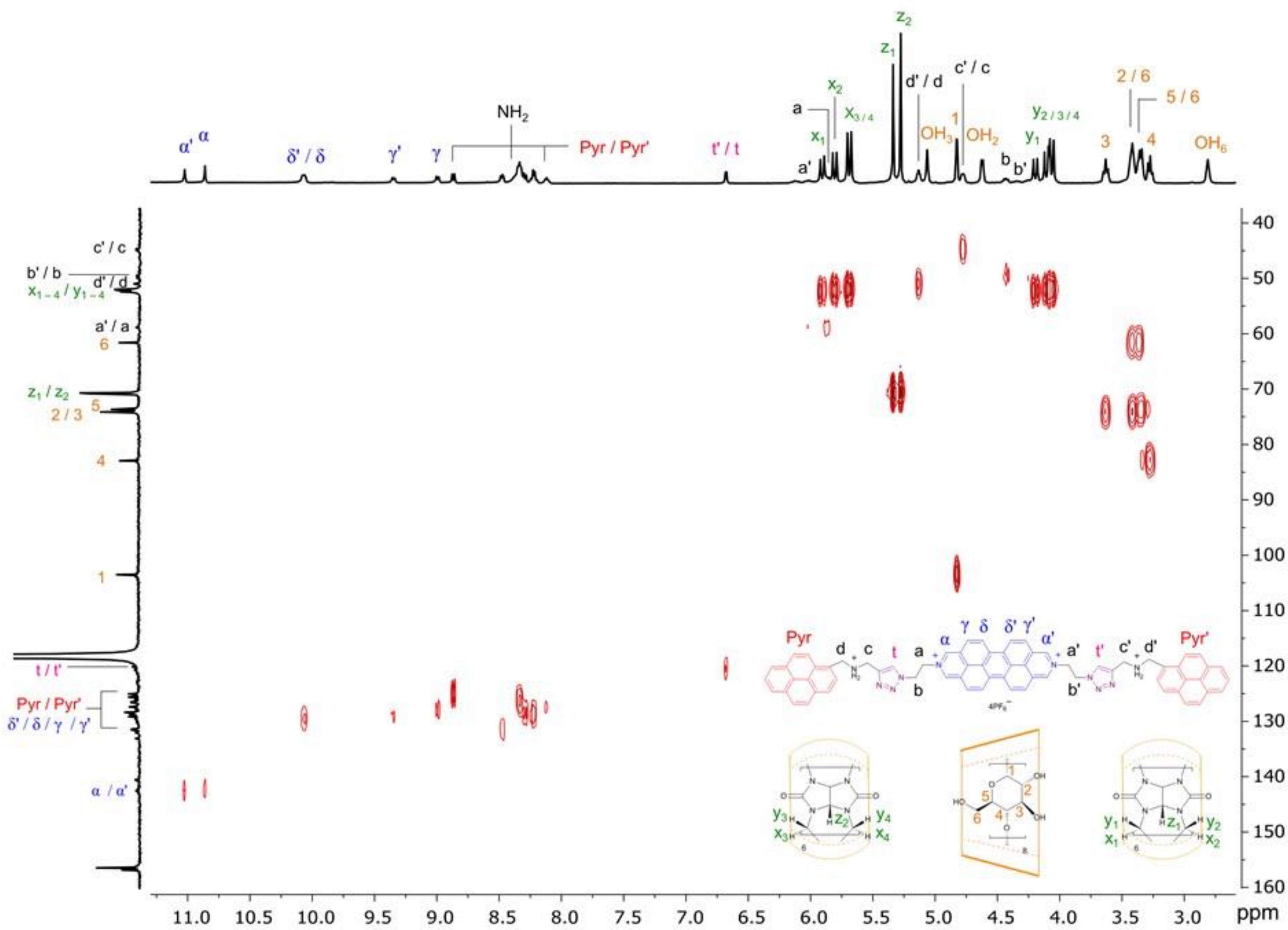

Supplementary Figure 5. ^1H-^{13}C HSQC spectrum of **R4•**4PF$_6$ (500 MHz, CD$_3$CN, 25 °C). Rather than illustrate the structural formula of **R4•**4PF$_6$, its components with labeled protons are shown above.

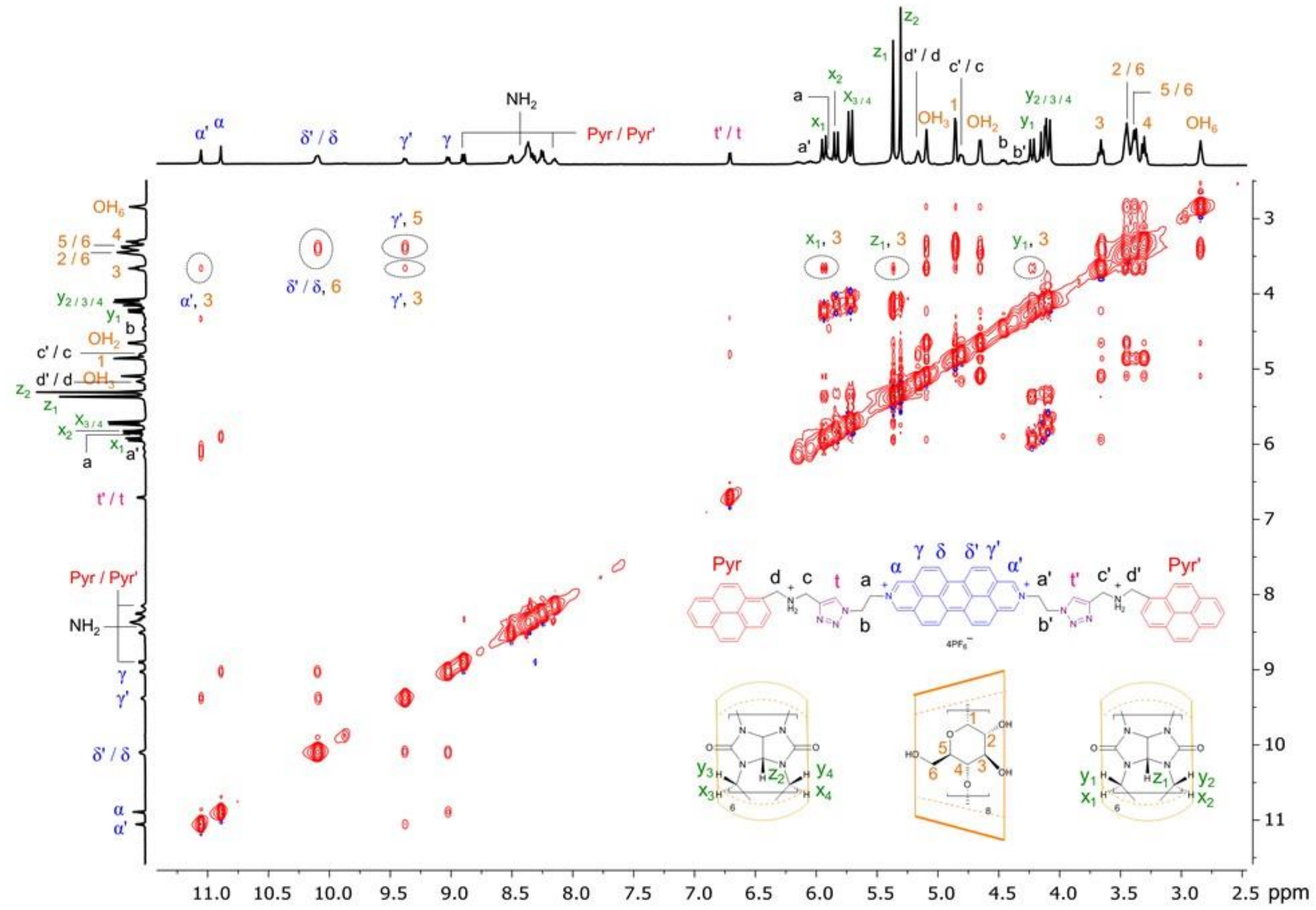

Supplementary Figure 6. ^{1}H-^{1}H NOESY spectrum of **R4•**4PF$_6$ (500 MHz, CD_3CN, 25 °C). Rather than illustrate the structural formula of **R4•**4PF$_6$, its components with labeled protons are shown above.

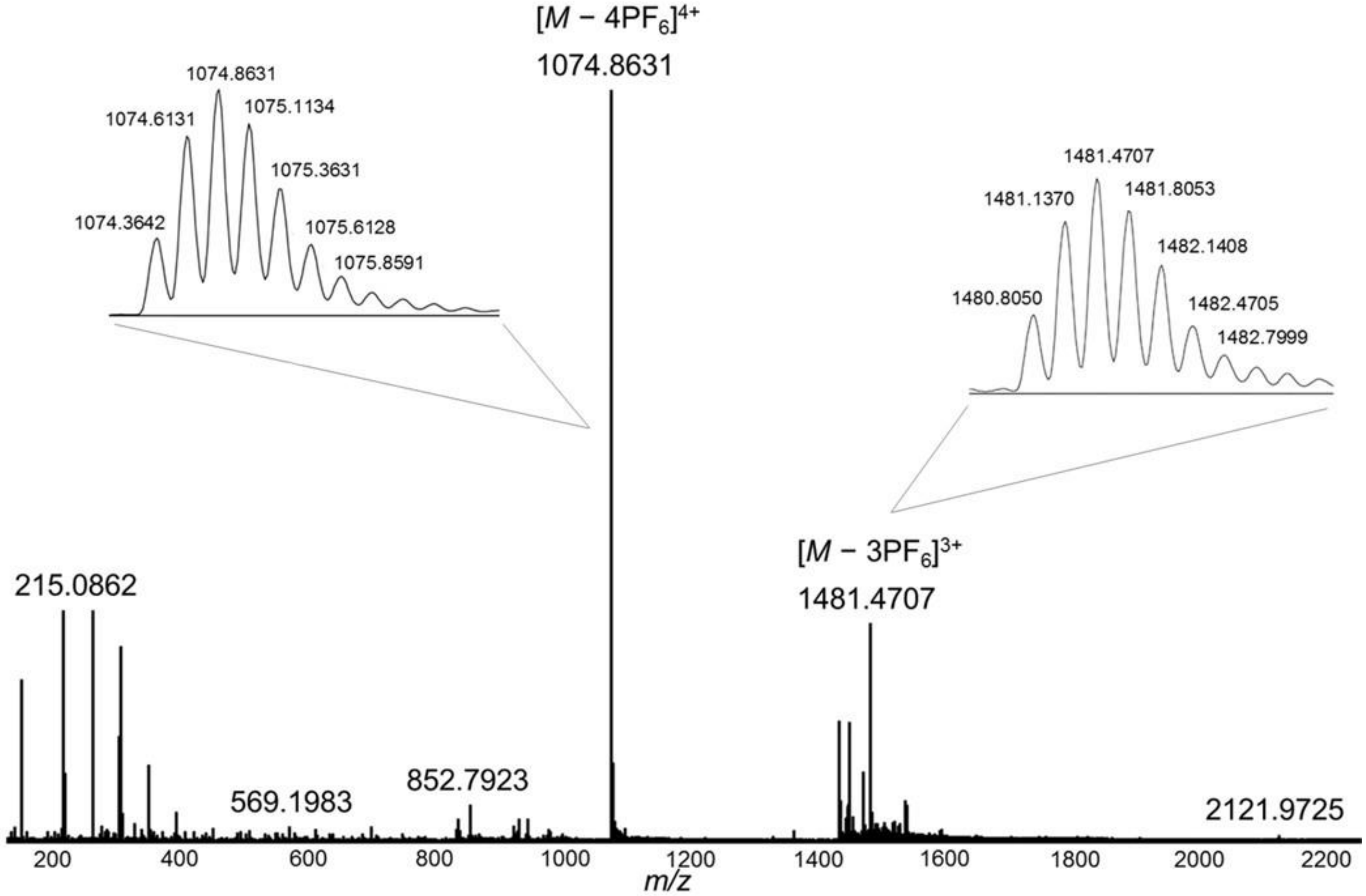

Supplementary Figure 7. High resolution ESI mass spectrum of **R4•**$4PF_6$

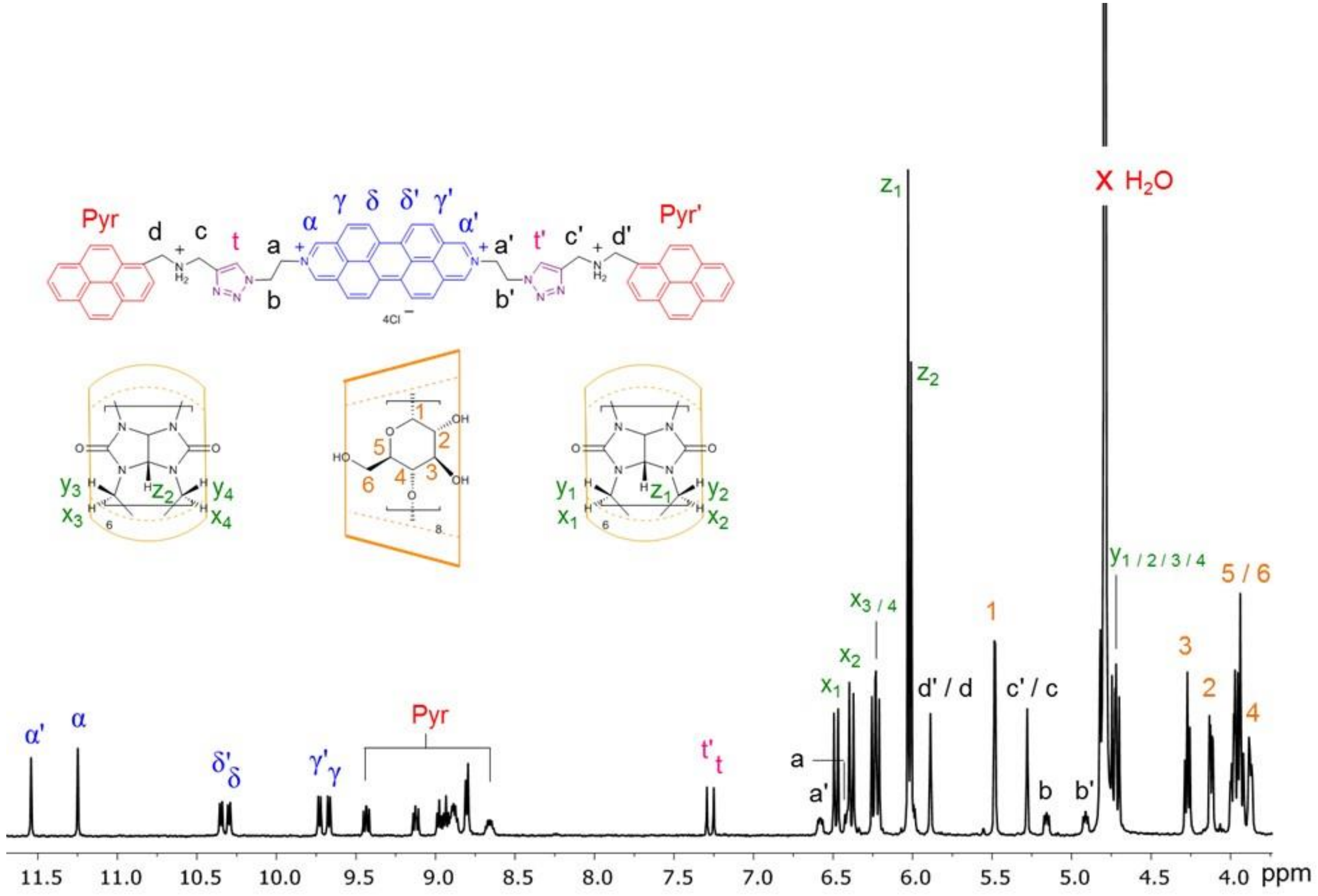

Supplementary Figure 8. ^{1}H NMR spectrum (600 MHz, D_2O, 80 °C) of **R4•**4Cl. Rather than illustrate the structural formula of **R4•**4Cl, its components with labeled protons are shown above.

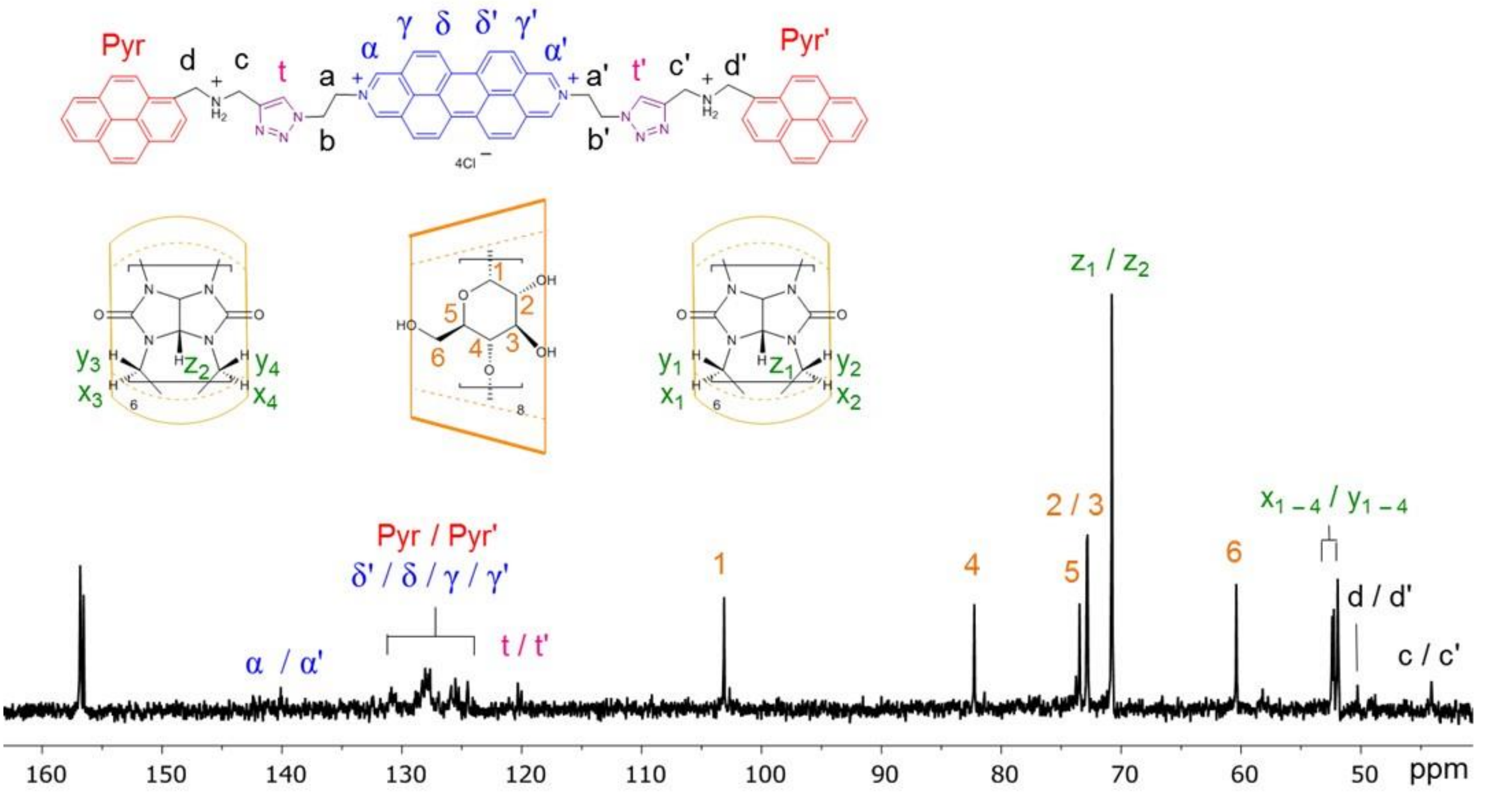

Supplementary Figure 9. ^{13}C NMR spectrum (600 MHz, D_2O, 80 °C) of **R4•**4Cl. Rather than illustrate the structural formula of **R4•**4Cl, its components with labeled protons are shown above.

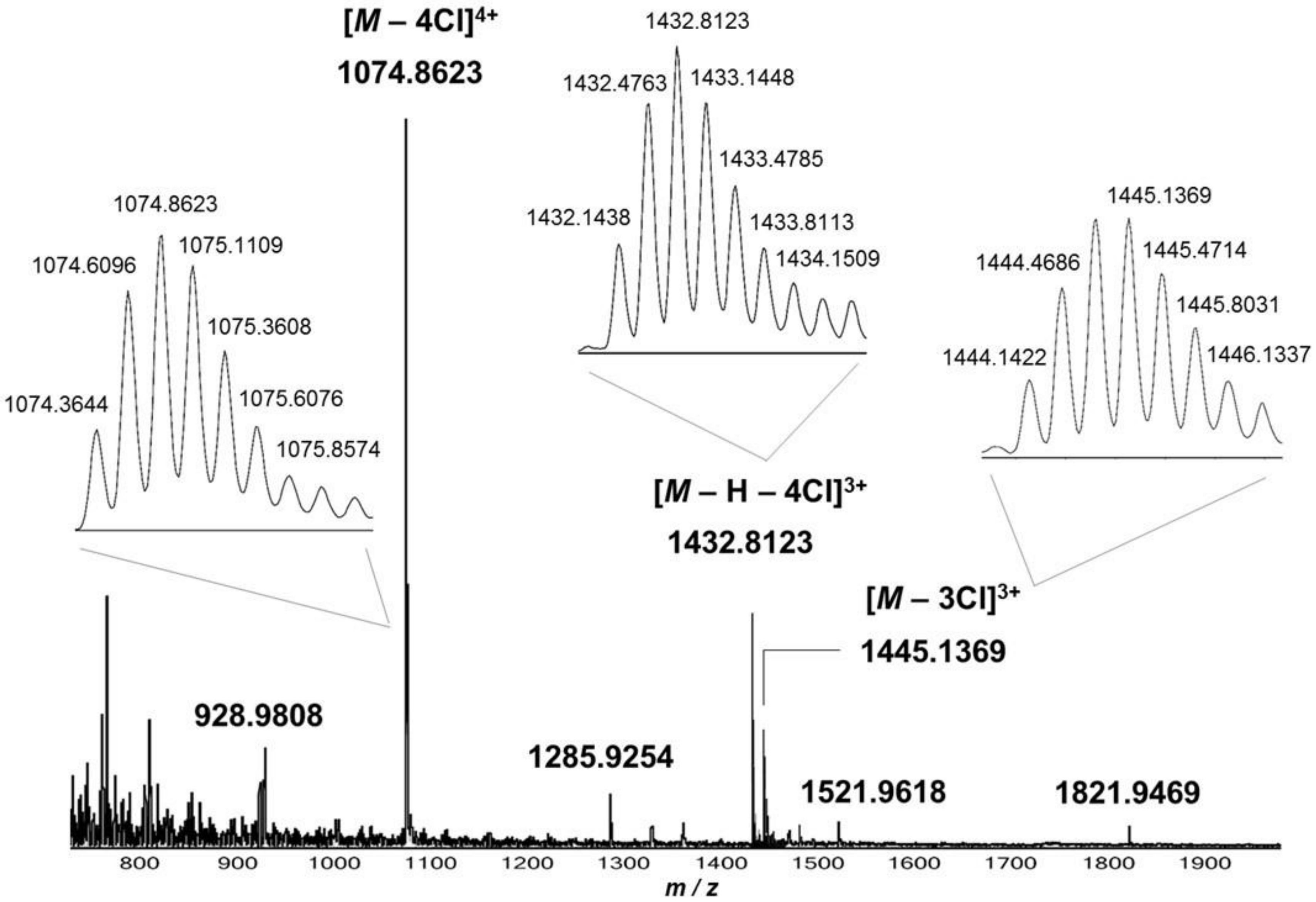

Supplementary Figure 10. High resolution ESI mass spectrum of **R4•**4Cl

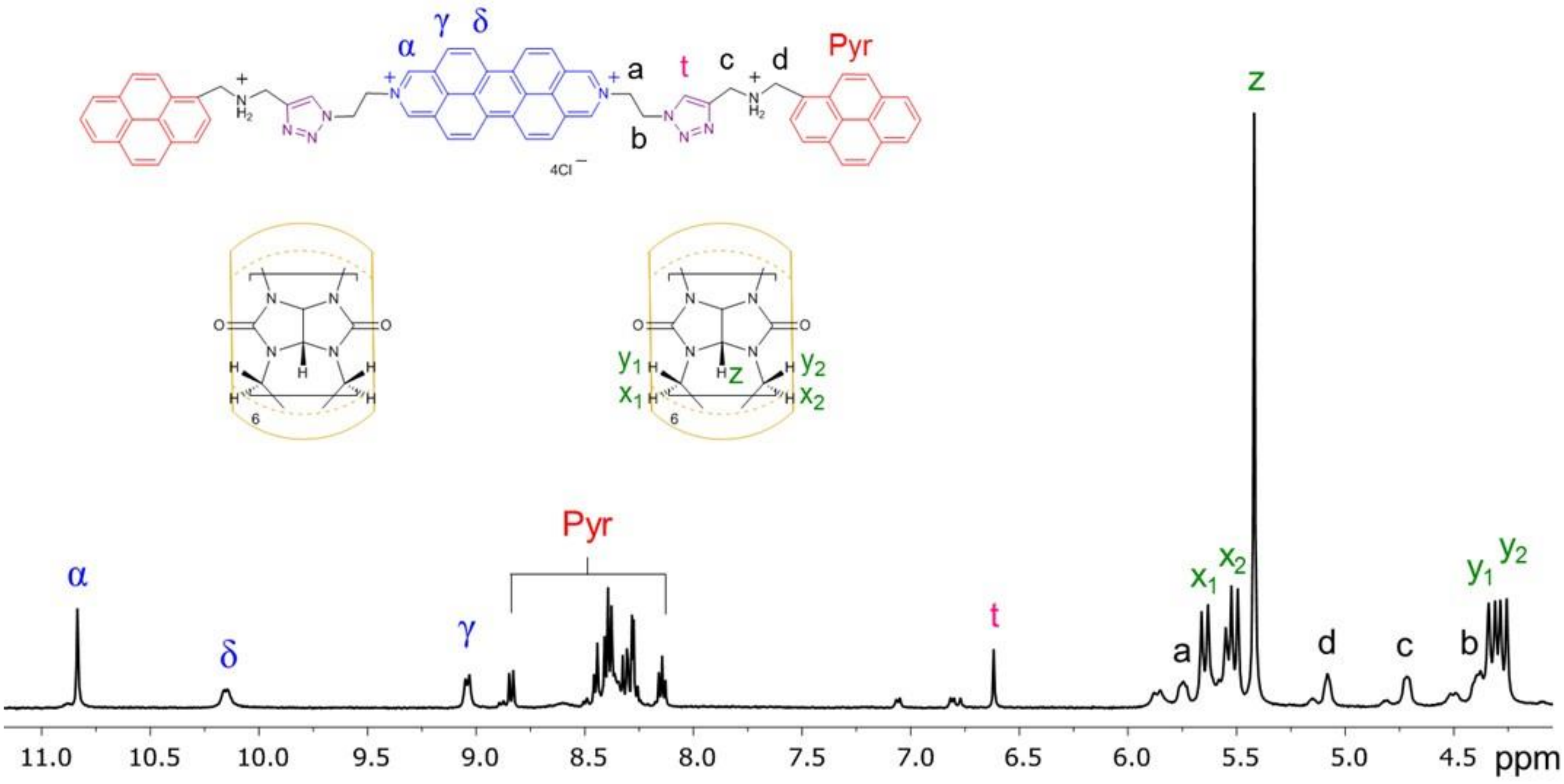

Supplementary Figure 11. ^{1}H NMR spectrum (500 MHz, $(CD_3)_2SO$, 25 °C) of **R3•**$4PF_6$. Rather than illustrate the structural formula of **R3•**$4PF_6$, its components with labeled protons are shown above. On account of the strong aggregation of **R3•**$4PF_6$ in common solvents, the sample concentration was reduced to 125 µM.

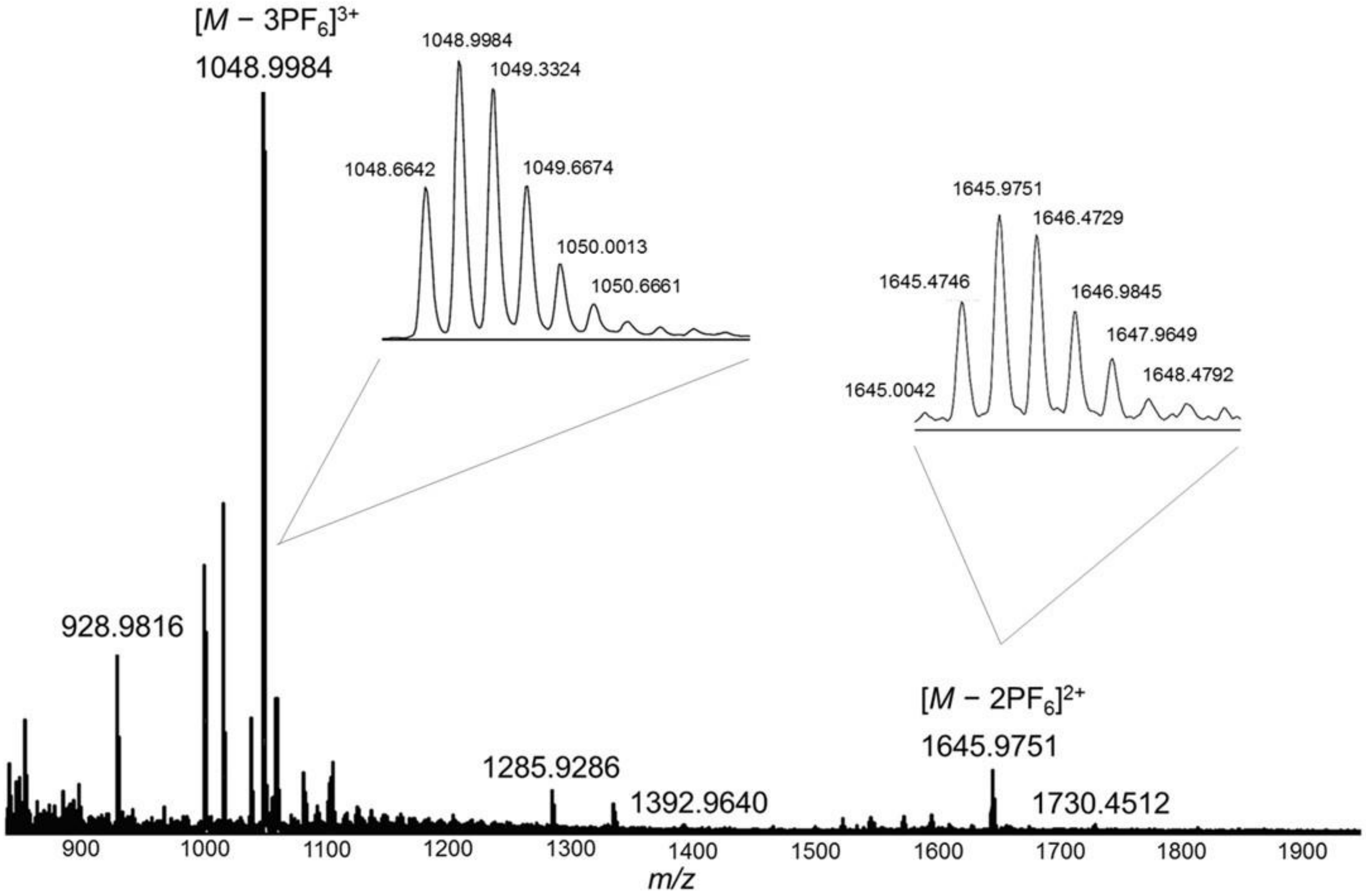

Supplementary Figure 12. High resolution ESI mass spectrum of **R3**•$4PF_6$

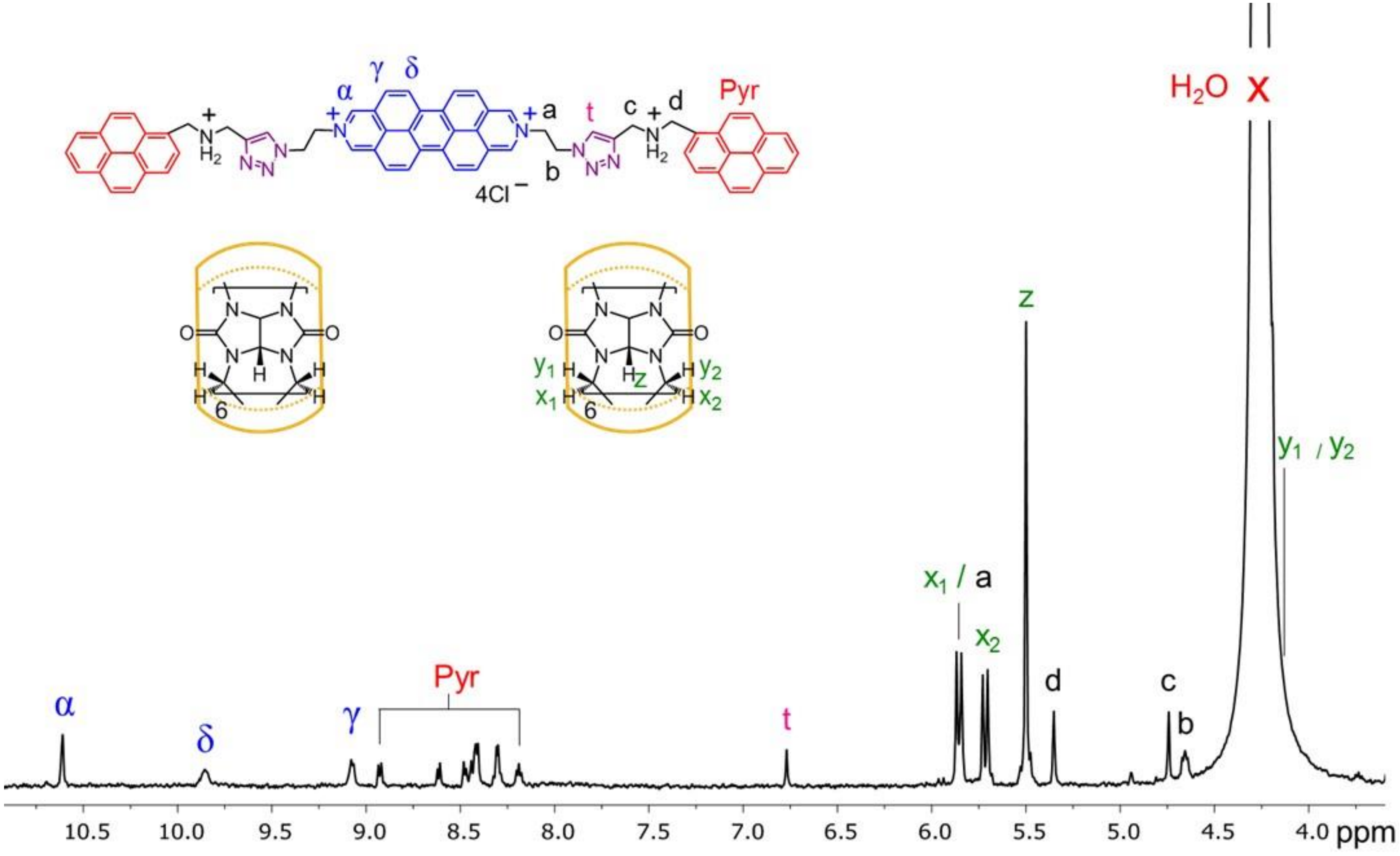

Supplementary Figure 13. ^{1}H NMR spectrum (600 MHz, D_2O, 70 °C) of **R3•**4Cl. Rather than illustrate the structural formula of **R3•**4Cl, its components with labeled protons are shown above.

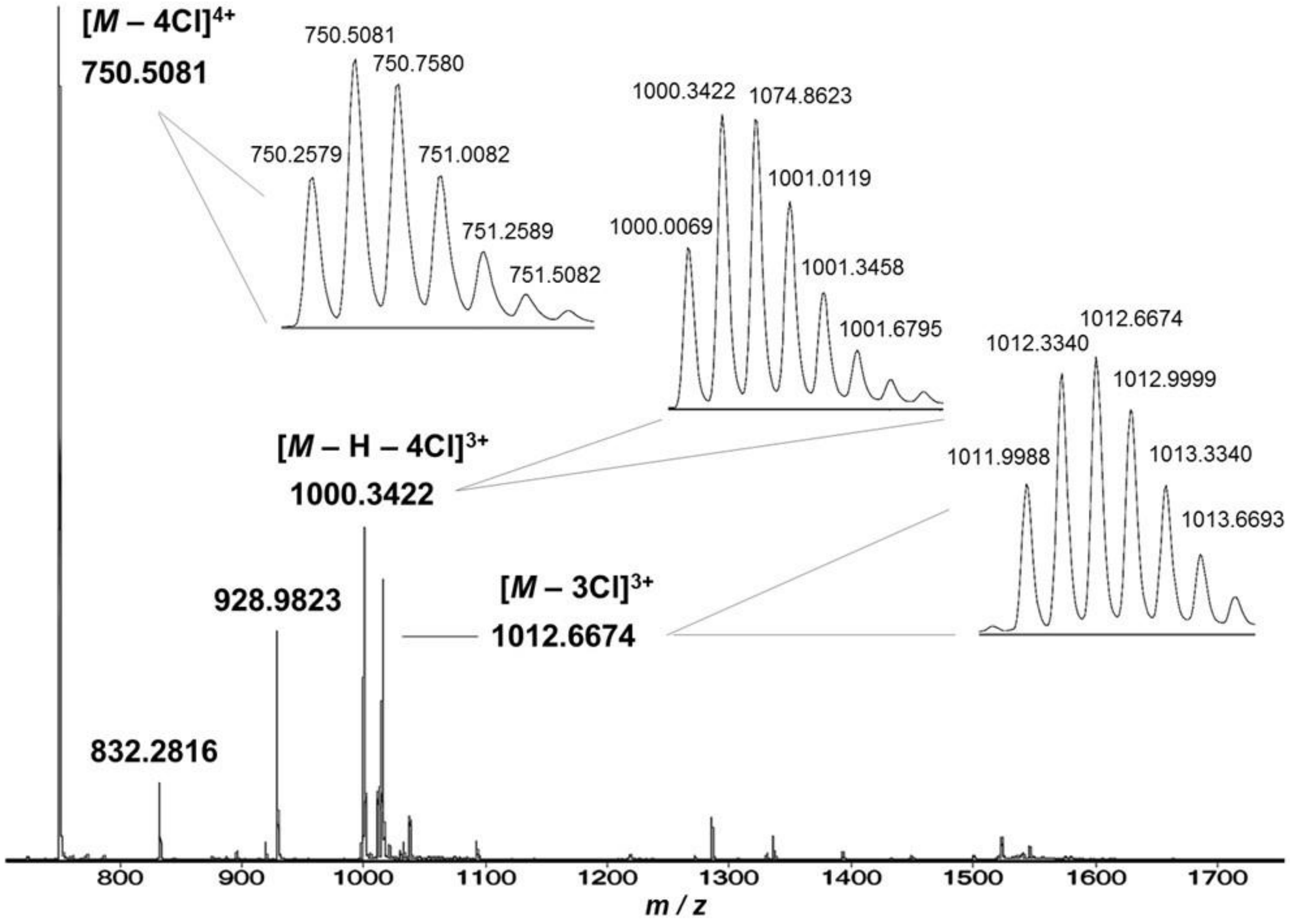

Supplementary Figure 14. High resolution ESI mass spectrum of **R3•**4Cl

Binding Studies between γ-CD and **2**•2Cl

*^{1}H NMR Binding Studies of **2•2Cl** with γ-CD*

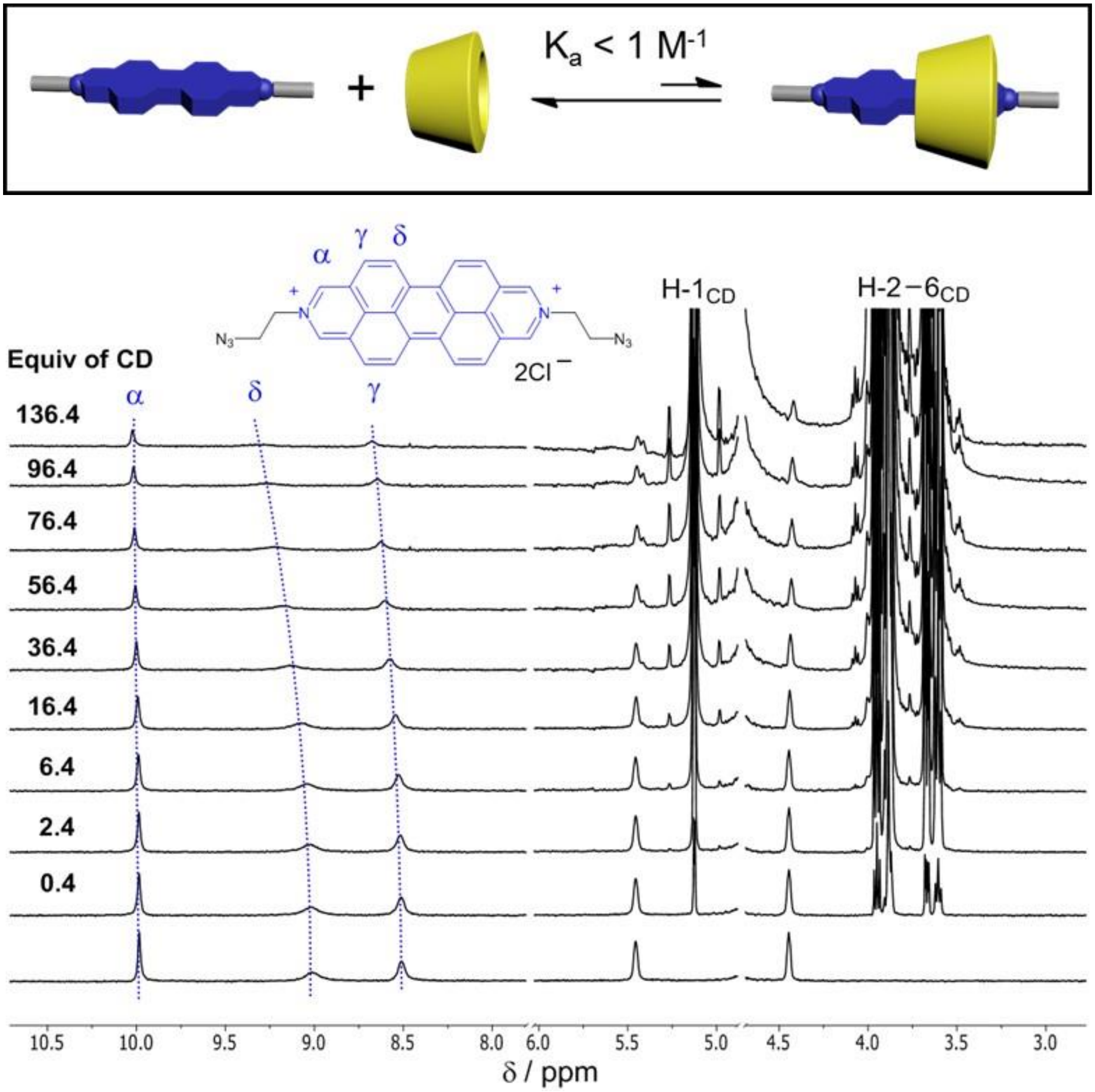

Supplementary Figure 15. Graphical representation (top) of the binding behavior of **2**•2Cl with γ-CD and the ^{1}H NMR spectra (bottom, 600 MHz, 298 K) of **2**•2Cl (initial concentration = 0.5 mM) with increasing amounts of γ-CD (0.2 mM – 28.8 mM) in D_2O.

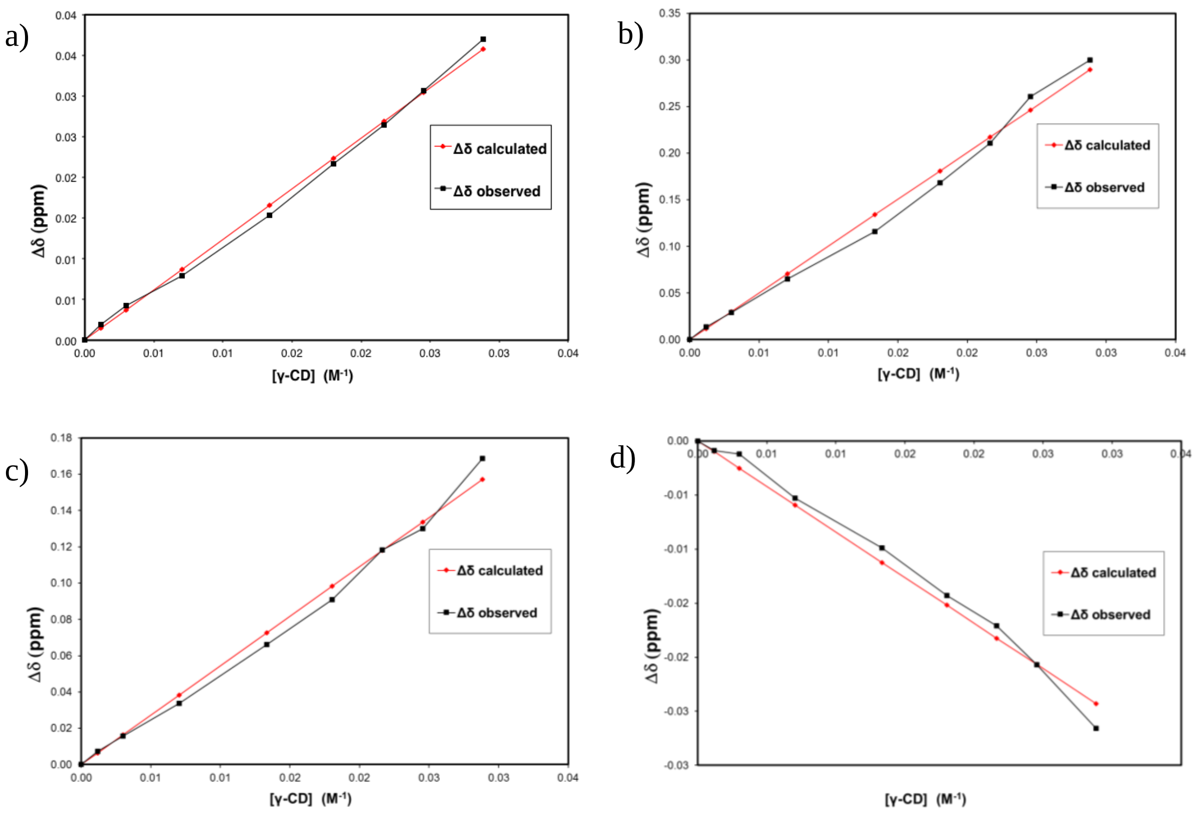

Supplementary Figure 16. Experimental and calculated Δδ values for the following protons a) α, b) δ, c) γ and d) one of the methylene protons from an ^{1}H NMR binding study of **2**•2Cl with γ-CD in D_2O. $K_a < 1$ M^{-1}.

*Isothermal Titration Calorimetry (ITC) Stuides on **2•2Cl** with γ-CD*

Sample Preparation: **2**•2Cl (1.8 mg) was dissolved in H_2O (10 mL) and an excess of CB6 (14 mg) was added to the solution. The mixture was placed in an ultrasonic bath for 10 min and then passed through a syringe filter (0.25 μm). The generated bright yellow solution of the complex **2**•2Cl⊂CB6 was then used for ITC.

The heat (dQ/dt) generated during titrations by the complexation between **2**•2Cl⊂CB[6] and γ-CD is too weak to be measured accurately by ITC. This result suggests that the binding constant (K_a) of **2**•2Cl⊂CB6 and γ-CD is below the lower limit of the ITC measurement ($K_a <$ 50 M^{-1})

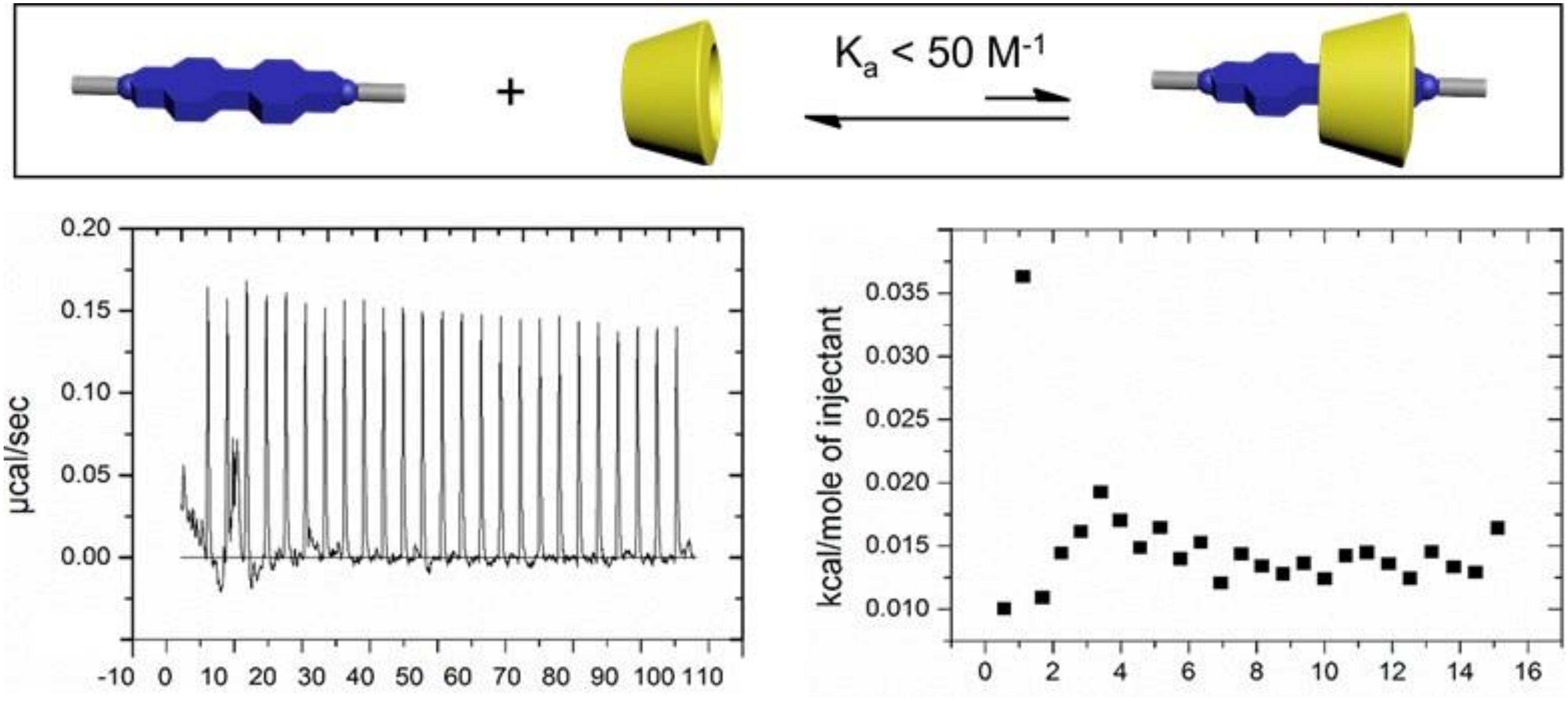

Supplementary Figure 17. Graphical representation (top) of host-guest binding behavior of **2•**2Cl with γ-CD. ITC profiles (bottom) of **2•**2Cl⊂CB6 titrated with γ-CD.

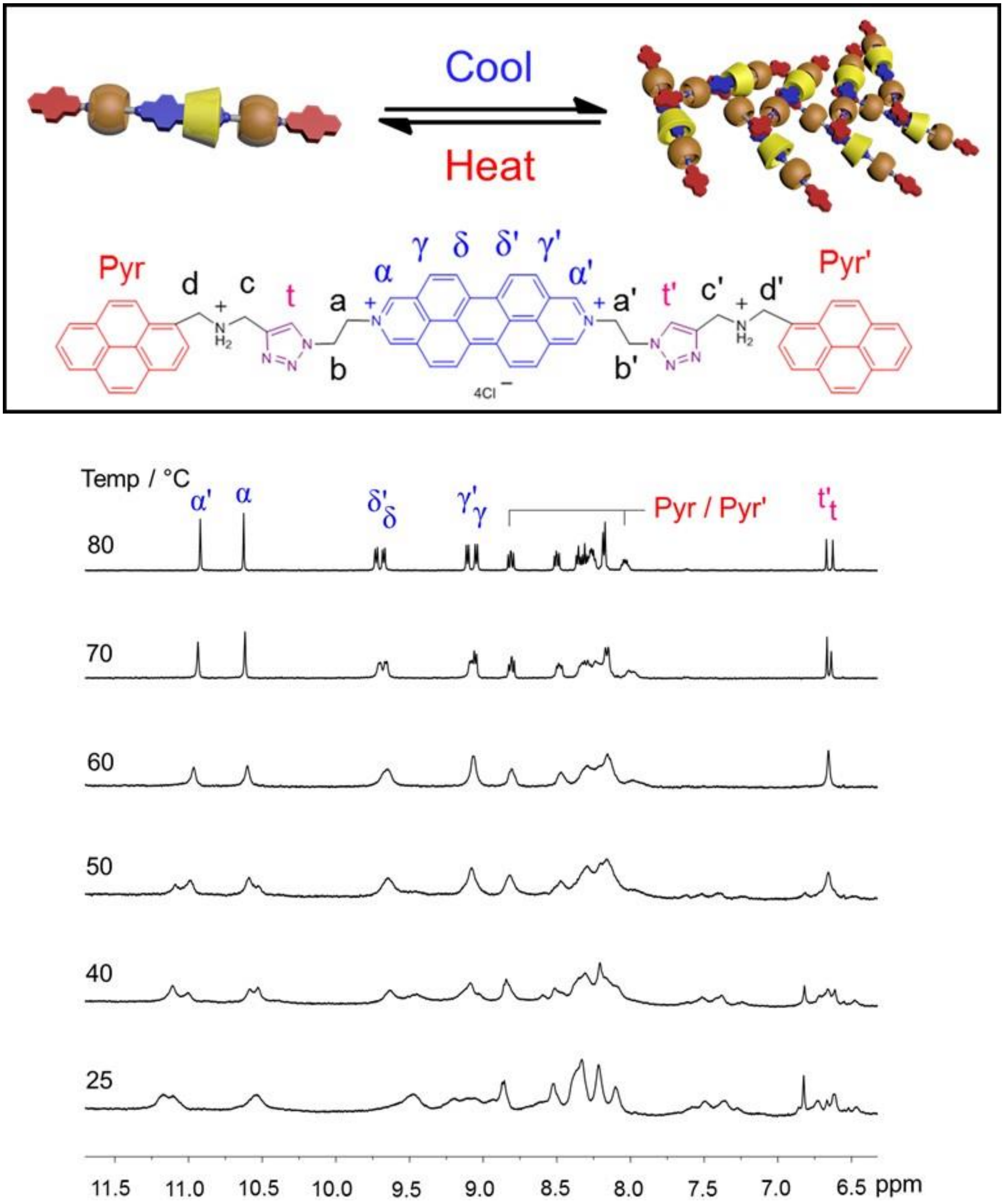

Supplementary Figure 18. Graphical representation (top) of the aggregation of the [4]rotaxane **R4**•4Cl monomers in response to changes in temperature. Temperature-dependent partial ^{1}H NMR spectra (bottom, 600 MHz, D_2O, 500 μM) of **R4**•4Cl.

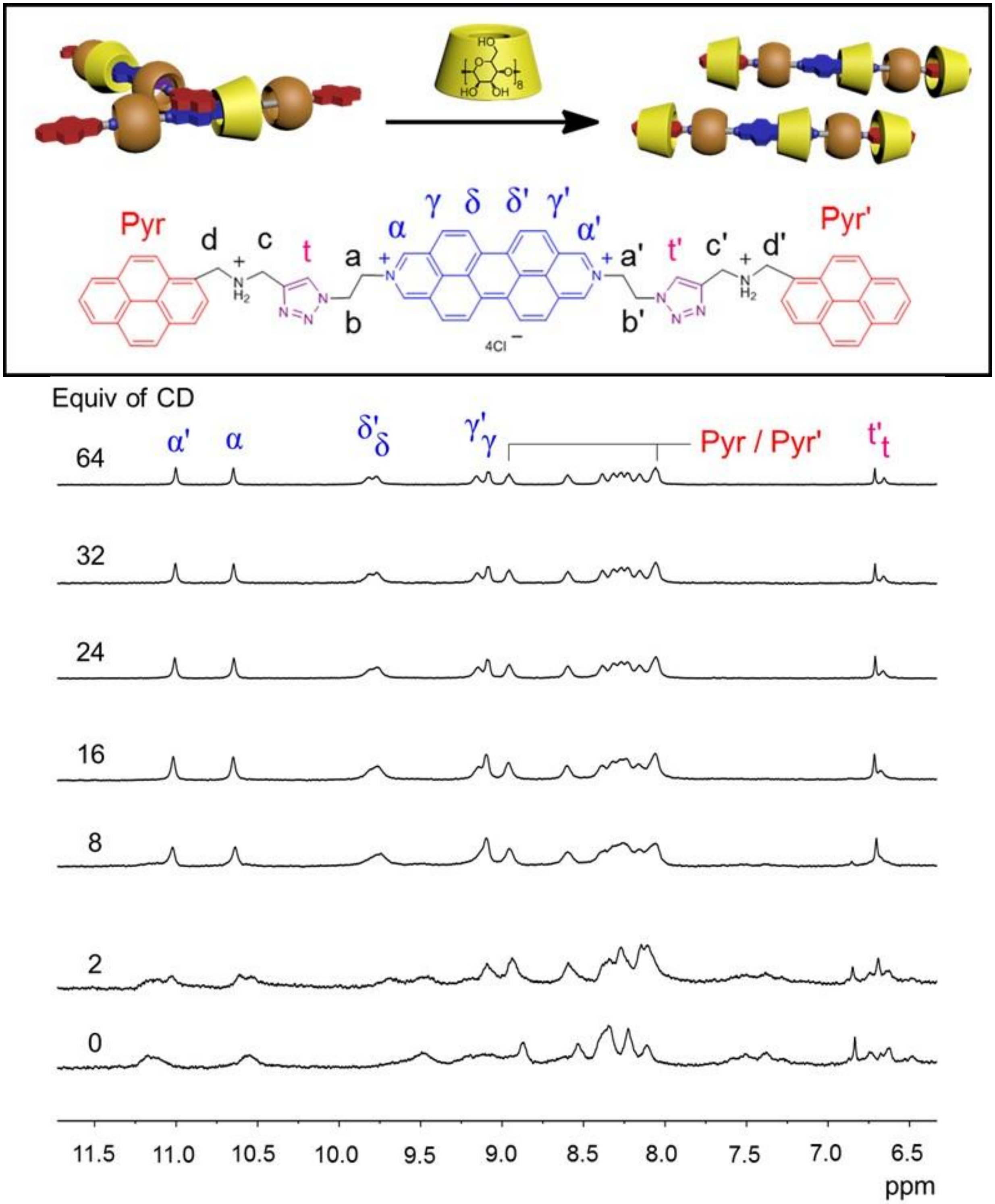

Supplementary Figure 19. Graphical representation (top) of the aggregation of [4]rotaxane **R4**•4Cl in the solid state and its de-aggregation upon the addition of an excess of γ-CD. Partial ^{1}H NMR spectra (bottom, 600 MHz, D_2O, 500 μM) of **R4**•4Cl with increasing amount of γ-CD.

Conformational Analysis of [4]Rotaxane **R4**•4Cl

In the 2D NOESY NMR spectrum (Supplementary Figure 21, bottom), a nuclear Overhauser effect (NOE) correlation peak attributed to proton resonances δ/δ' on the diazoperopyrenium (DAPP) unit and H-6_{CD} on the γ-CD was observed, suggesting that the primary side of the γ-CD torus is pointing towards the center of the DAPP unit. A group of NOE correlation peaks attributed to proton resonances α' and H-3_{CD}, x_1 and H-3_{CD}, y_1 and H-3_{CD}, and z_1 and H-3_{CD} were also observed, indicating that the secondary face of the γ-CD torus forms hydrogen bonding networks with its neighboring CB6 ring. No correlation peaks were observed between the proton resonances on the primary face (H-5_{CD}, H-6_{CD}, and OH-6_{CD}). These results suggest that in the [4]rotaxane **R4**•4PF_6, the γ-CD ring has a fixed co-conformation as shown in Supplementary Figure 21 (top).

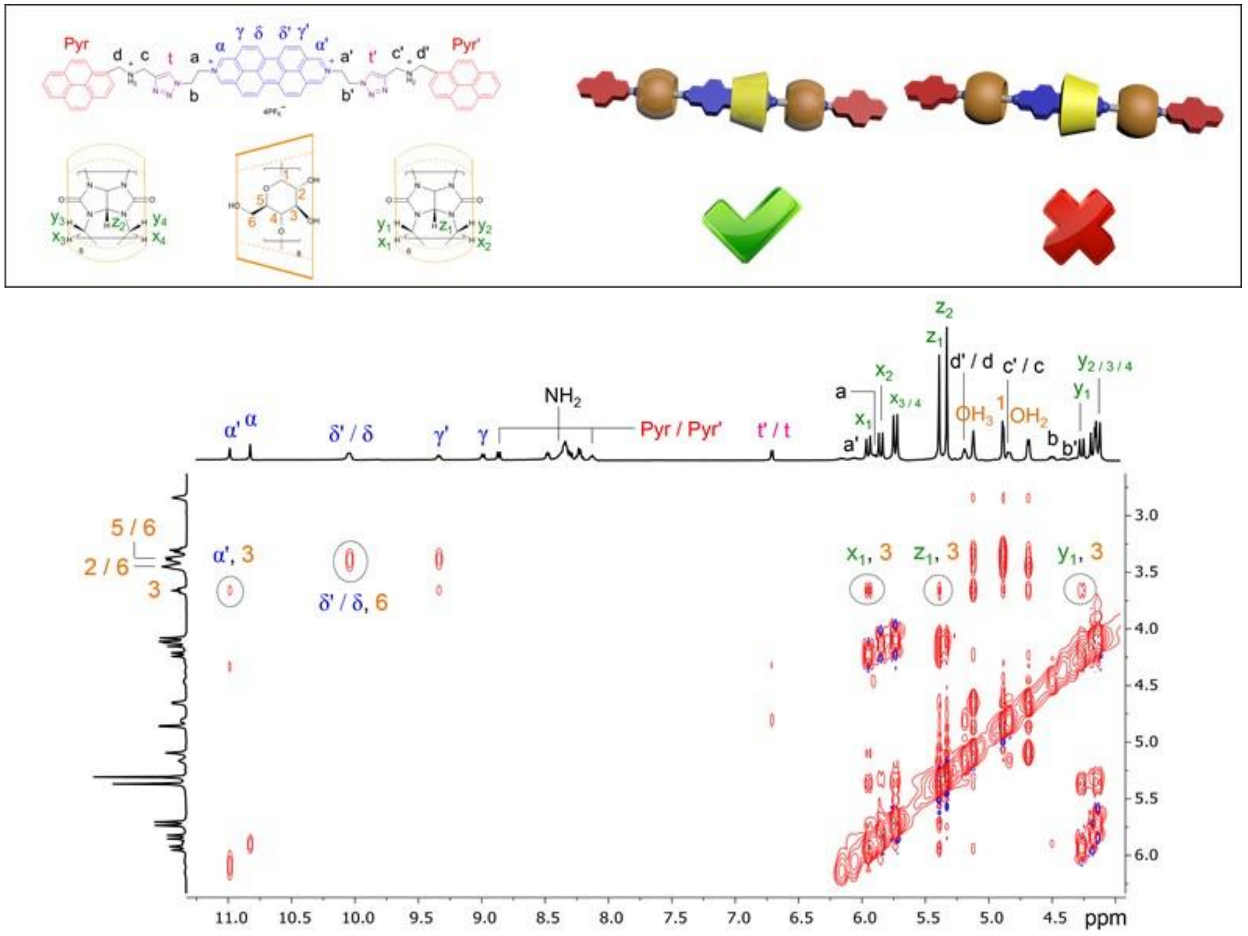

Supplementary Figure 20. Structural formula of the components of **R4**•4PF_6 and graphical representation (top) of the co-conformation of γ-CD in the [4]rotaxane **R4**•4PF_6 analysed from

the partial 2D NOESY NMR spectrum of **R4**•$4PF_6$ (500 MHz, CD_3CN, 25 °C, mixing time: 300 ms).

Variable Temperature ^{1}H NMR Spectroscopic Studies

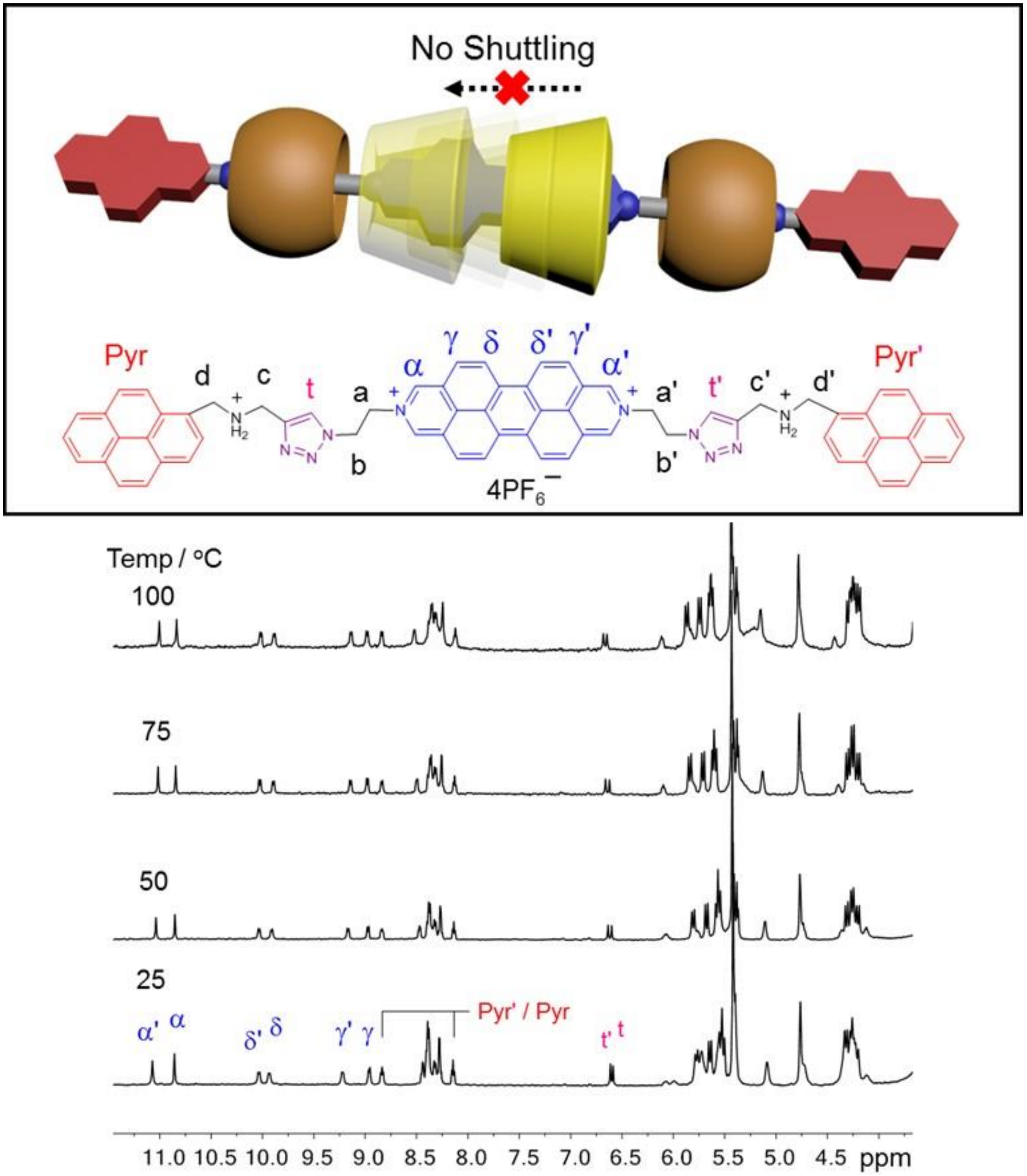

Supplementary Figure 21. Graphical representation (top) of the γ-CD ring in the [4]rotaxane **R4**•4PF_6 which does not shuttle along the dumbbell even at elevated temperature. Temperature-dependent partial ^{1}H NMR spectra (bottom) of [4]rotaxane **R4**•4PF_6 in $(CD_3)_2SO$.

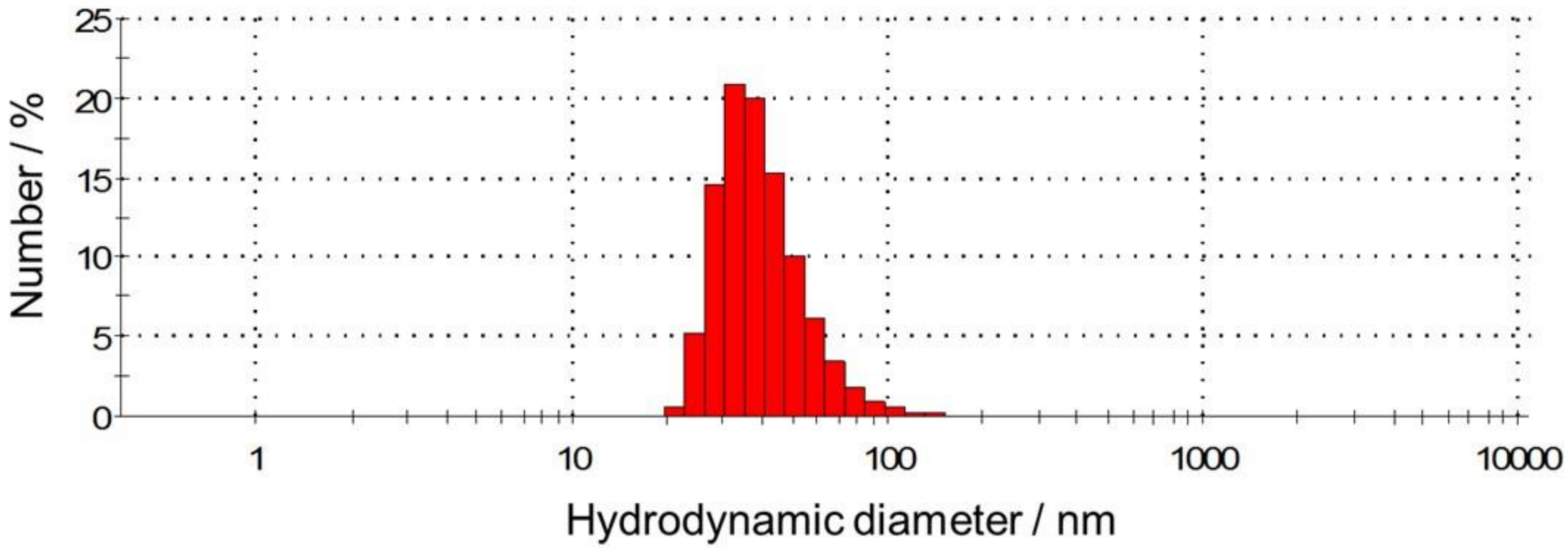

Supplementary Figure 22. Distribution of hydrodynamic diameters of **R4**•4Cl aggregates in H_2O (500 µM, 25 ºC) measured by dynamic light scattering. The observed hydrodynamic diameters of **R4**•4Cl species are broadly distributed between 20 – 120 nm, which is indicative of significant aggregation of **R4**•4Cl in H_2O at 500 µM.

Aggregation Studies of **R4**•4Cl

In an aggregated solution of **R4**•4Cl, where the monomeric species is **R4** and the aggregated oligomers are $\mathbf{R4}_n$, then,

$$n\mathrm{R4} \underset{}{\overset{K}{\rightleftharpoons}} \mathrm{R4}_n \quad (1)$$

in which

$$\mathrm{R4} \overset{K_{a1}}{\rightleftharpoons} \mathrm{R4}_2 \overset{K_{a2}}{\rightleftharpoons} \mathrm{R4}_3 \overset{K_{a3}}{\rightleftharpoons} \mathrm{R4}_4 \cdots \overset{K_{a(n-1)}}{\rightleftharpoons} \mathrm{R4}_n \quad (2)$$

When the aggregation process is homogenous and non-cooperative during the nucleation process, then

$$K_{a1} = K_{a2} = K_{a3} = \cdots = K_{a(n-1)} \quad (3)$$

Then total aggregation process is equivalent to the dimerization process

$$2\mathrm{R4} \overset{4K_{agg}}{\rightleftharpoons} \mathrm{R4}_2 \quad (4)$$

Thus,

$$K_{agg} = \frac{[\mathrm{R4}_2]}{4[\mathrm{R4}]^2} \quad (5)$$

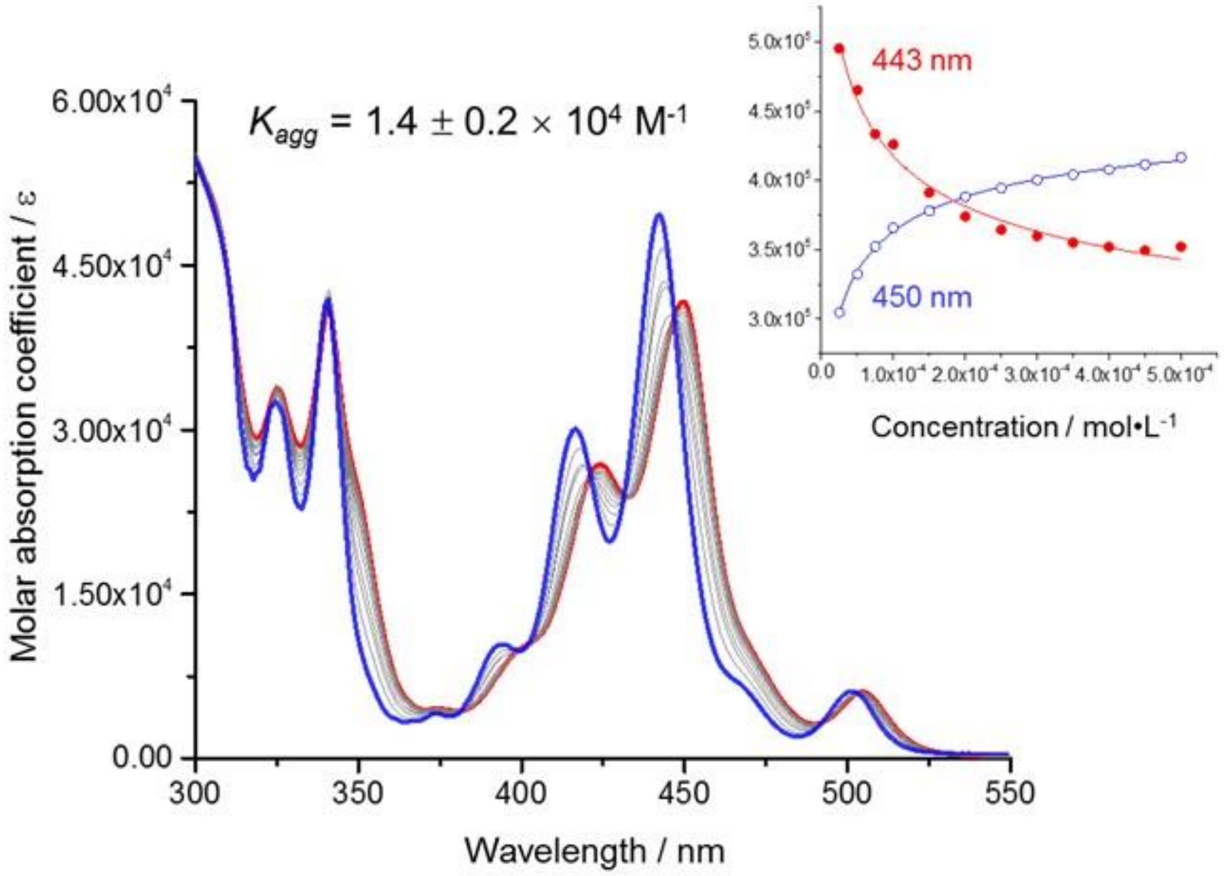

Supplementary Figure 23. Concentration dependent (0.025 mM – 0.5 mM) UV/Vis absorption spectra of aqueous solutions of **R4**•4Cl measured at 25 °C. Inset, fitted curves based on the absorption data at 443 and 450 nm, respectively. The aggregation constant K_{agg} of was **R4**•4Cl in aqueous solution was calculated to be $K_{agg} = 1.4 \pm 0.2 \times 10^4$ M^{-1}.

The data were well fitted using the dimerization model (adjusted $R^2 > 0.98$) as we proposed, indicating that the aggregation of **R4**•4Cl in aqueous solution is non-cooperative and the dimerization model is the appropriate model to describe the aggregation process.

Solution Phase Photophysical Studies

UV-Vis Spectroscopy

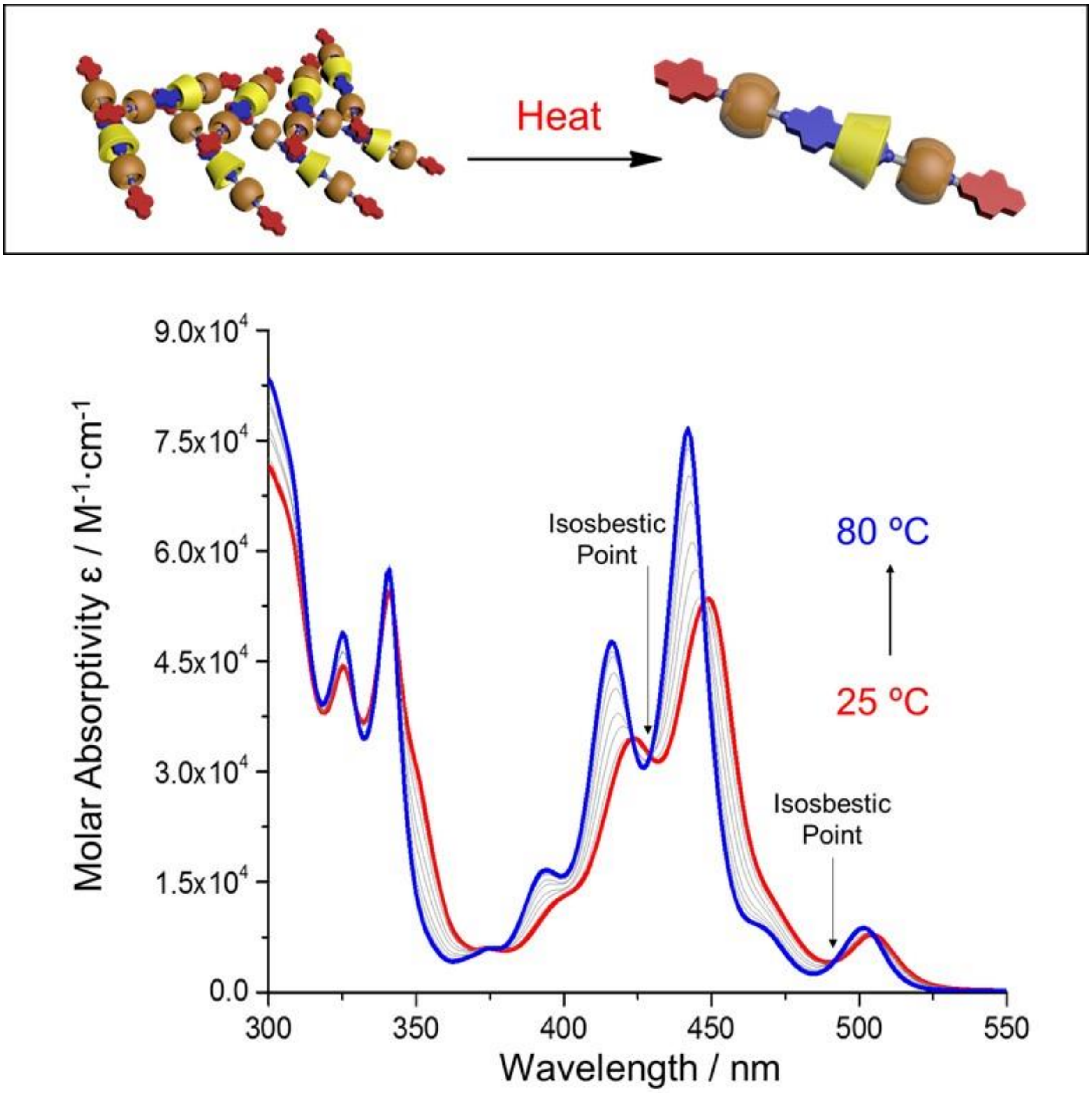

Supplementary Figure 24. Graphical representation (top) of the aggregation the [4]rotaxane **R4•**4Cl monomers in response to changes in temperature. Temperature dependent UV-Vis spectra (bottom) of the hetero[4]rotaxane **R4•**4Cl at 300 μM in water.

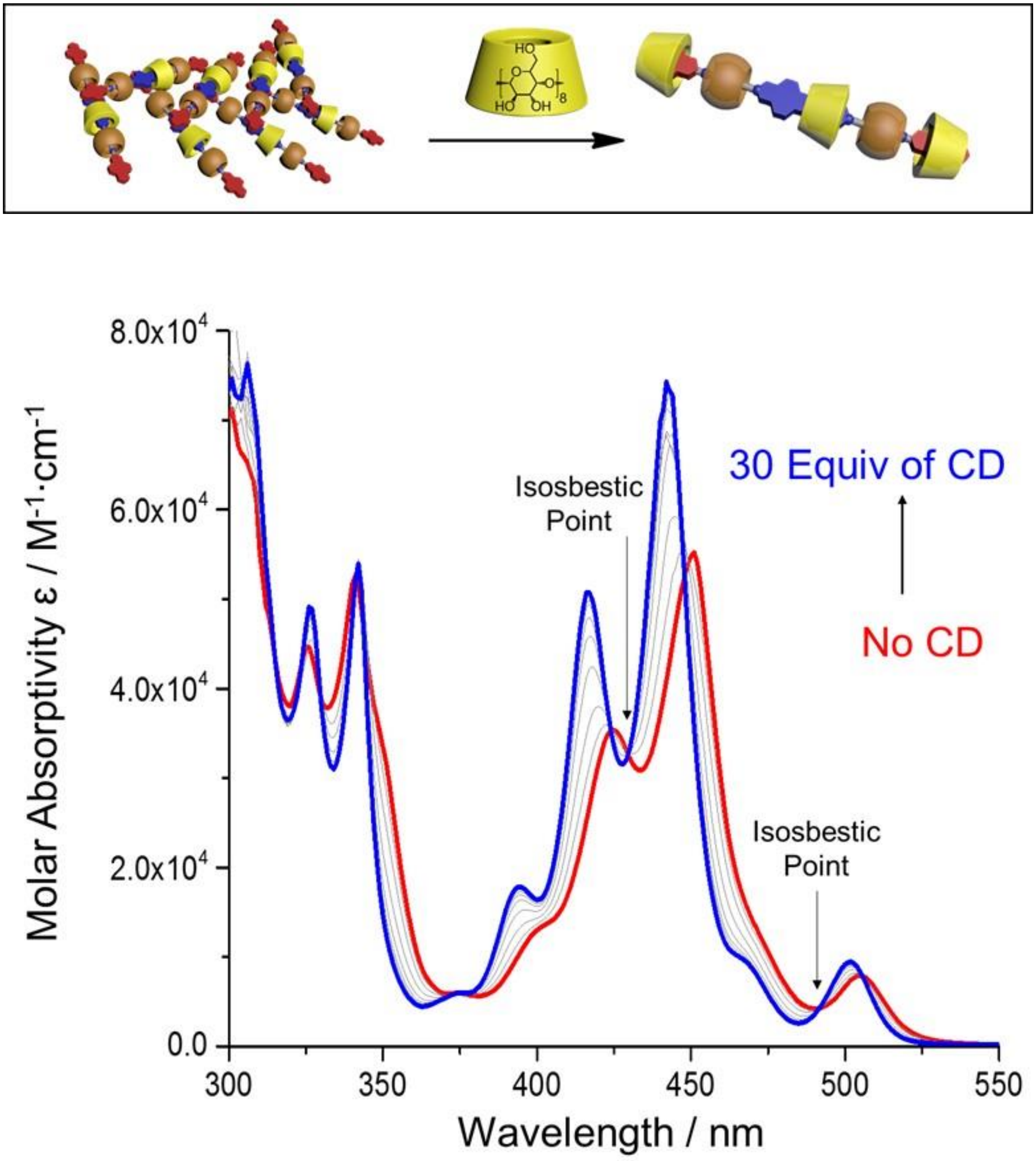

Supplementary Figure 25. Graphical representation (top) of the deaggregation the [4]rotaxane **R4**•4Cl in response to addition of γ-CD. UV-Vis spectra (bottom) of up to 30 equiv of γ-CD titrated in a 500 μM aqueous solution the hetero[4]rotaxane **R4**•4Cl.

Steady-State Fluorescence Spectroscopy

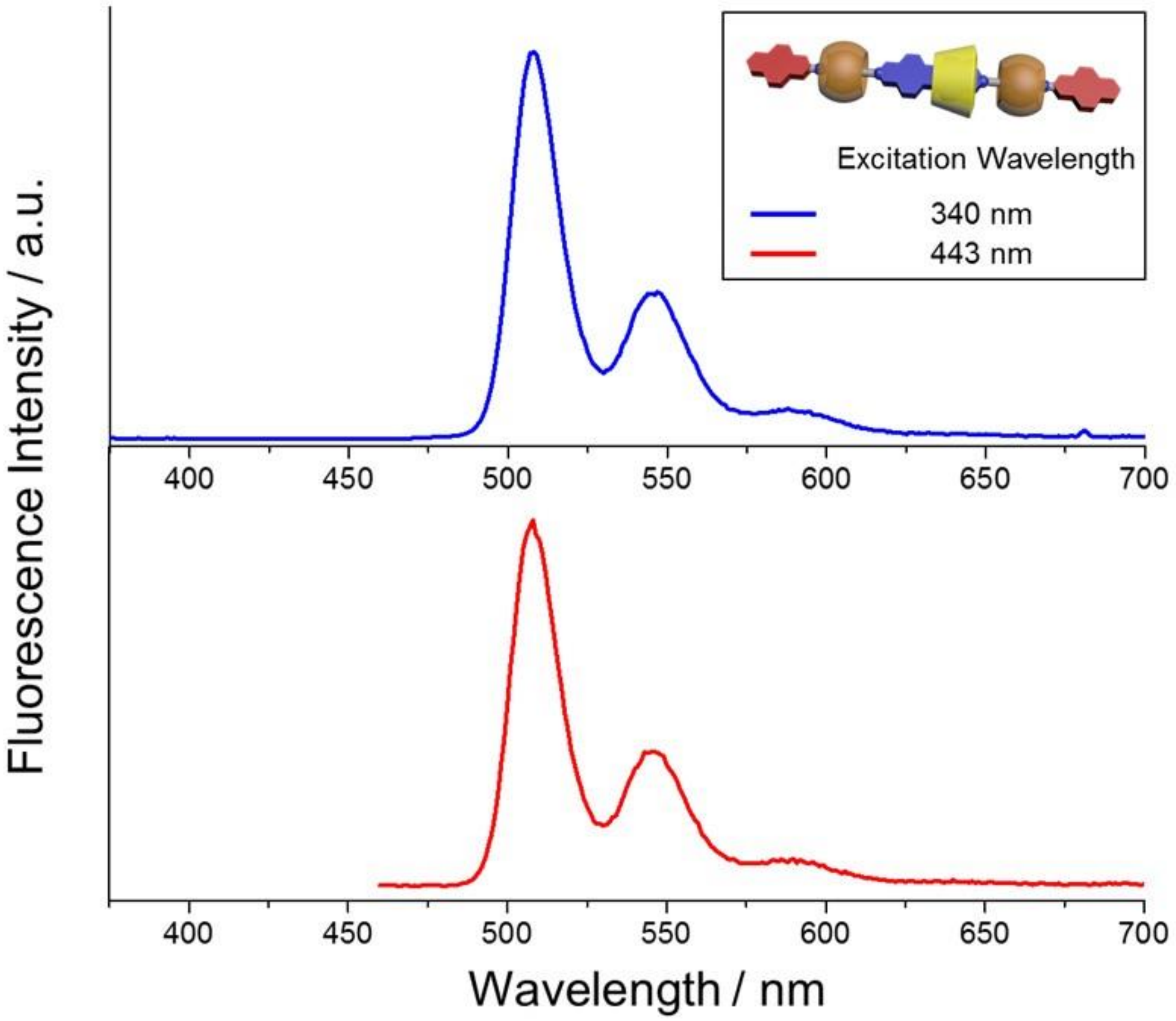

Supplementary Figure 26. Fluorescence emission spectra, which were excited at 340 nm (blue) and 443 nm (red), respectively, of a 5 μM aqueous solution of hetero[4]rotaxane **R4**•4Cl measured at 25 ^{o}C.

Quantum Yield Measurements

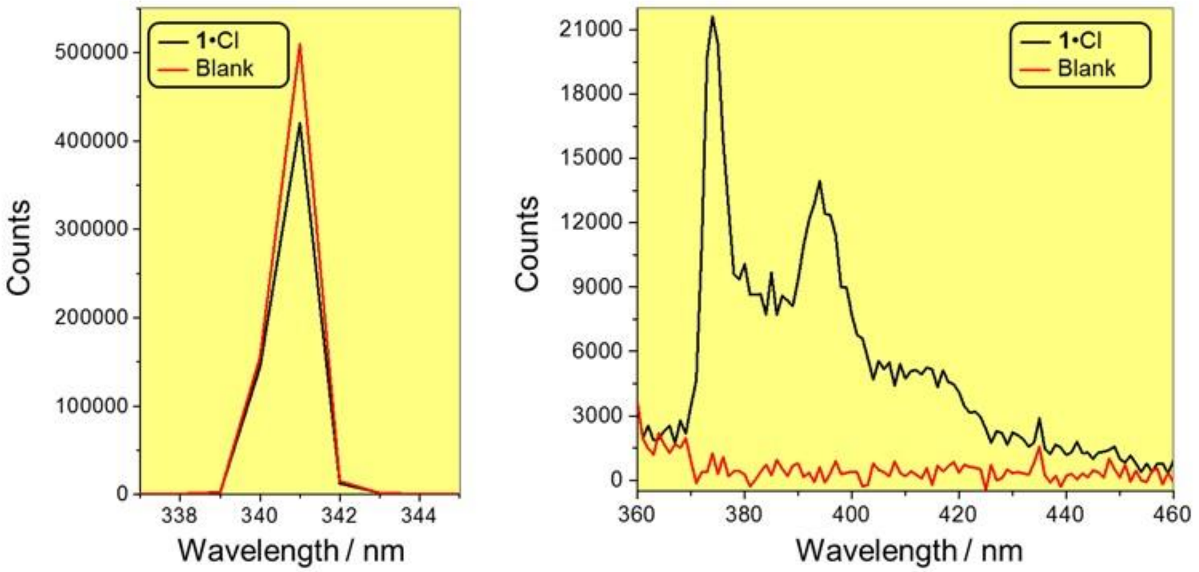

Supplementary Figure 27. Traces from quantum yield measurement for a solution of **1**•Cl (5 μM) in water at 298 K, using water as a blank. Excitation wavelength = 341 nm. The absolute quantum yield of **1**•Cl is 22.8 ± 0.25 %.

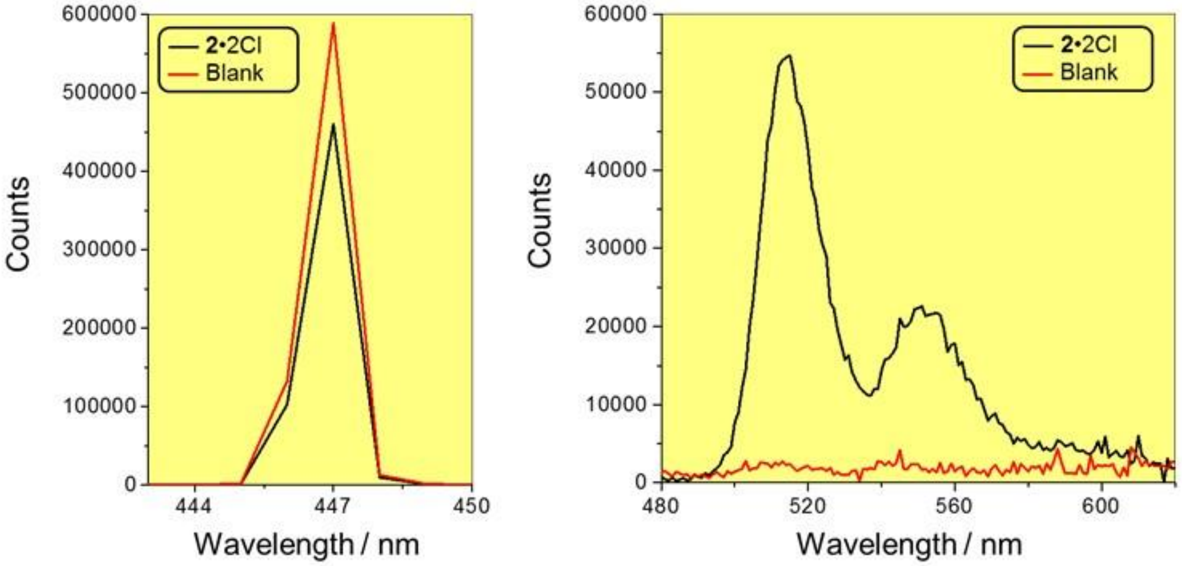

Supplementary Figure 28. Traces from quantum yield measurement for a solution of **2**•2Cl (5 μM) in water at 298 K, using water as a blank. Excitation wavelength = 447 nm. The absolute quantum yield of **2**•2Cl is 51.3 ± 0.36 %.

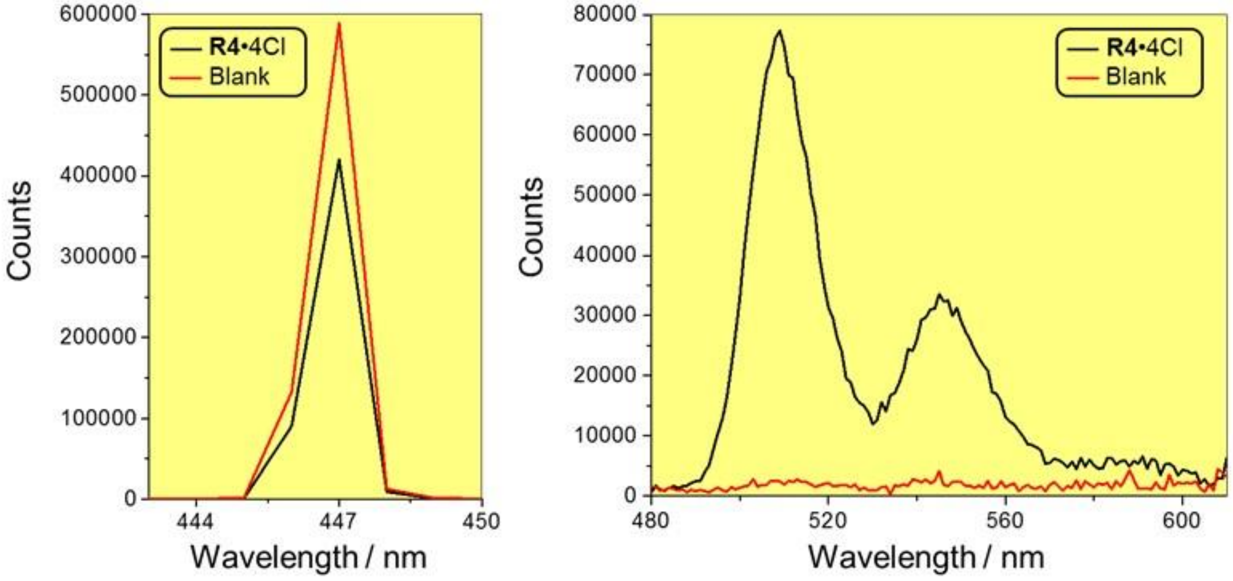

Supplementary Figure 29. Traces from quantum yield measurement for a solution of **R4**•4Cl (5 μM) in water at 298 K, using water as a blank. Excitation wavelength = 447 nm. The absolute quantum yield of **R4**•4Cl is 52.4 ± 0.27 %.

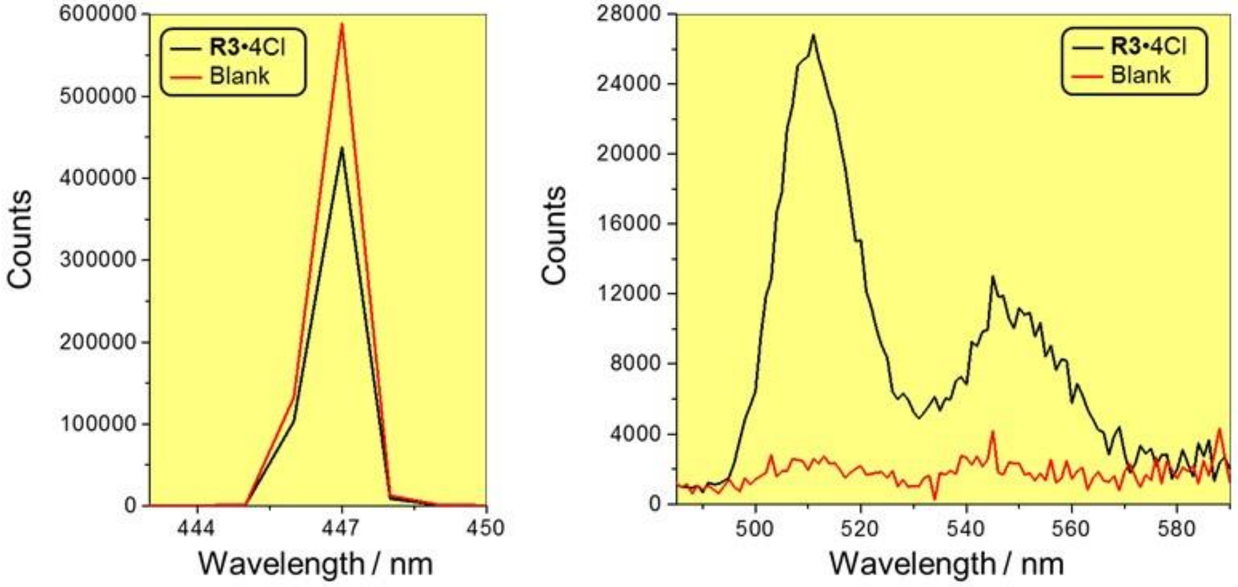

Supplementary Figure 30. Traces from quantum yield measurement for a solution of **R3**•4Cl (5 μM) in water at 298 K, using water as a blank. Excitation wavelength = 447 nm. The absolute quantum yield of **R3**•4Cl is 18.3 ± 0.11%.

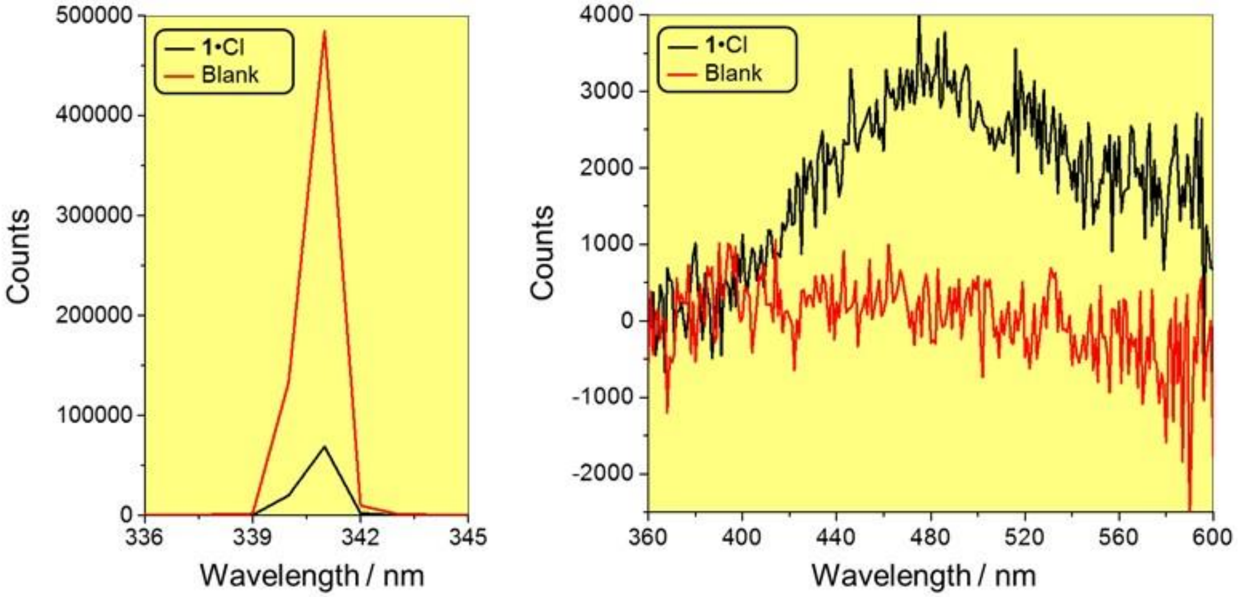

Supplementary Figure 31. Traces from quantum yield measurement for a freeze-dried solid sample of **1**•Cl, using $BaSO_4$ powder as a blank. Excitation wavelength = 341 nm. The absolute quantum yield of **1**•Cl is 4.0 ± 0.01 %.

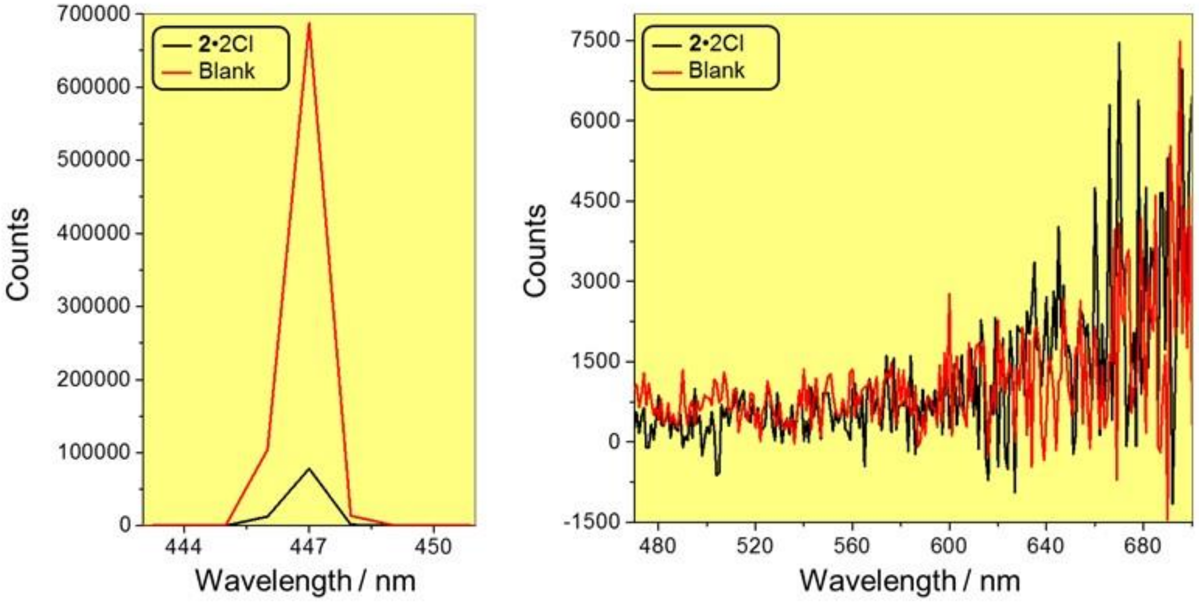

Supplementary Figure 32. Traces from quantum yield measurement for a freeze-dried solid sample of **2**•2Cl, using $BaSO_4$ powder as a blank. Excitation wavelength = 447 nm. The absolute quantum yield of **2**•2Cl is 0.1 %.

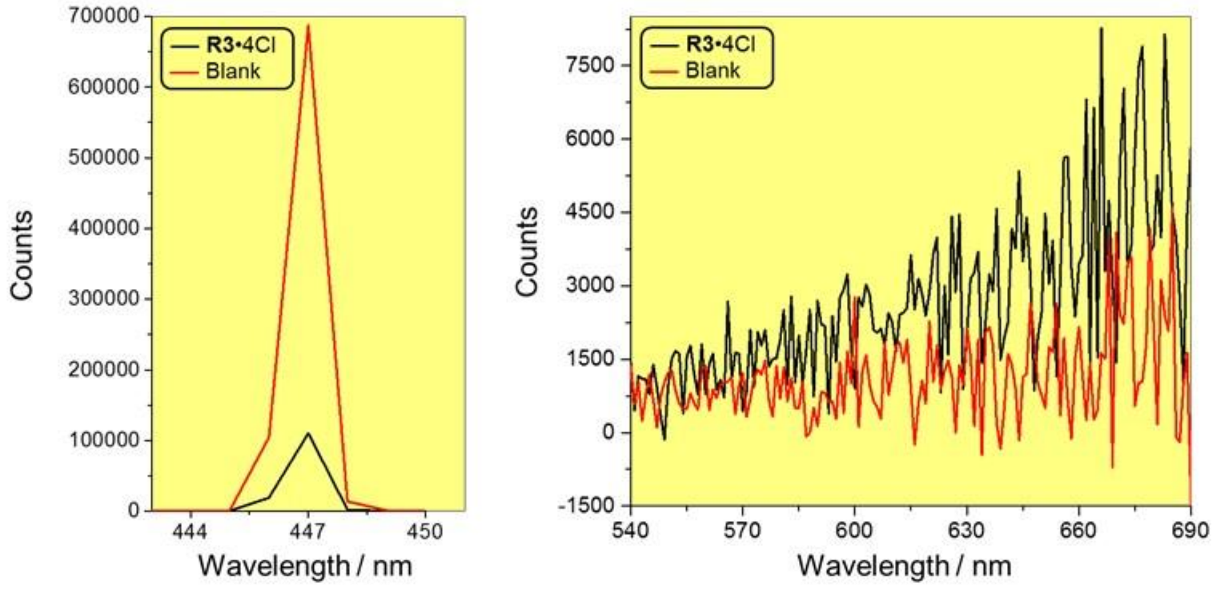

Supplementary Figure 33. Traces from quantum yield measurement for a freeze-dried solid sample of **R3**•4Cl, using $BaSO_4$ powder as a blank. Excitation wavelength = 447 nm. The absolute quantum yield of **R3**•4Cl is 1.7 ± 0.01 %.

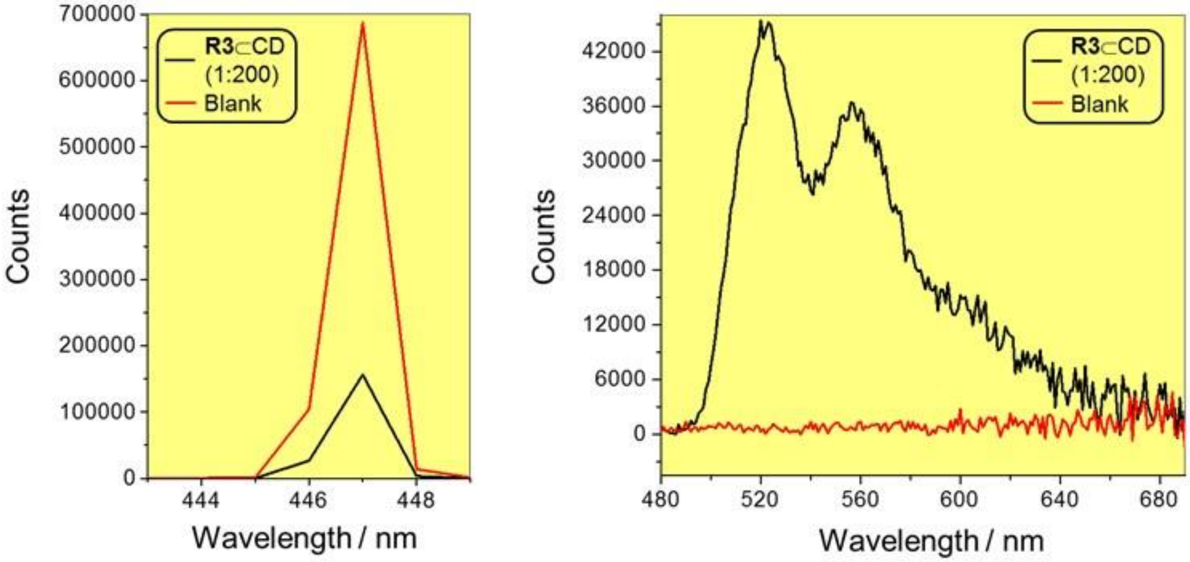

Supplementary Figure 34. Traces from quantum yield measurement on a freeze-dried solid sample of **R3**⊂γ-CD, with a molar ratio **R3**•4Cl:γ-CD = 1:200. $BaSO_4$ powder was used as a blank. Excitation wavelength = 447 nm. The absolute quantum yield of **R3**⊂CD (1:200) is 25.9 ± 0.04 %.

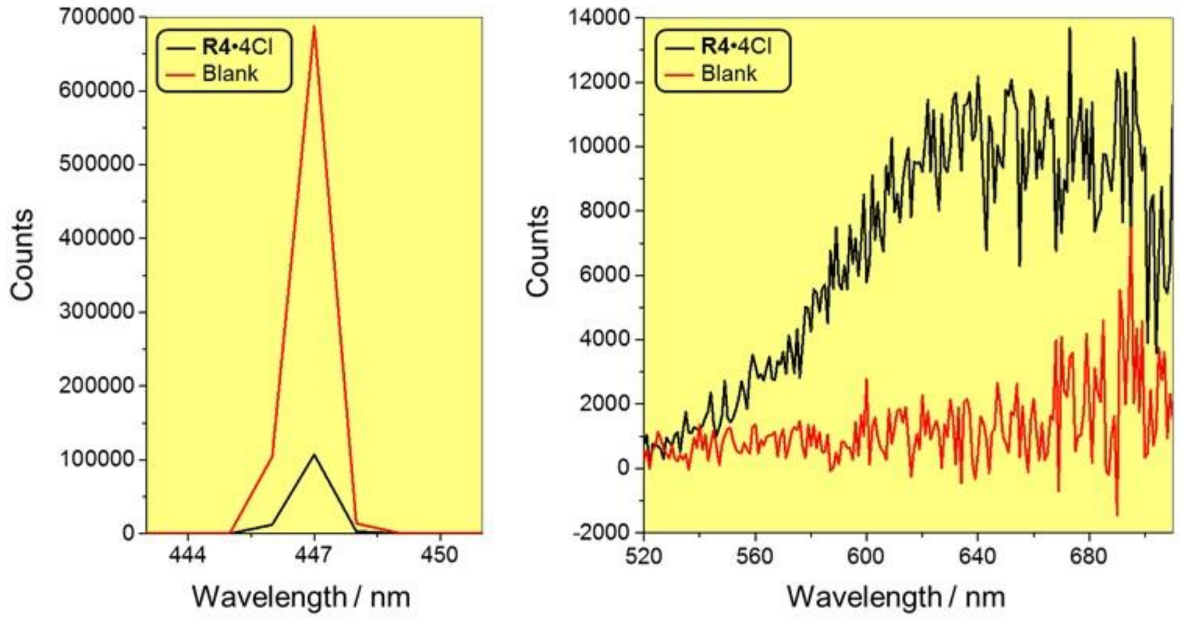

Supplementary Figure 35. Traces from quantum yield measurement for a freeze-dried solid sample of **R4**•4Cl, using $BaSO_4$ powder as a blank. Excitation wavelength = 447 nm. The absolute quantum yield of **R4**•4Cl is 7.7 ± 0.01 %.

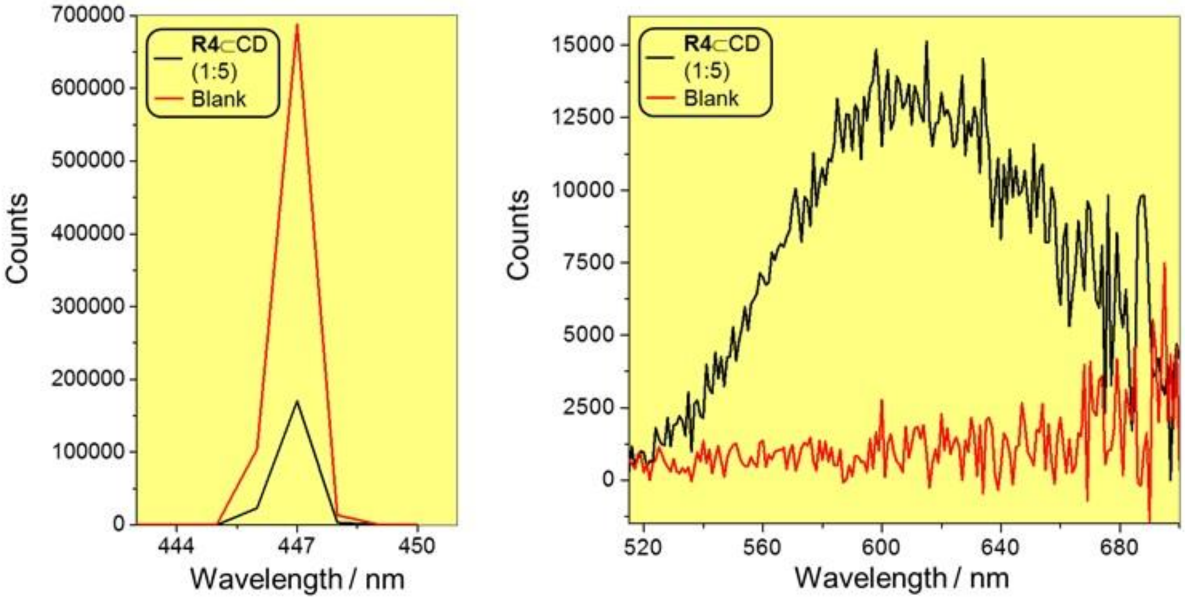

Supplementary Figure 36. Traces from quantum yield measurement on a freeze-dried solid sample of **R4**⊂γ-CD, with a molar ratio **R4**•4Cl:γ-CD = 1:5. $BaSO_4$ powder was used as a blank. Excitation wavelength = 447 nm. The absolute quantum yield of **R4**⊂CD (1:5) is 10.4 ± 0.02 %.

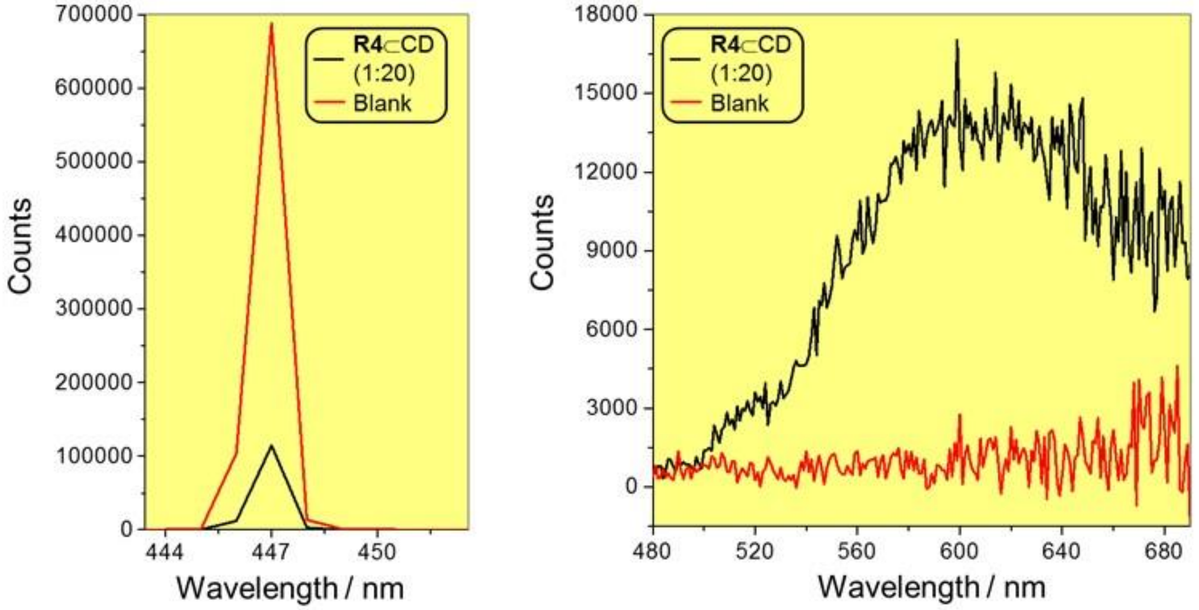

Supplementary Figure 37. Traces from quantum yield measurement for a freeze-dried sample of **R4**⊂γ-CD, with a molar ratio **R4**•4Cl:γ-CD = 1:20. $BaSO_4$ powder was used as a blank. Excitation wavelength = 447 nm. The absolute quantum yield of **R4**⊂CD (1:20) is 12.1 ± 0.02 %.

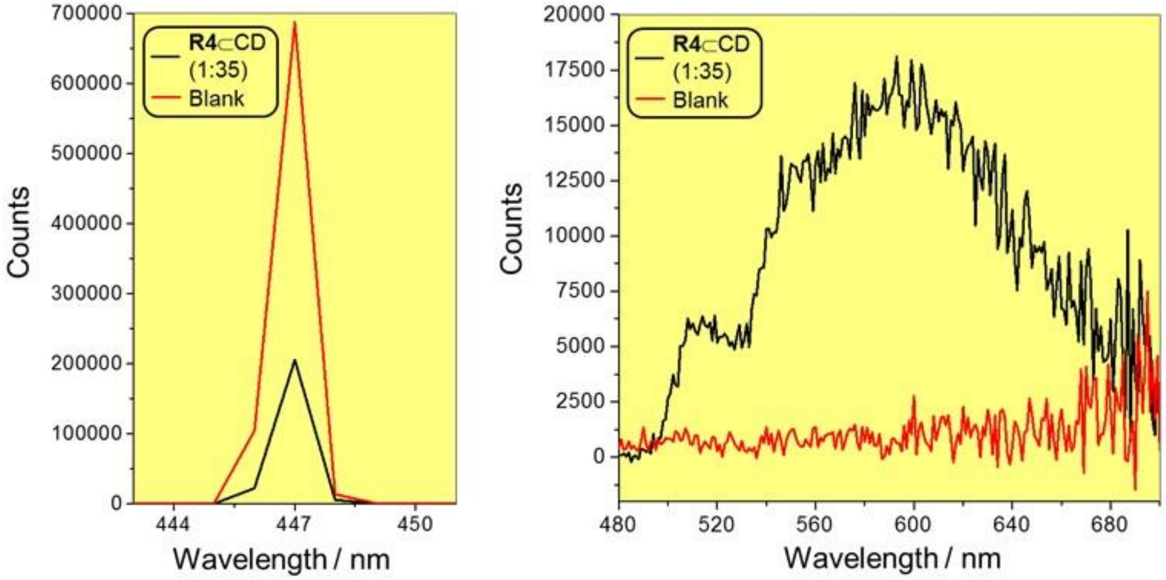

Supplementary Figure 38. Traces from quantum yield measurement for a freeze-dried sample of **R4**⊂γ-CD, with a molar ratio **R4**•4Cl:γ-CD = 1:35. $BaSO_4$ powder was used as a blank. Excitation wavelength = 447 nm. The absolute quantum yield of **R4**⊂CD (1:35) is 15.7 ± 0.03 %.

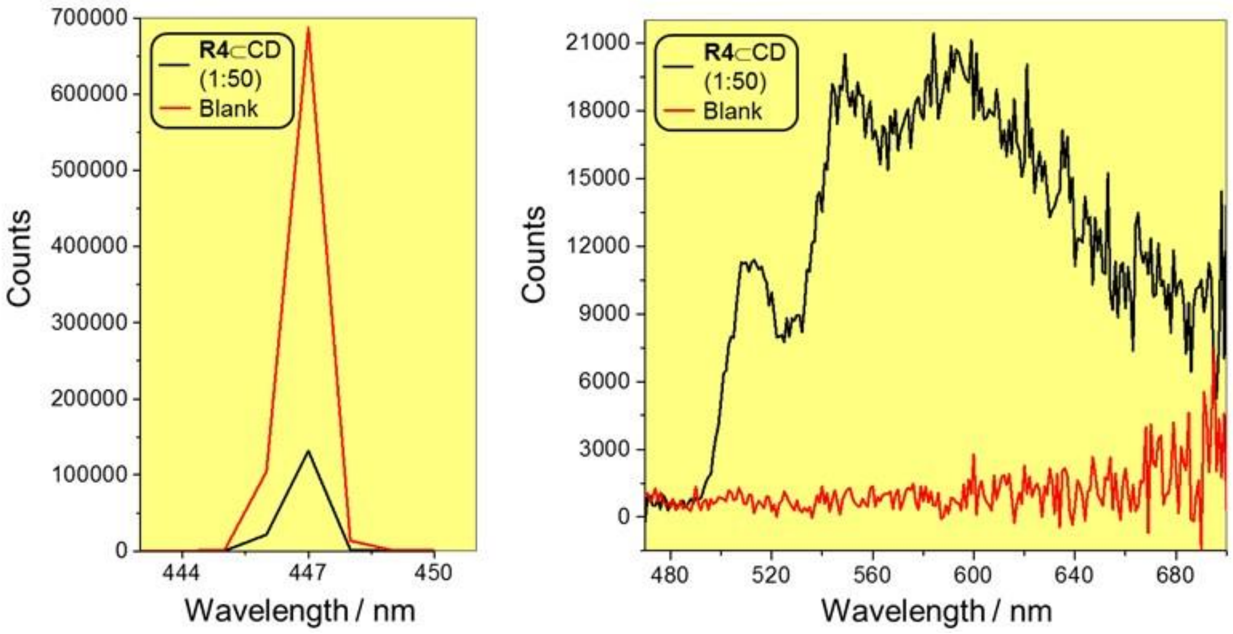

Supplementary Figure 39. Traces from quantum yield measurement on a freeze-dried solid sample of **R4**⊂CD, in which **R4**•4Cl:CD = 1:50 in molar ratio. $BaSO_4$ powder was used as a blank. Excitation wavelength = 447 nm. The absolute quantum yield of **R4**⊂CD (1:50) is 19.6 ± 0.03 %.

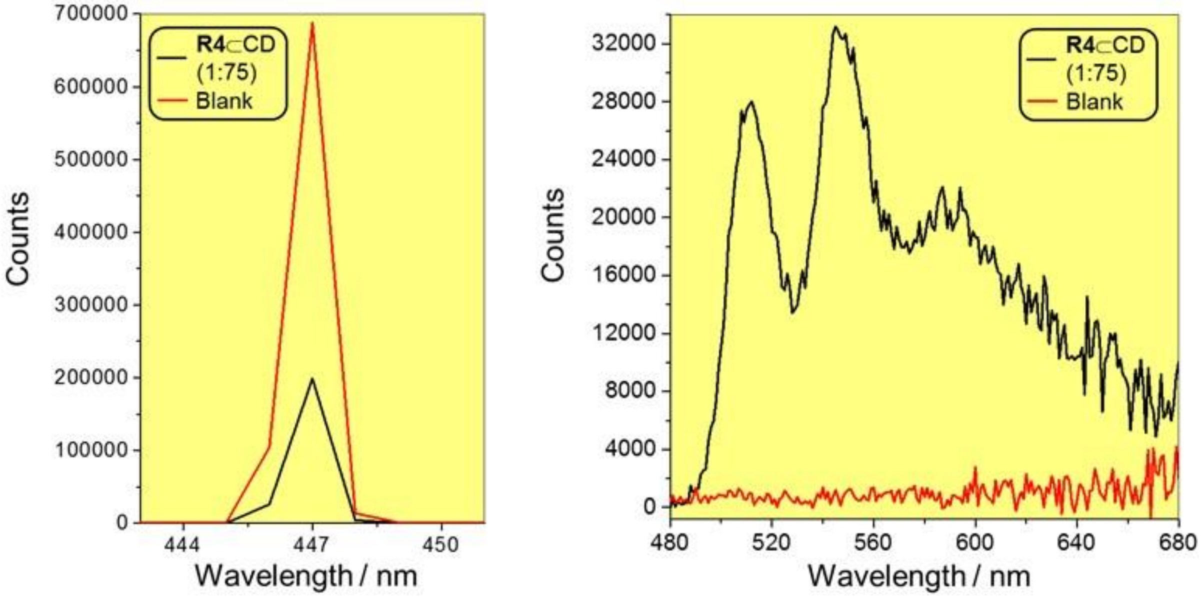

Supplementary Figure 40. Traces from quantum yield measurement for a freeze-dried sample of **R4**⊂γ-CD, with a molar ratio of **R4**•4Cl:γ-CD = 1:75. $BaSO_4$ powder was used as a blank. Excitation wavelength = 447 nm. The absolute quantum yield of **R4**⊂CD (1:75) is 25.8 ± 0.03 %.

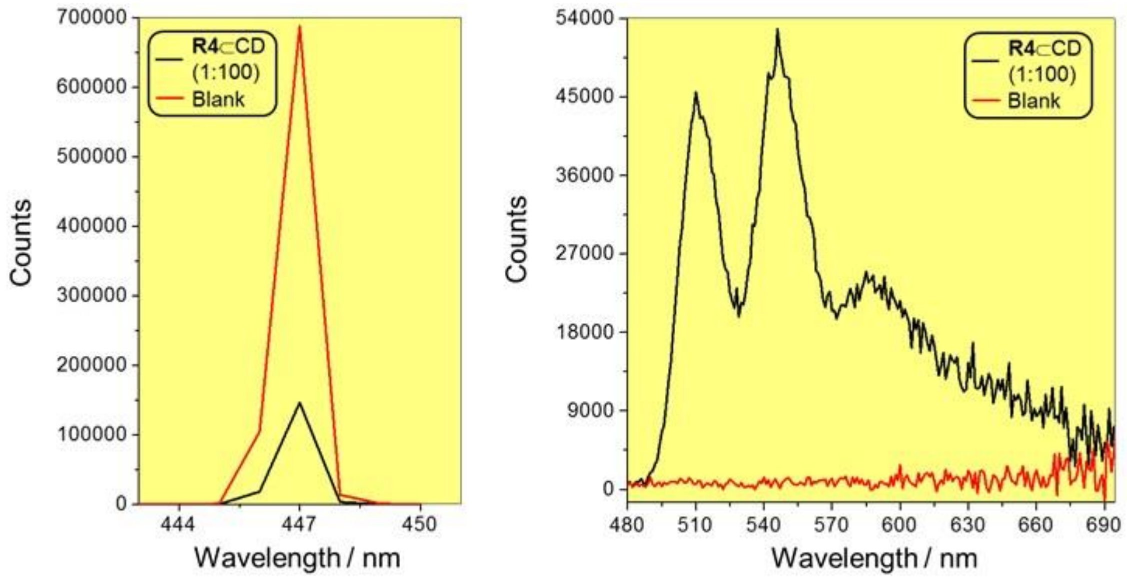

Supplementary Figure 41. Traces from quantum yield measurement for a freeze-dried sample of **R4**⊂γ-CD, with a molar ratio **R4**•4Cl:CD = 1:100. $BaSO_4$ powder was used as a blank. Excitation wavelength = 447 nm. The absolute quantum yield of **R4**⊂CD (1:100) is 31.0 ± 0.05 %.

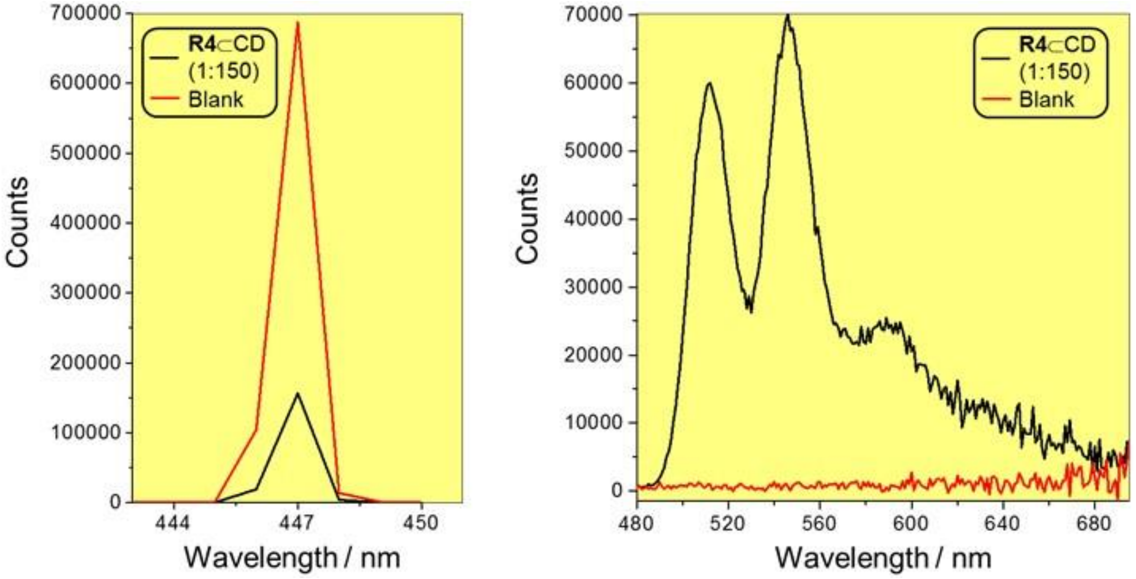

Supplementary Figure 42. Traces from quantum yield measurement for a freeze-dried sample of **R4**⊂γ-CD, with a molar ratio **R4**•4Cl:γ-CD = 1:150. $BaSO_4$ powder was used as a blank. Excitation wavelength = 447 nm. The absolute quantum yield of **R4**⊂CD (1:150) is 36.9 ± 0.06 %.

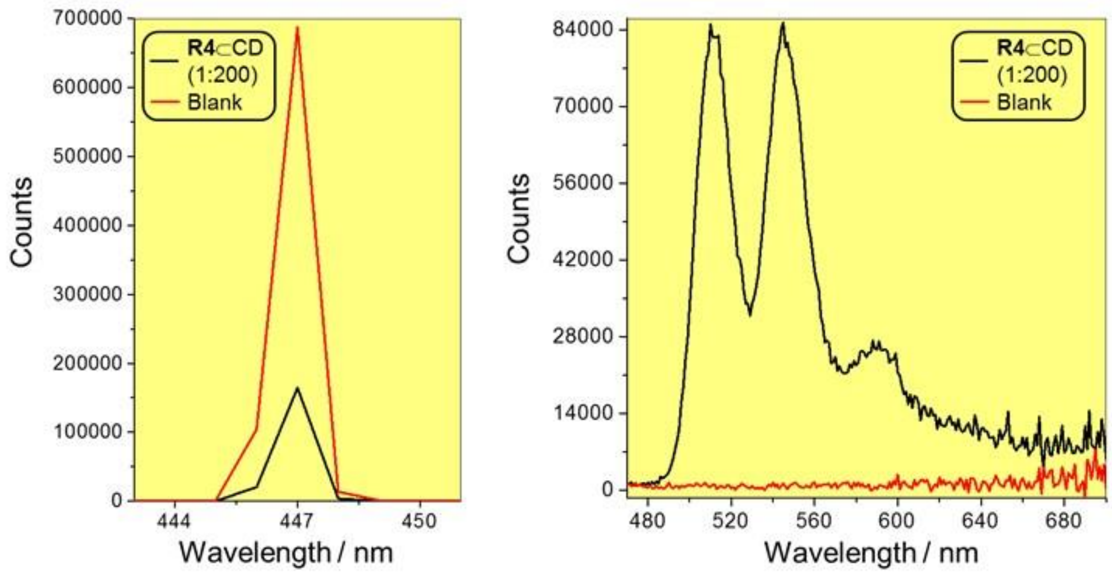

Supplementary Figure 43. Traces from quantum yield measurement on a freeze-dried solid sample of **R4**⊂CD, in which **R4**•4Cl:CD = 1:200 in molar ratio. $BaSO_4$ powder was used as a blank. Excitation wavelength = 447 nm. The absolute quantum yield of **R4**⊂CD (1:200) is 45.7 ± 0.08 %.

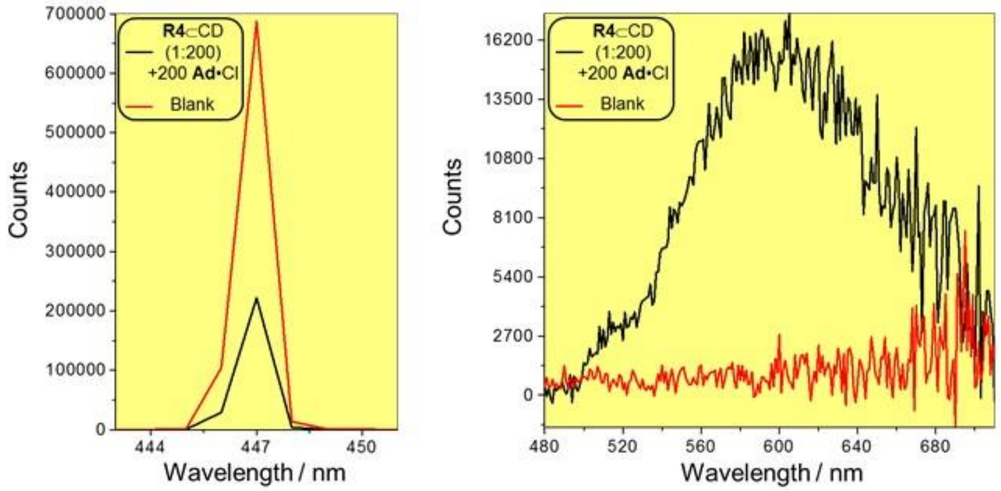

Supplementary Figure 44. Traces from quantum yield measurement on a freeze-dried solid sample of **R4**⊂CD, in which **R4**•4Cl:CD = 1:200 in molar ratio, followed by the addition of 200 equiv of **Ad**•Cl. $BaSO_4$ powder was used as a blank. Excitation wavelength = 447 nm. The absolute quantum yield of **R4**⊂CD (1:200) +200 **Ad**•Cl is 15.4 ± 0.03 %.

Förster Resonance Energy Transfer (FRET) Studies

The emission (Supplementary Figure 45) of the pyrenyl unit of **1**•Cl exhibits a significant overlap with the absorption of the DAPP unit in **2**•2Cl, suggesting that the FRET process could take place if the donor **1**•Cl and the acceptor **2**•2Cl are in close proximity. In the UV-Vis spectrum of an aqueous **R4**•4Cl solution, characteristic absorption bands attributed to the pyrenyl and DAPP units are observed in the 318–365 and 365–520 nm regions. The emission spectrum of **R4**•4Cl is independent of the excitation wavelength, giving (Supplementary Figure 46) identical emission spectra. No pyrenyl unit emission is observed at around 350–450 nm. The excitation spectrum of **R4**•4Cl is similar to its UV-Vis spectrum. These results suggest that energy transfer between the pyrenyl and DAPP units takes place with near quantitative (> 99%) efficiency. The simulated structure of $\mathbf{R4}^{4+}$ shows that the distance between the pyrenyl and

DAPP unit is within 10–12 Å. This distance is in good agreement with a high FRET process according to the literature reports.[1]

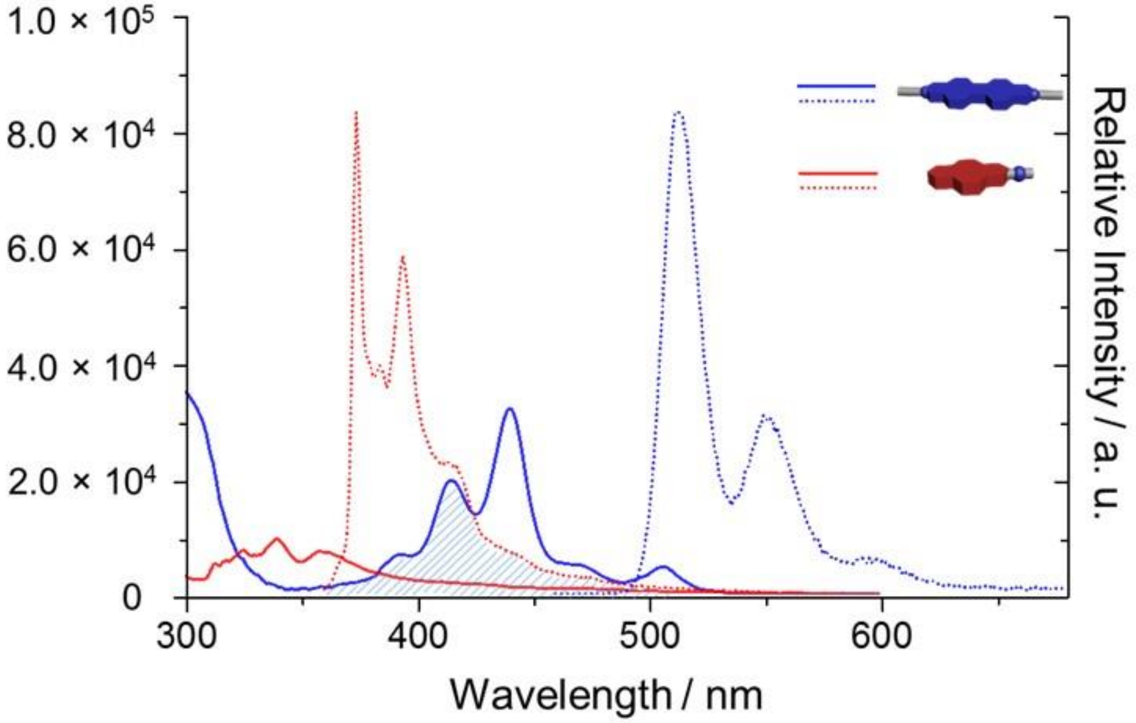

Supplementary Figure 45. UV-Vis absorbance (solid line) and fluorescence emission (dotted line) spectra of precursors **1**•Cl and **2**•2Cl in aqueous solution at 298K.

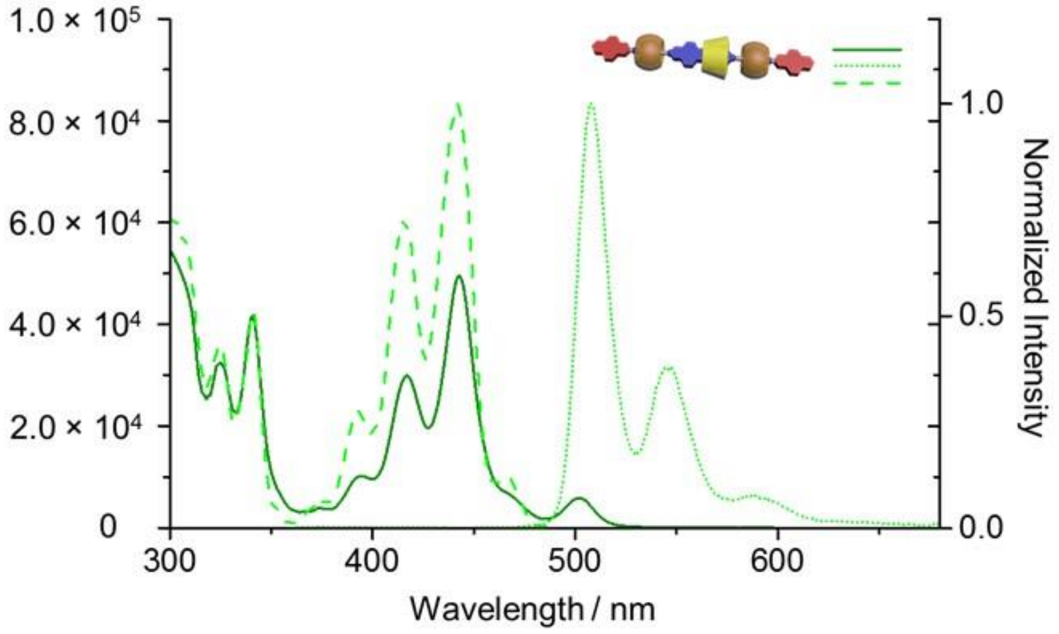

Supplementary Figure 46. UV-Vis absorbance (solid line), fluorescence excitation (dashed line) and emission (dotted line) spectra of the monomeric [4]rotaxane **R4**•4Cl in aqueous solution at 298K.

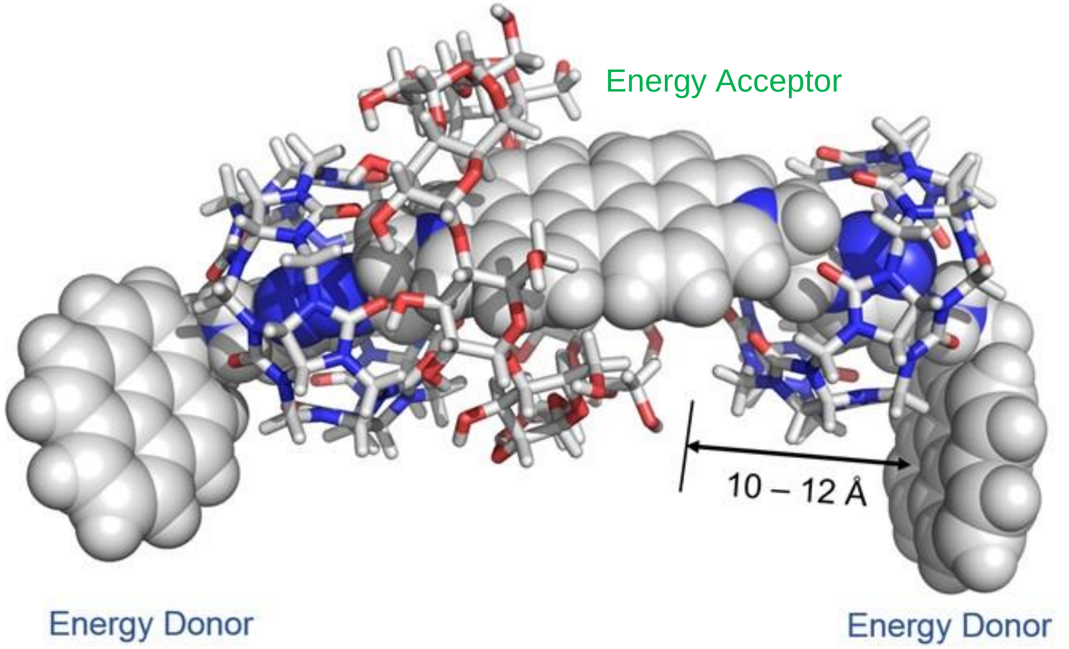

Supplementary Figure 47. Simulated structure of $\mathbf{R4}^{4+}$ using molecular mechanics (OPLS_2005). The orientation of γ-CD is fixed according to the NMR experiments.

Binding Studies between γ-CD and **R4**•4Cl

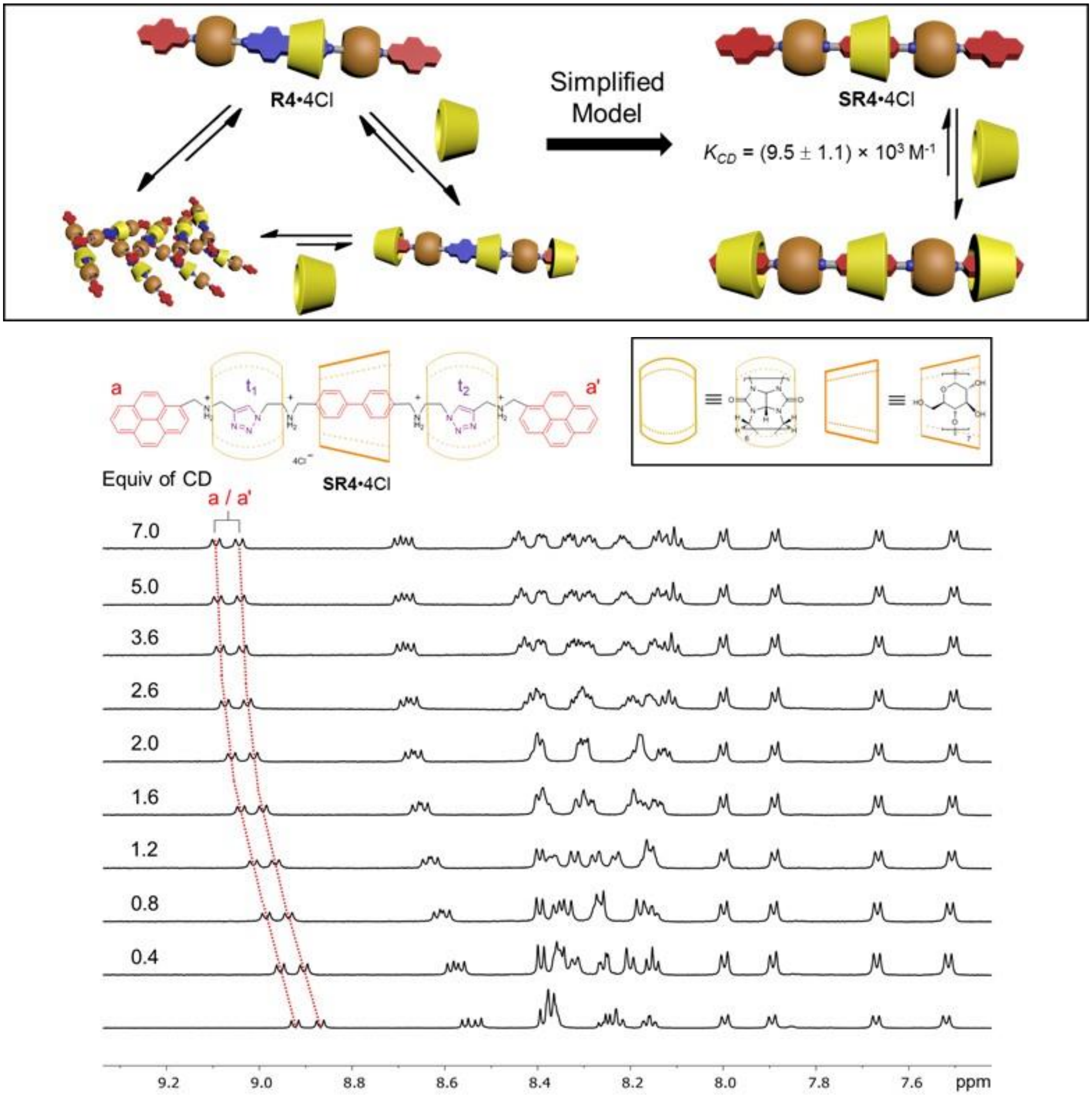

Supplementary Figure 48. Graphical representation (top) of the complicated binding behavior of **R4•**4Cl with γ-CD, and the binding behavior of the resulting simplified model compound **SR4•**4Cl, of which the binding sites resemble those of **R4•**4Cl, with γ-CD and the ^{1}H NMR spectra (bottom, 600 MHz, 298 K) of **SR4•**4Cl (initial concentration = 1 mM) with increasing amount of γ-CD (0.4 mM – 6.5 mM) in D_2O.

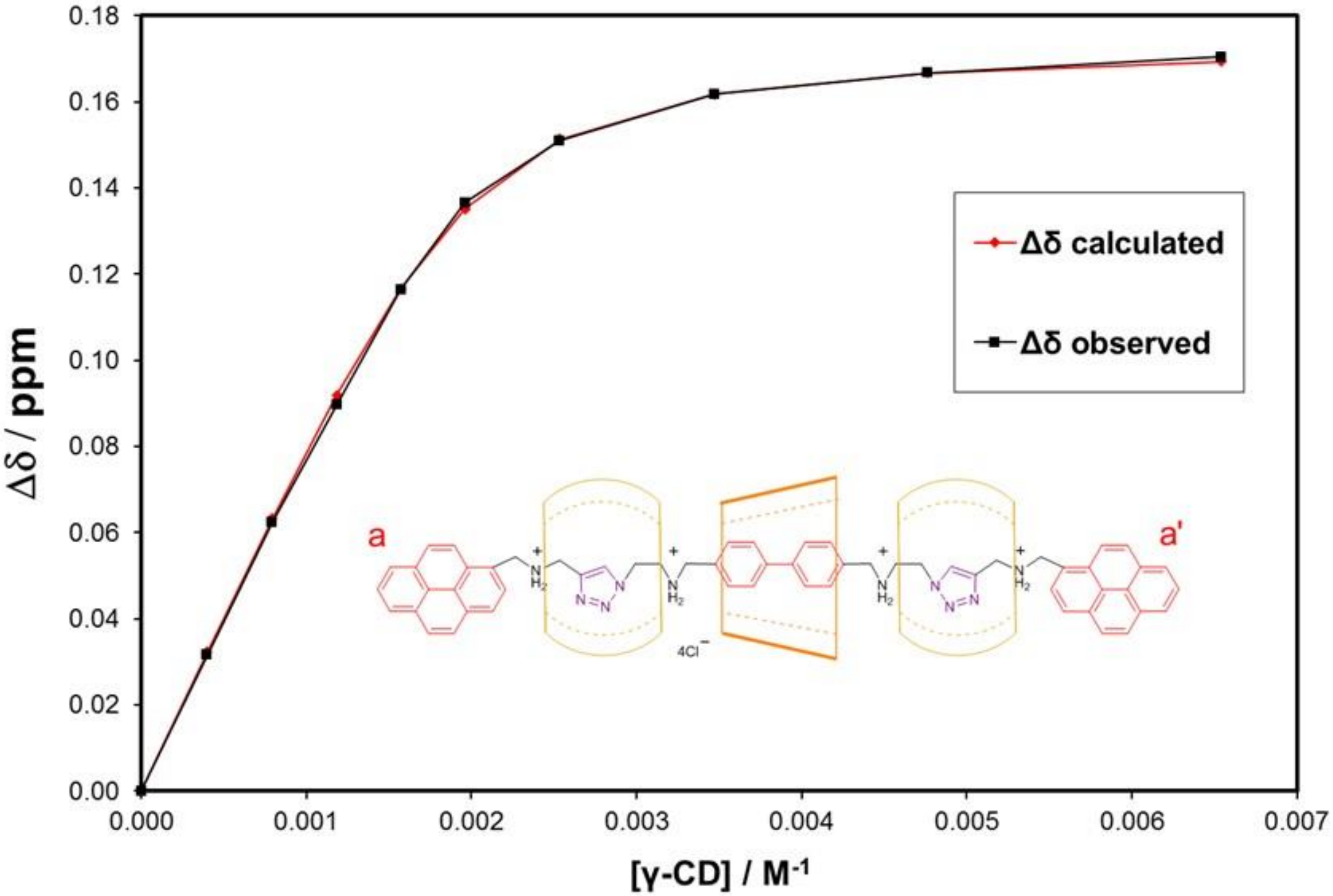

Supplementary Figure 49. Experimental and calculated values according to the average chemical shifts of the **a** and **a**' protons, assuming that the two pyrene binding sites are equivalent, for the ^{1}H NMR binding study of **SR4**•4Cl with γ-CD in D_2O. $K_a = (9.5 \pm 1.1) \times 10^3$ M^{-1}. The fact that the effective concentration of the binding sites is twice the concentration of **SR4**•4Cl has been accounted for in the calculation.

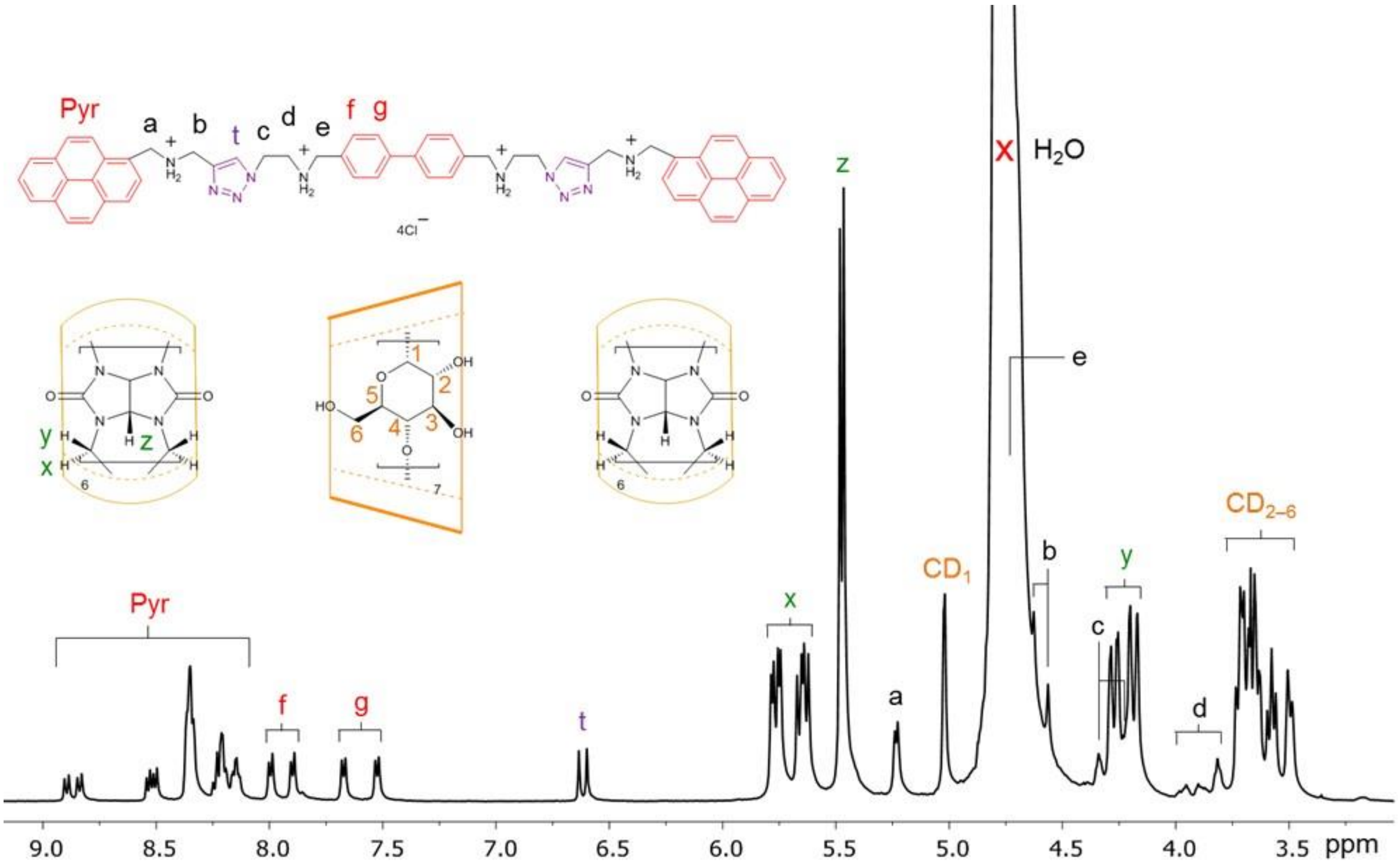

Supplementary Figure 50. ^{1}H NMR spectrum (500 MHz, D_2O, 25 °C) of **SR4•**4Cl. Rather than illustrate the structural formula of **SR4•**4Cl, its components with labeled protons are shown above. Since the presence of the CD ring introduces dissymmetry in the molecule, two sets of resonances are observed for protons Pyr, a ─ g, and x ─ z.

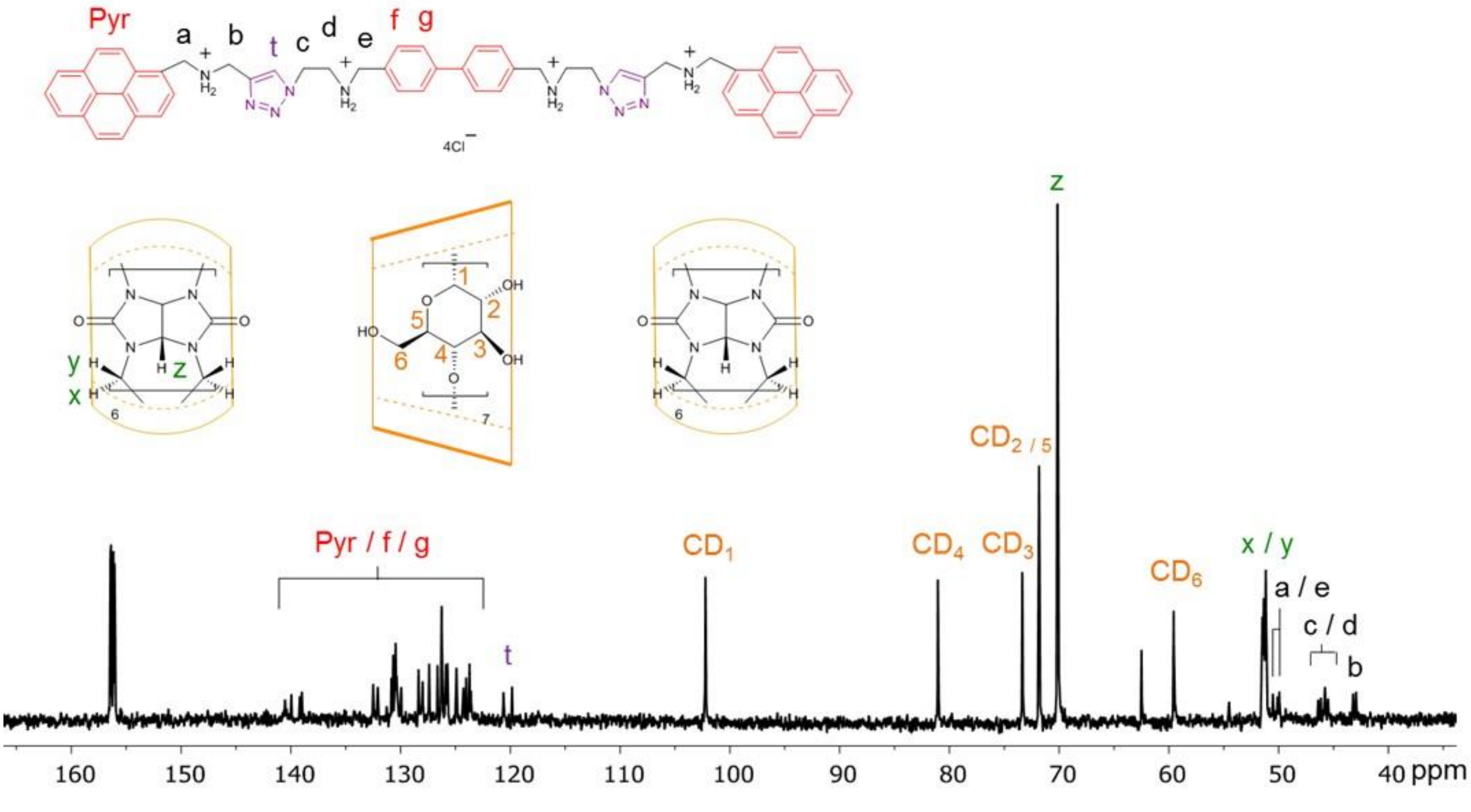

Supplementary Figure 51. ^{13}C NMR spectrum (500 MHz, D_2O, 25 °C) of **SR4•**4Cl. Rather than illustrate the structural formula of **SR4•**4Cl, its components with labeled protons are shown above.

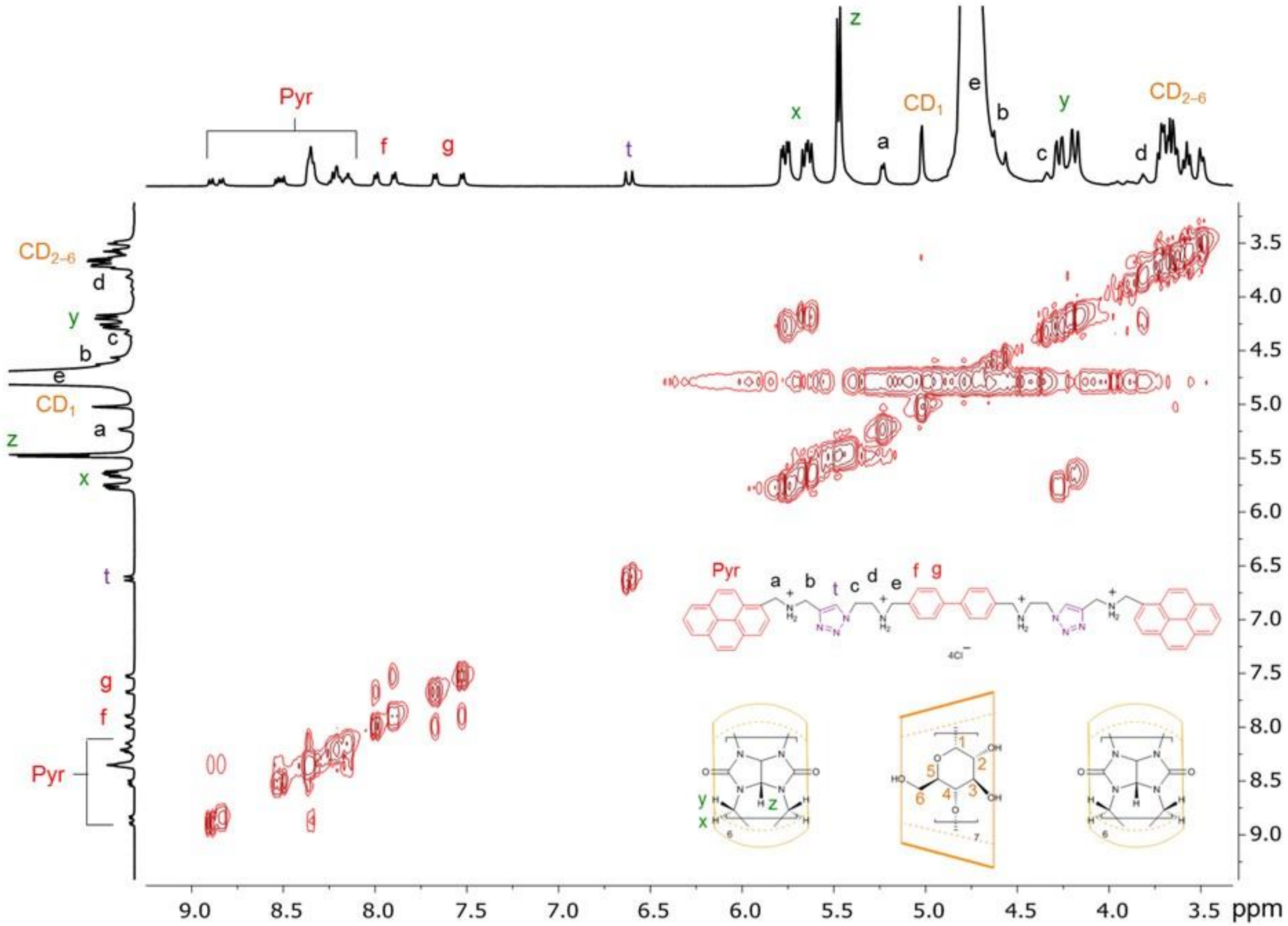

Supplementary Figure 52. ^1H-^1H COSY spectrum of **SR4•**4Cl (500 MHz, D_2O, 25 °C). Rather than illustrate the structural formula of **SR4•**4Cl, its components with labeled protons are shown above.

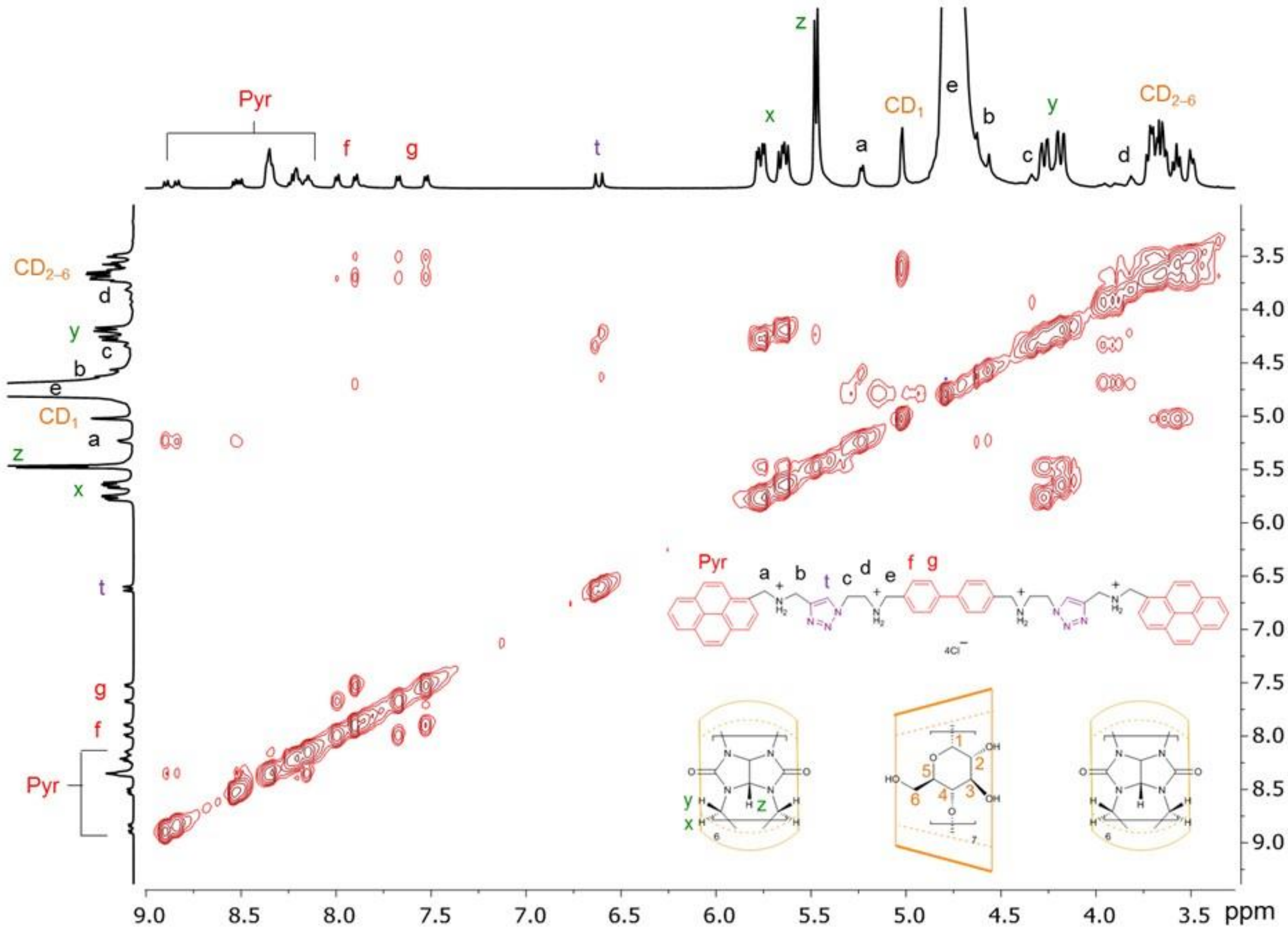

Supplementary Figure 53. ^1H-^1H NOESY spectrum of **SR4•**4Cl (500 MHz, D_2O, 25 °C). Rather than illustrate the structural formula of **SR4•**4Cl, its components with labeled protons are shown above.

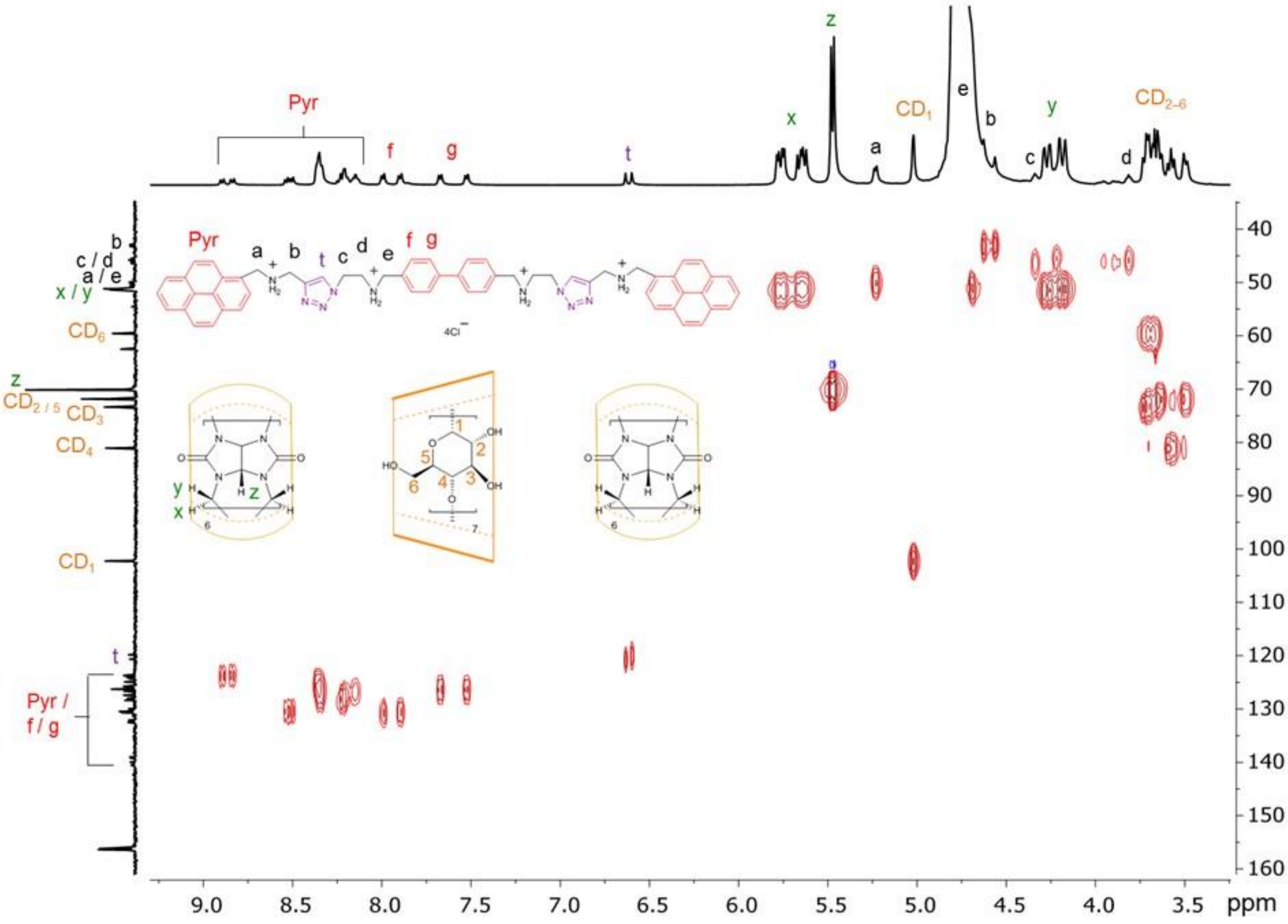

Supplementary Figure 54. ^{1}H-^{13}C HSQC spectrum of **SR4•**4Cl (500 MHz, D_2O, 25 °C). Rather than illustrate the structural formula of **SR4•**4Cl, its components with labeled protons are shown above.

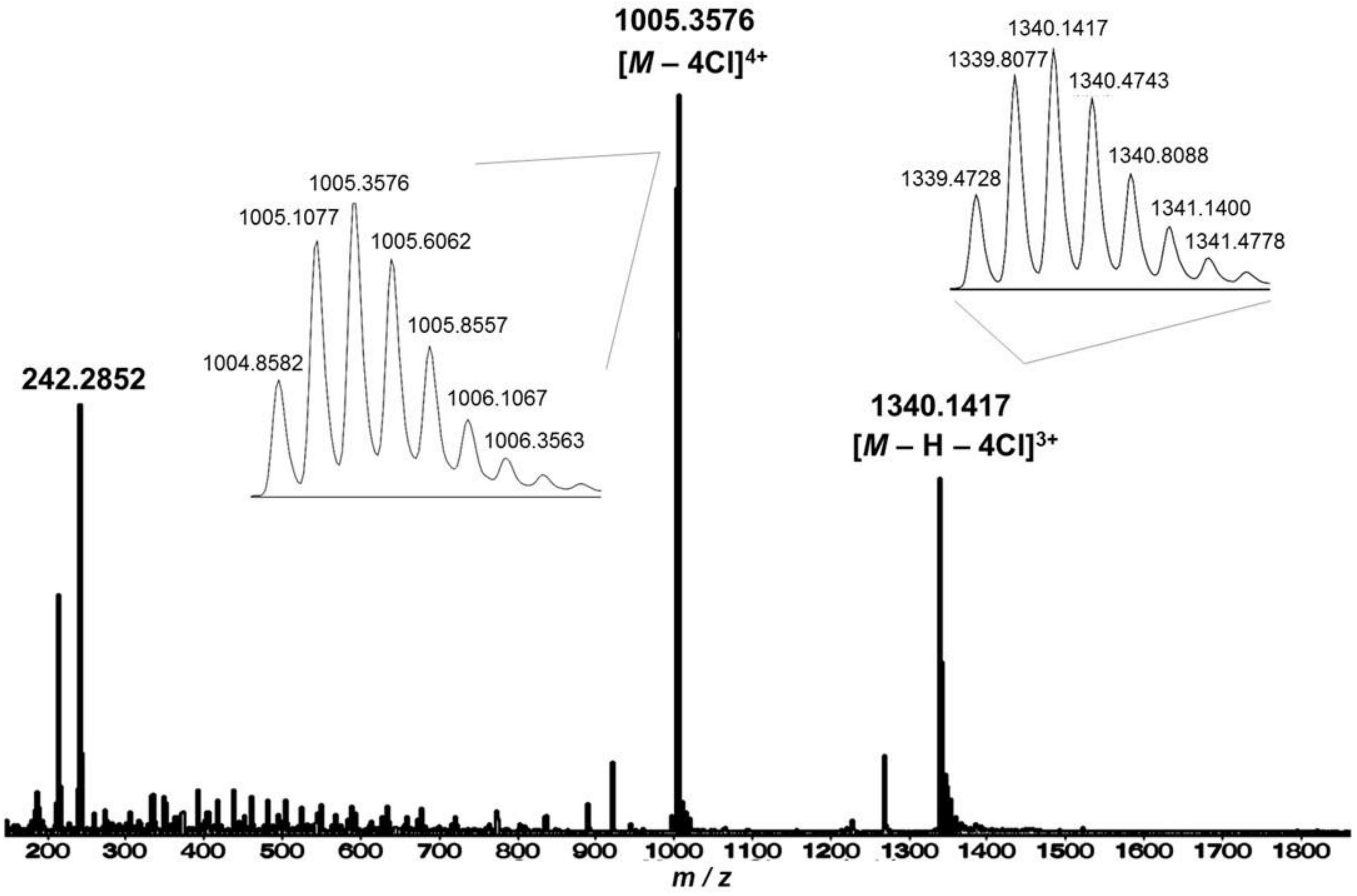

Supplementary Figure 55. High resolution ESI mass spectrum of **SR4•**4Cl

Binding Studies between γ-CD and Competing Agents Ad•Cl and AdMe•Cl

^{1}H NMR Binding Studies of Ad•Cl with γ-CD

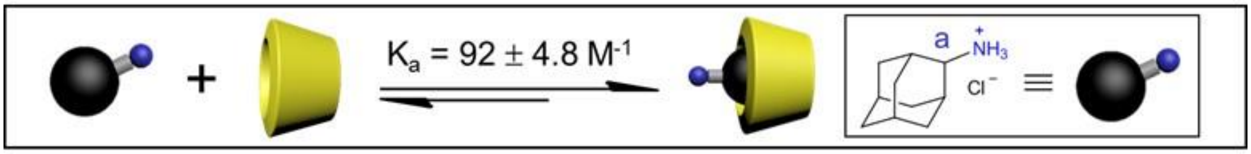

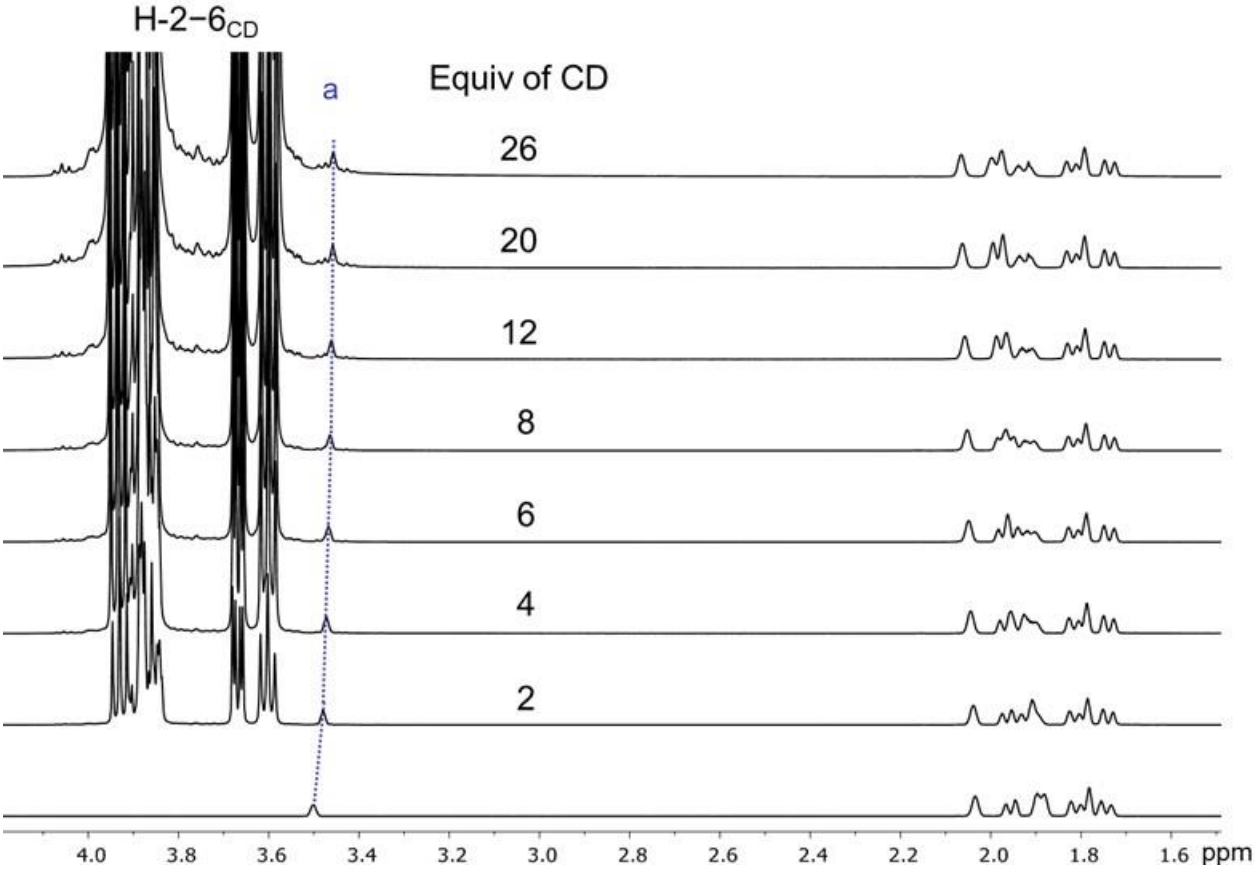

Supplementary Figure 56. Graphical representation (top) of the binding behavior of Ad•Cl with γ-CD and the ^{1}H NMR spectra (bottom, 600 MHz, 298 K) of AdMe•Cl (initial concentration = 5 mM) with the addition of γ-CD (9.5 mM – 78.8 mM) in D_2O.

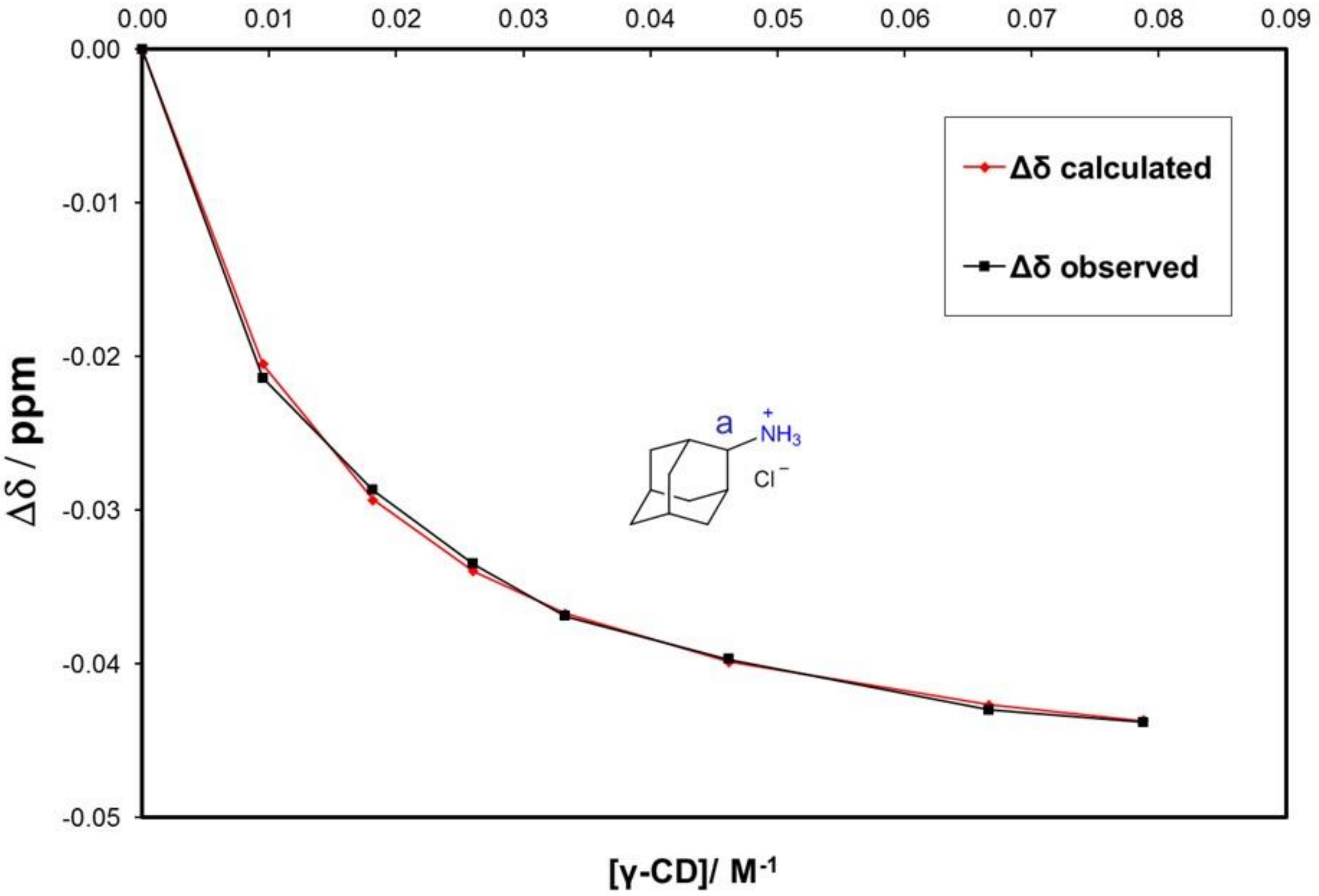

Supplementary Figure 57. Experimental and calculated values according to proton **a** for the ^{1}H NMR binding study of Ad•Cl with γ-CD in D_2O. K_a = 92 ± 4.8 M^{-1}.

^{1}H NMR Binding Studies of AdMe•Cl with γ-CD

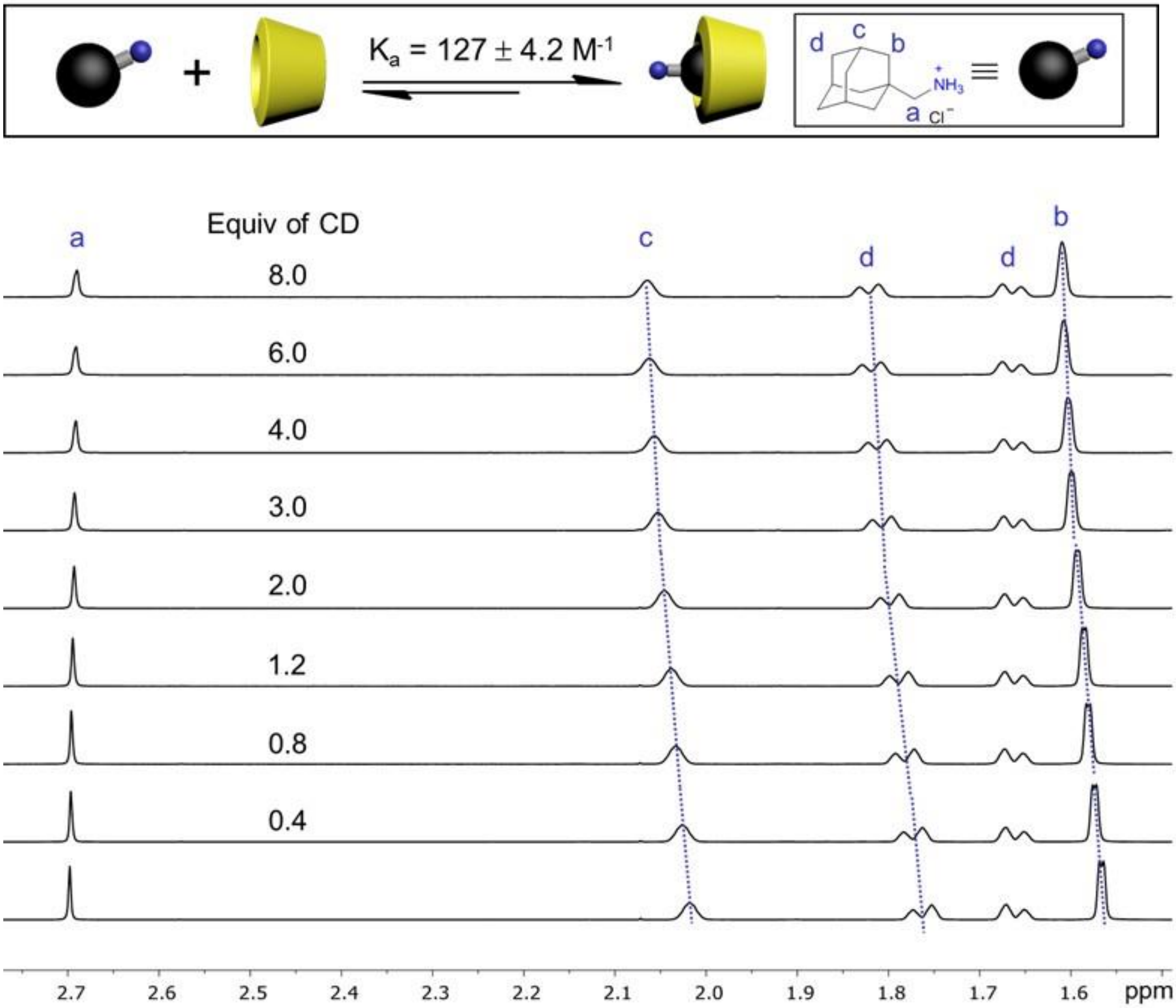

Supplementary Figure 58. Graphical representation (top) of the binding behavior of AdMe•Cl with γ-CD and the ^{1}H NMR spectra (bottom, 600 MHz, 298 K) of AdMe•Cl (initial concentration = 5 mM) with the addition of γ-CD (2.0 mM – 28.6 mM) in D_2O.

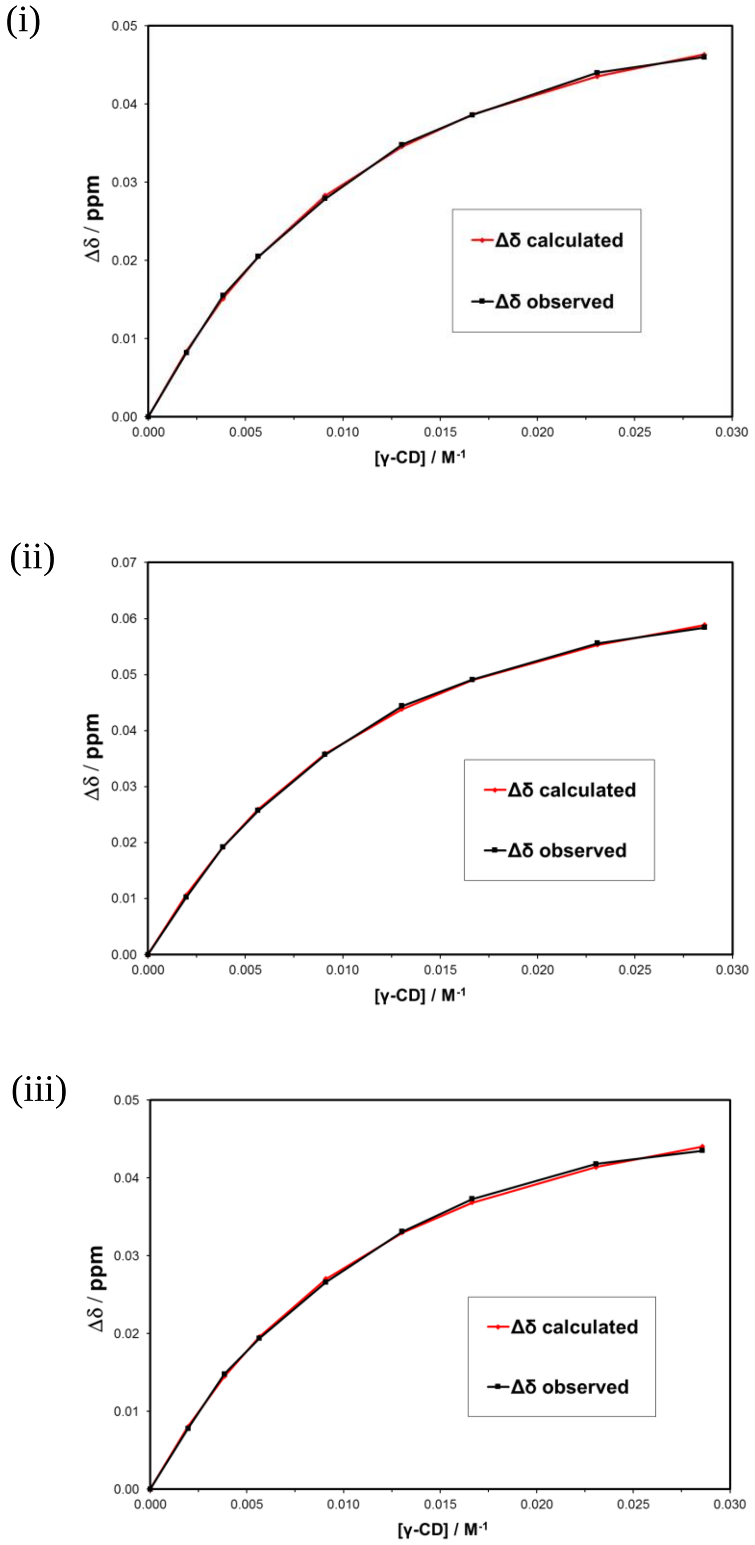

Supplementary Figure 59. Experimental and calculated values according to protons (i) **c**, (ii) **b** and (iii) **d** for the ^{1}H NMR binding study of AdMe•Cl with γ-CD in D_2O. K_a = 127 ± 4.2 M^{-1}.

Solid-State Photophysical Studies

Solid-State UV-Vis Spectroscopy

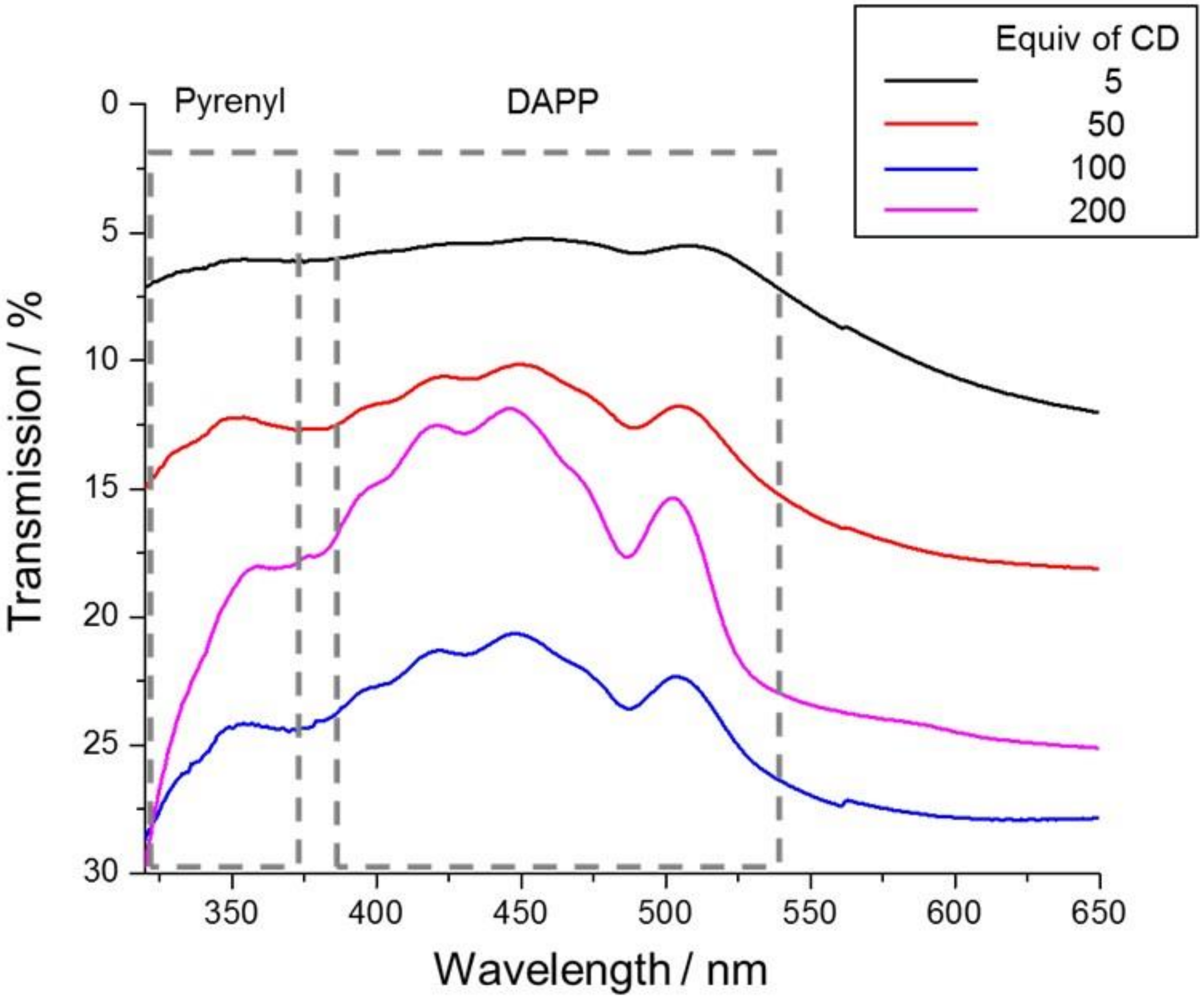

Supplementary Figure 60. Solid-state UV-Vis spectra of the powders of [4]rotaxanes **R4•**4Cl in the presence of different amounts of γ-CD (5 – 200 equiv).

Although the difference in thickness of the powder sample results in a difference in the transmission in the solid-state UV-Vis spectra, it is still clear that in the presence of increasing amounts of γ-CD, the spectra follow a similar trend (Supplementary Figure 37) to that exhibited in solution, indicating the de-aggregation of the **R4•**4Cl in the solid state.

Solid-State Fluorescence Spectroscopy

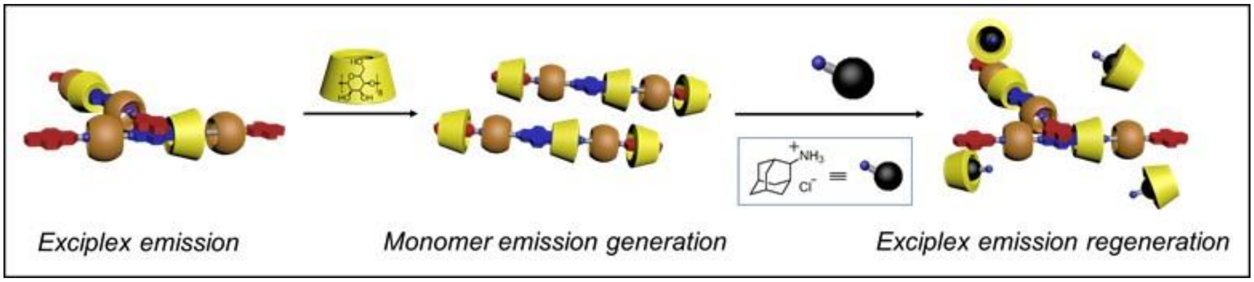

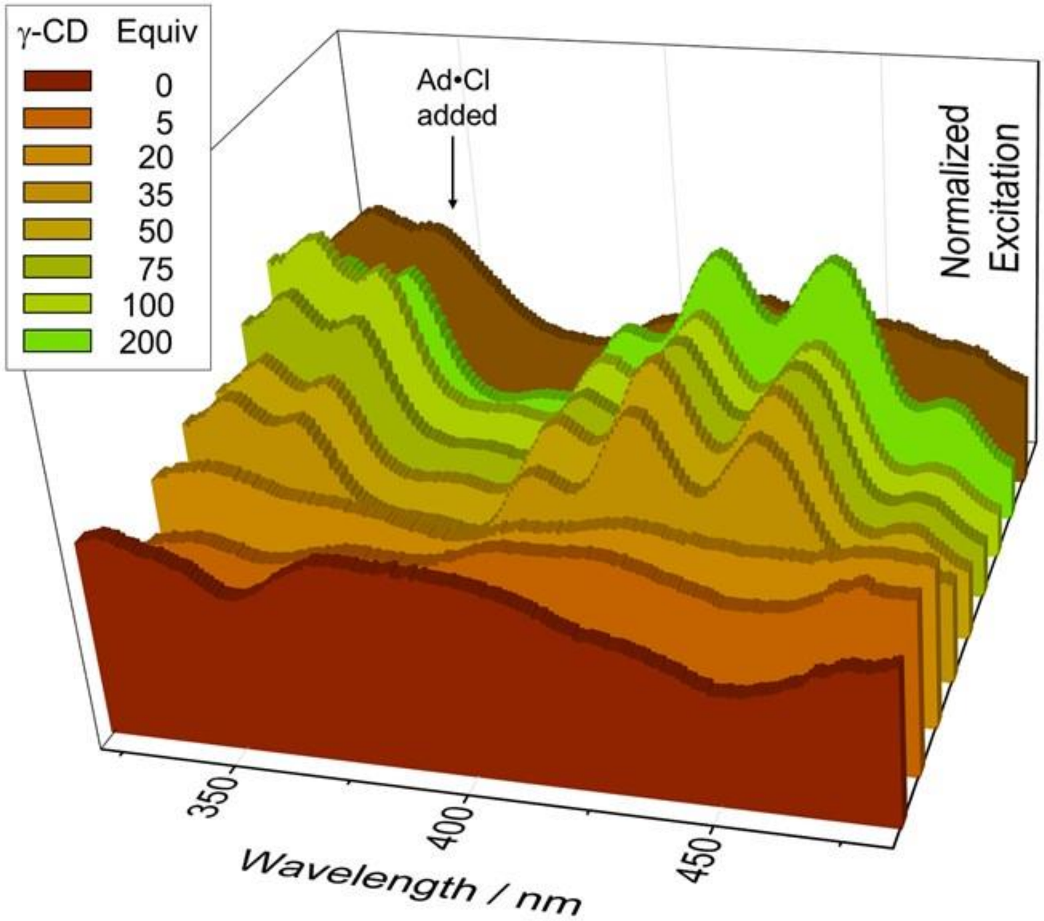

Supplementary Figure 61. Graphical representation (top) of the aggregation of [4]rotaxane **R4**•4Cl in the solid state and its de-aggregation upon the addition of an excess of γ-CD. The addition of Ad•Cl causes the rotaxane **R4**•4Cl to revert to its aggregated form. Solid-state fluorescent excitation spectra (bottom) of the [4]rotaxane **R4**•4Cl upon adding 0 – 200 equivalents of γ-CD and, in one case, 200 equivalents of both γ-CD and Ad•Cl.

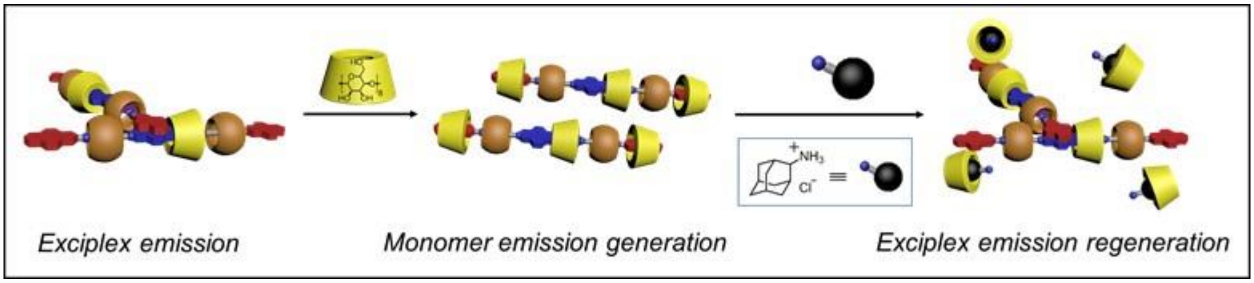

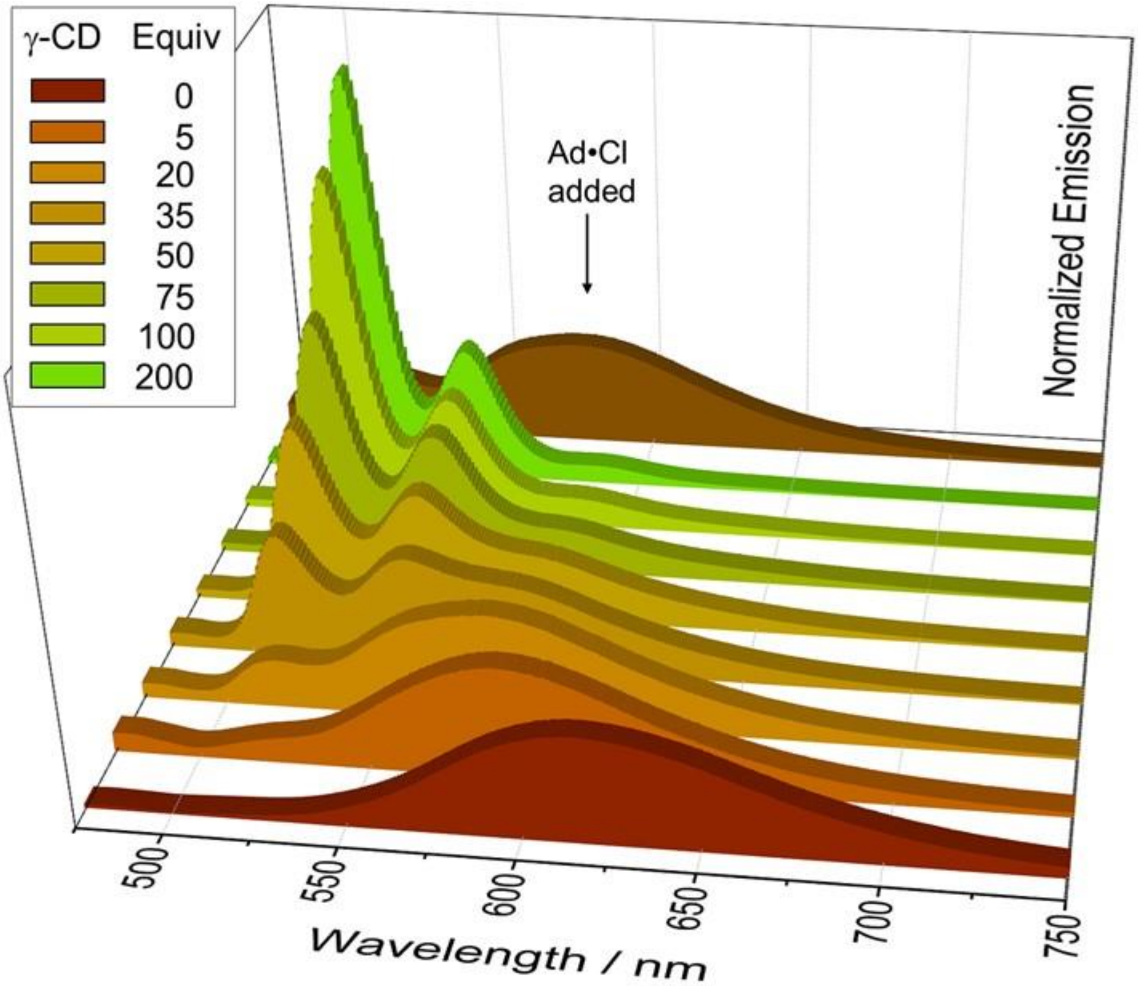

Supplementary Figure 62. Graphical representation (top) of the aggregation of [4]rotaxane **R4**•4Cl in the solid state and its de-aggregation upon the addition of an excess of γ-CD. The addition of Ad•Cl causes the rotaxane **R4**•4Cl to revert to its aggregated form. Solid-state fluorescent emission spectra (bottom, $\lambda_{excitation}$ = 447 nm) of the [4]rotaxane **R4**•4Cl upon adding 0 – 200 equivalents of γ-CD and, in one case, 200 equivalents of both γ-CD and Ad•Cl.

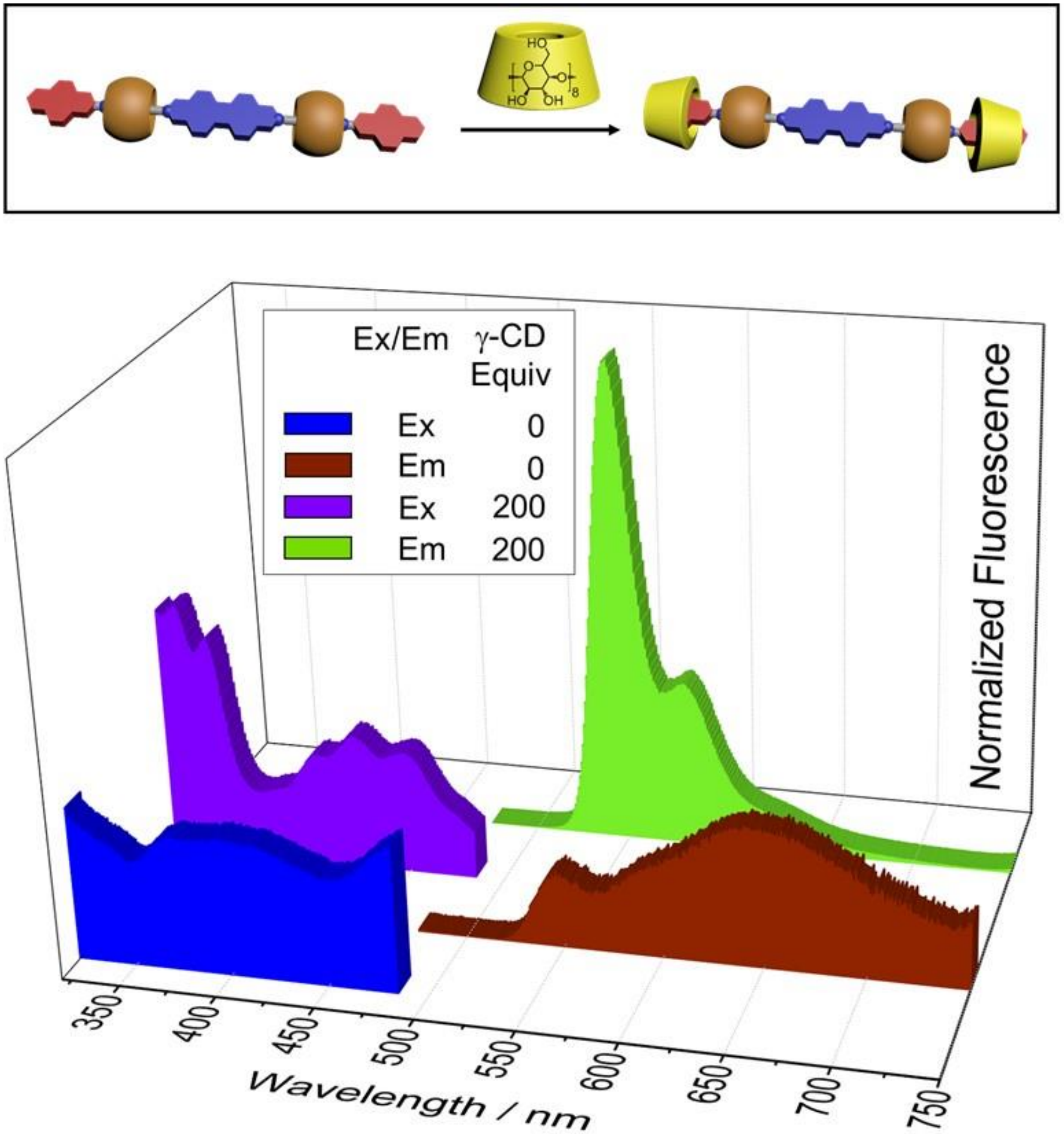

Supplementary Figure 63. Graphical representation (top) of the de-aggregation of [3]rotaxane **R3**•4Cl in the solid state upon the addition of an excess of γ-CD. Solid-state fluorescent excitation (Ex) and emission (Em) spectra (bottom, $\lambda_{excitation}$ = 341 nm) of the [3]rotaxane **R3**•4Cl upon adding 0 or 200 equivalents of γ-CD.

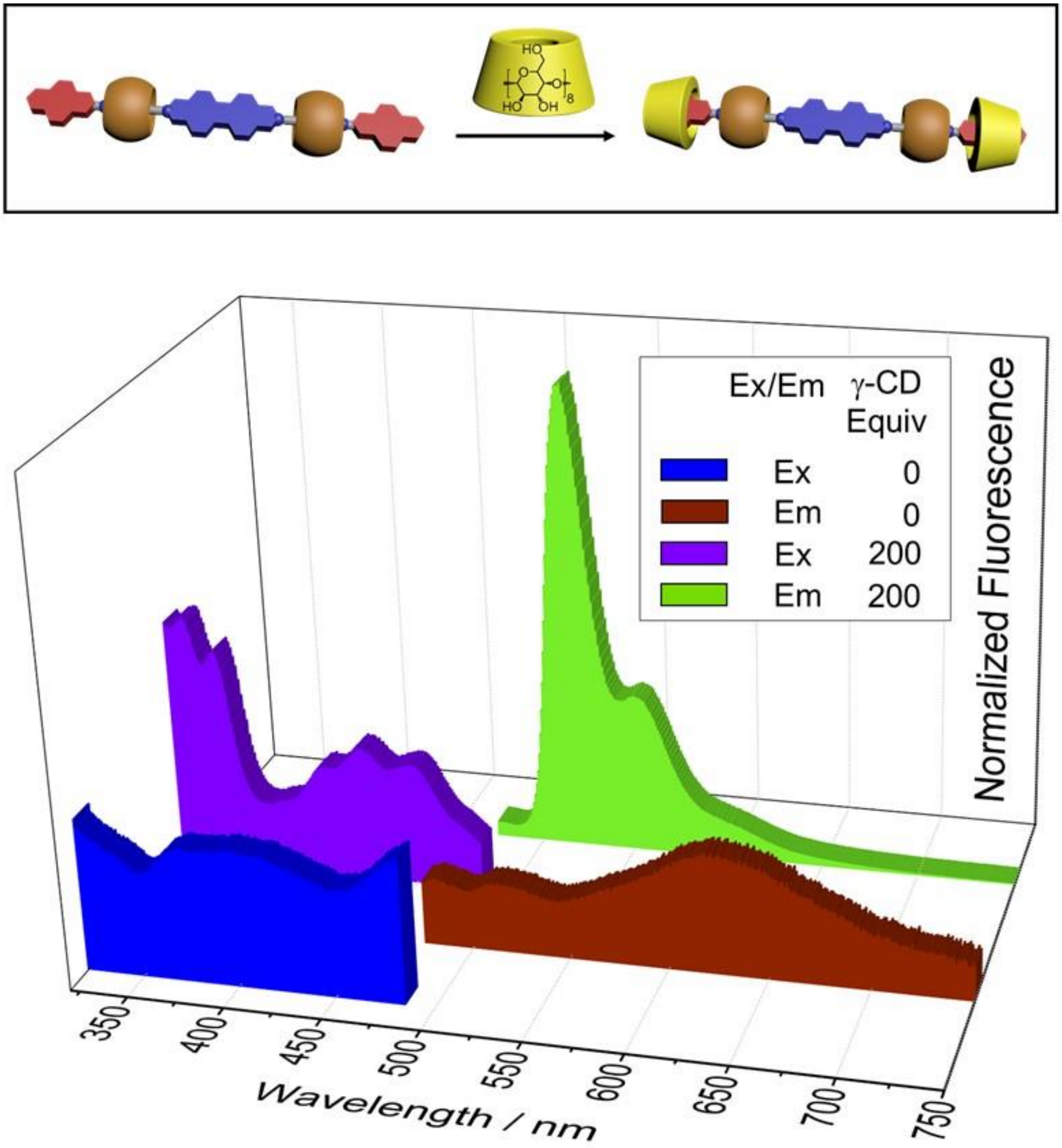

Supplementary Figure 64. Graphical representation (top) of the de-aggregation of [3]rotaxanes **R3**•4Cl in the solid state upon the addition of an excess of γ-CD. Solid-state fluorescent excitation (Ex) and emission (Em) spectra (bottom, $\lambda_{excitation}$ = 447 nm) of the [3]rotaxane **R3**•4Cl upon adding 0 or 200 equivalents of γ-CD.

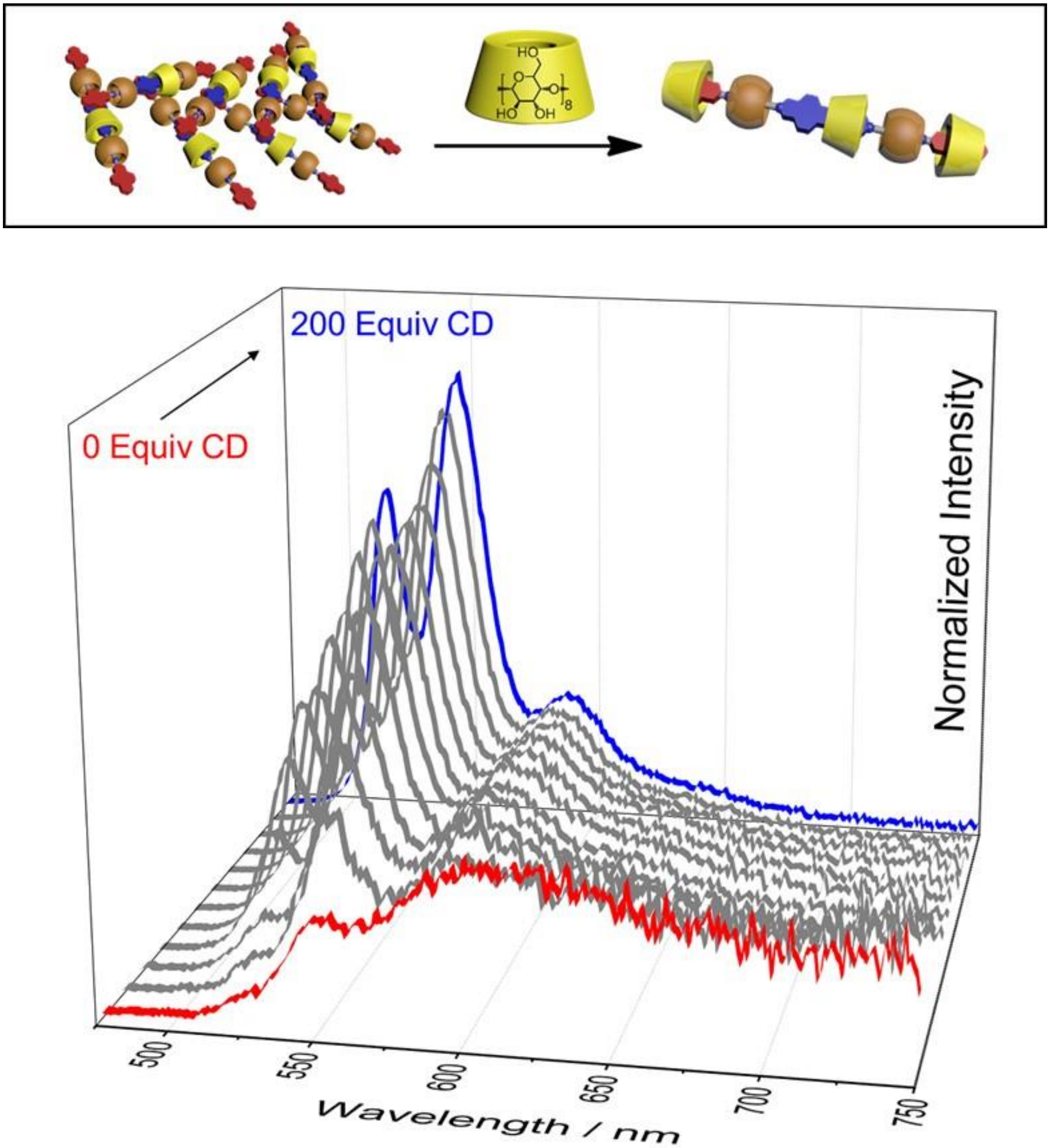

Supplementary Figure 65. Graphical representation (top) of the deaggregation the [4]rotaxane **R4**•4Cl in response to addition of γ-CD. Normalized fluorescence emission spectra (bottom, $\lambda_{excitation}$ = 341 nm) of up to 200 equiv of γ-CD titrated in a 500 μM aqueous solution the hetero[4]rotaxane **R4**•4Cl measured at 25 ^{o}C.

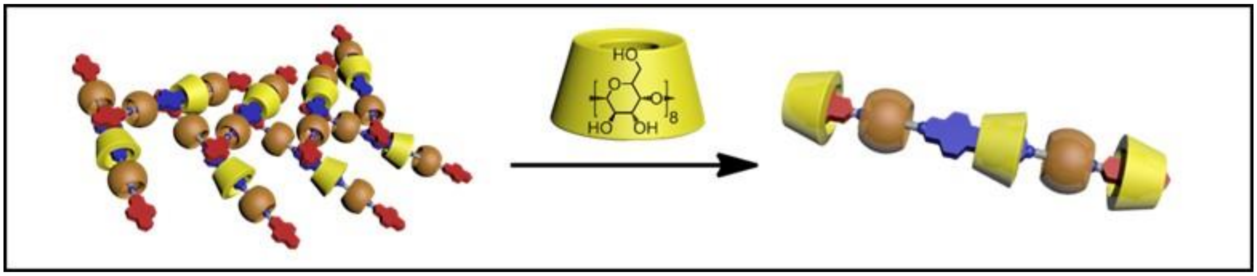

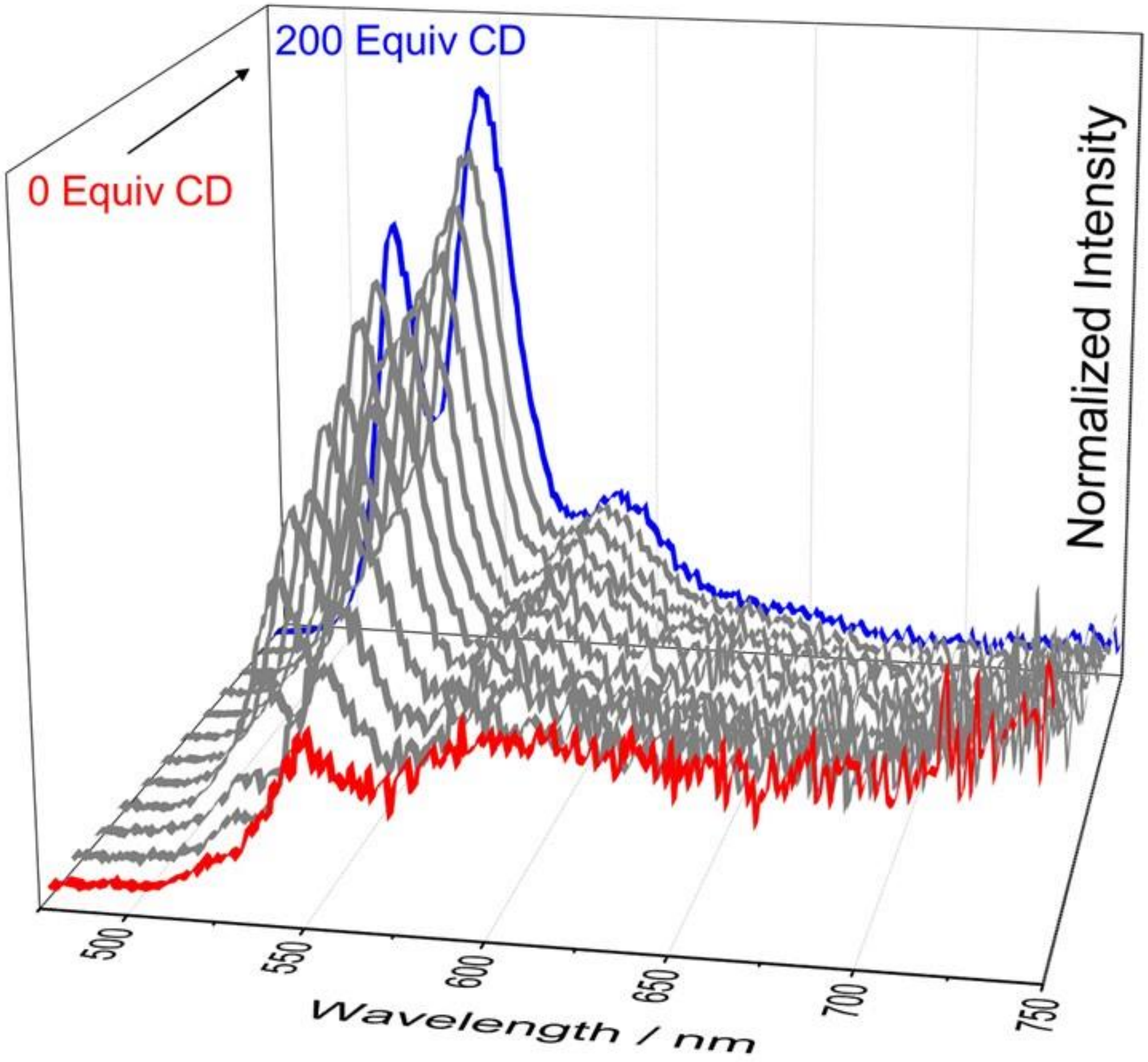

Supplementary Figure 66. Graphical representation (top) of the deaggregation the [4]rotaxane **R4**•4Cl in response to addition of γ-CD. Normalized fluorescence emission spectra (bottom, $\lambda_{excitation}$ = 447 nm) of up to 200 equiv of CD titrated in a 500 μM aqueous solution the hetero[4]rotaxane **R4**•4Cl measured at 25 ^{o}C.

Ink-Writing Tests

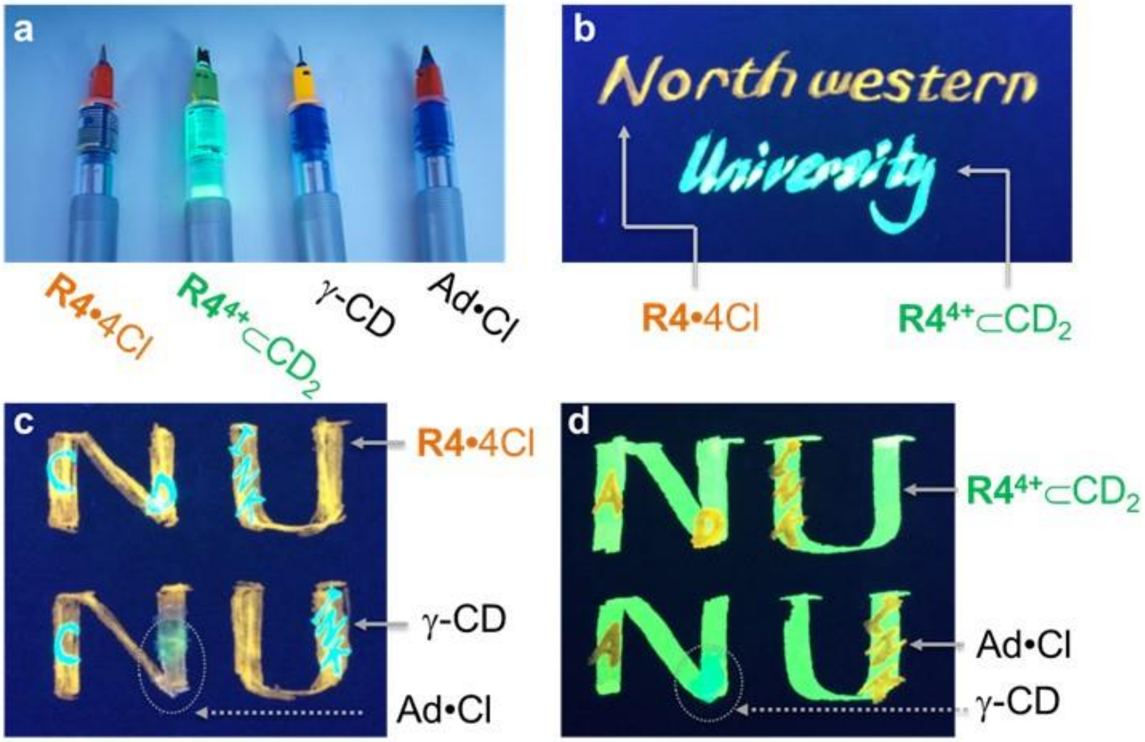

Supplementary Figure 67. **Writing test using aqueous solutions of the [4]rotaxane R4•4Cl, the complex $R4^{4+}$⊂γ-CD_2, γ-CD and Ad•Cl as inks**. **a**, Four fountain pens filled with the aqueous solutions of **R4**•4Cl, **$R4^{4+}$**⊂γ-CD_2, γ-CD and Ad•Cl as inks, respectively, under UV LED light. **b**, Photograph of the phrase 'Northwestern University' written on rag paper (uncoated) and illuminated with UV light (λ = 254 nm), in which 'Northwestern' was written using **R4**•4Cl ink and 'University' was written using **$R4^{4+}$**⊂γ-CD_2 ink. **c**, The letters 'NU' written twice on rag paper (uncoated) using **R4**•4Cl ink under UV light (λ = 254 nm). γ-CD ink was applied to write "CD INK" on top of the "NU" and the character 'D' of the 'CD INK' on the bottom line was erased after applying the Ad•Cl ink. **d**, The letters 'NU' written twice on rag paper (uncoated) using **$R4^{4+}$**⊂γ-CD_2 ink under UV light (λ = 254 nm). Ad•Cl ink was applied to write "AD INK" on top of the "NU" and the character 'D' of the 'AD INK' on the bottom line was erased after applying the γ-CD ink.

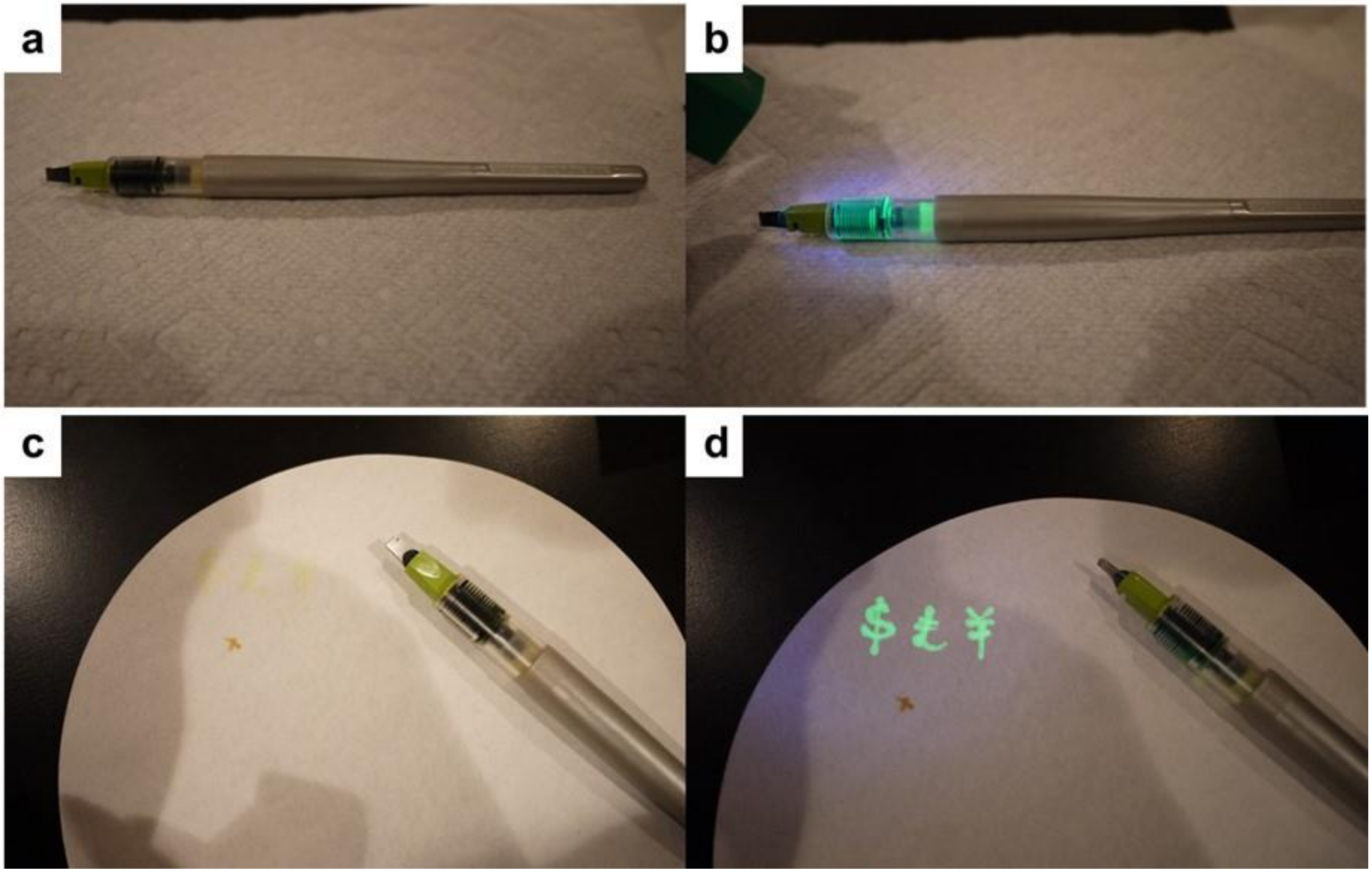

Supplementary Figure 68. Photo images of a fountain pen filled with **R4**$^{4+}\subset\gamma$-CD$_2$ ink under visible light (**a**) and UV light (**b**). Photo images of a writing test on a filter paper using **R4**$^{4+}\subset\gamma$-CD$_2$ ink under visible light (**c**) and UV light (**d**). "$, £, and ¥" symbol were written using the **R4**$^{4+}\subset\gamma$-CD$_2$ ink, "×" symbol were written using a commercial counterfeit banknote detection pen.

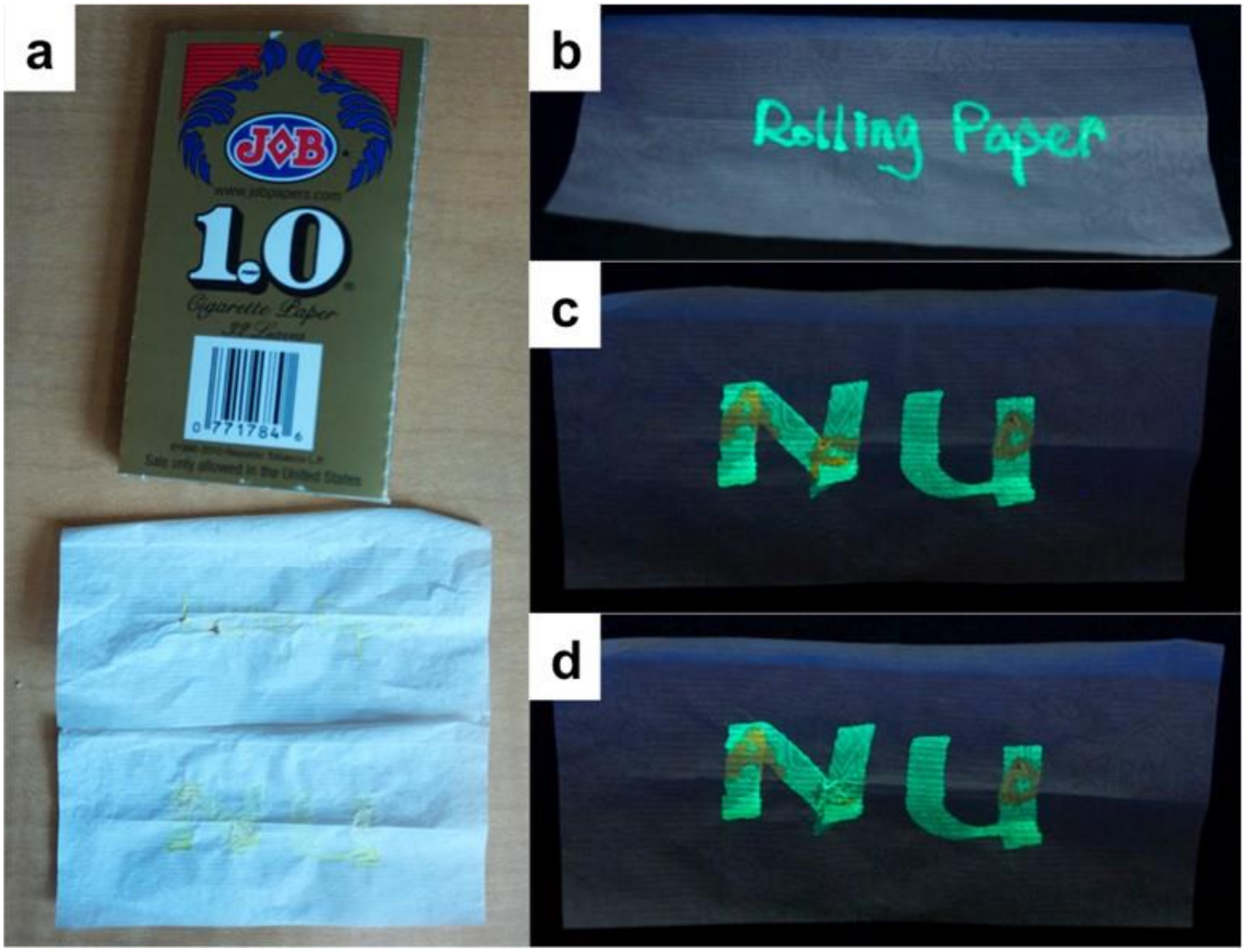

Supplementary Figure 69. (a) Photo images of cigarette rolling paper package and two rolling papers under visible light. “Rolling Paper” and “NU” were written on these papers with **R4**$^{4+}$$\subset$γ-CD$_2$ ink. (b) Photo image of “Rolling Paper” under UV light. (c) Photo image of “NU” under UV light, “AFD” was written on top of “NU” using Ad•Cl ink. (d) Photo image of “NU” under UV light, “F” was erased from “AFD” using γ-CD ink.

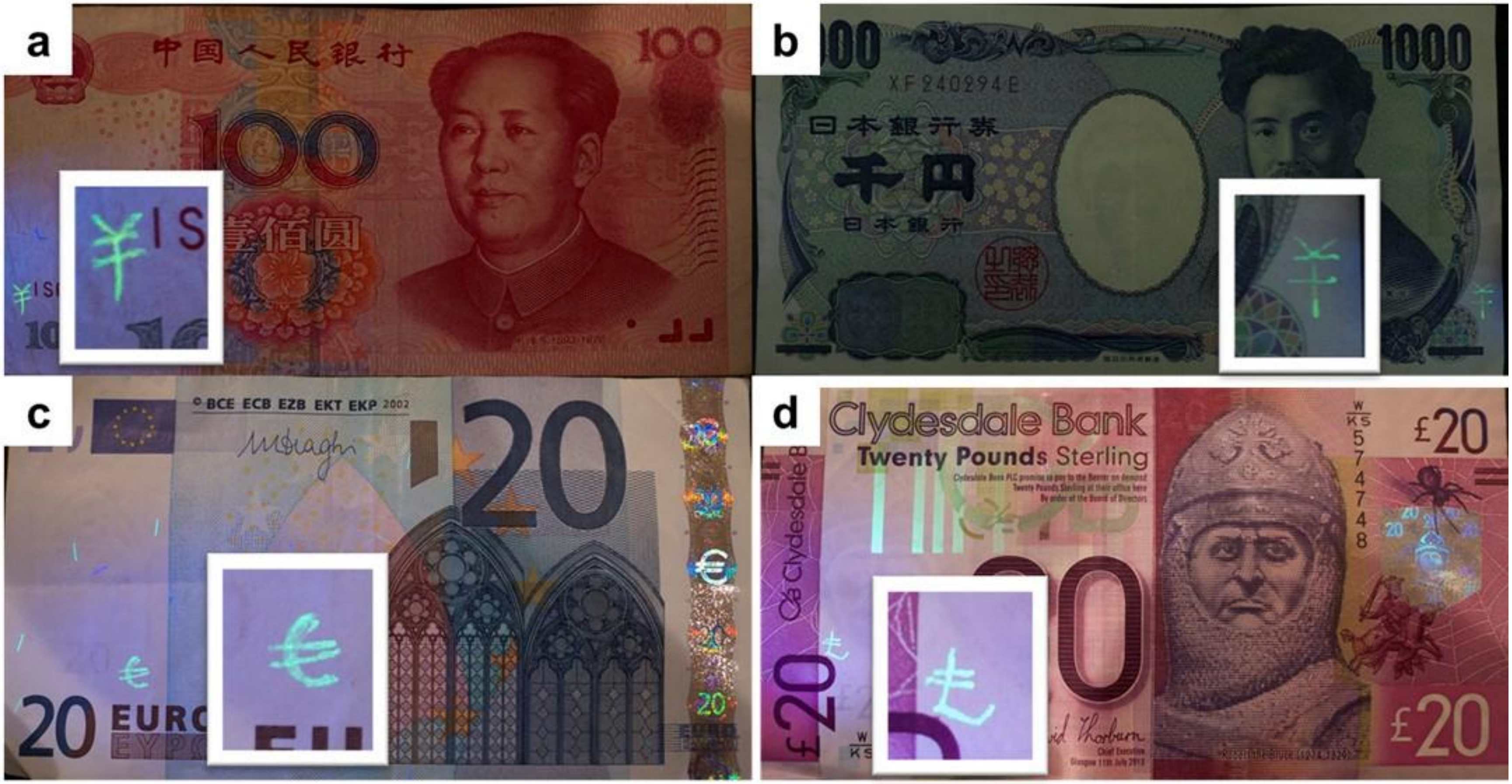

Supplementary Figure 70. Photo images of banknote tests using a fountain pen filled with $\mathbf{R4}^{4+}\subset\gamma\text{-CD}_2$ ink. Currency symbols drawn on genuine banknotes, Chinese Yuan (a), Japanese Yen (b), Euro (c) and British Sterling Pounds (d), show green fluorescence under UV LED light.

Monochromic Printing Tests

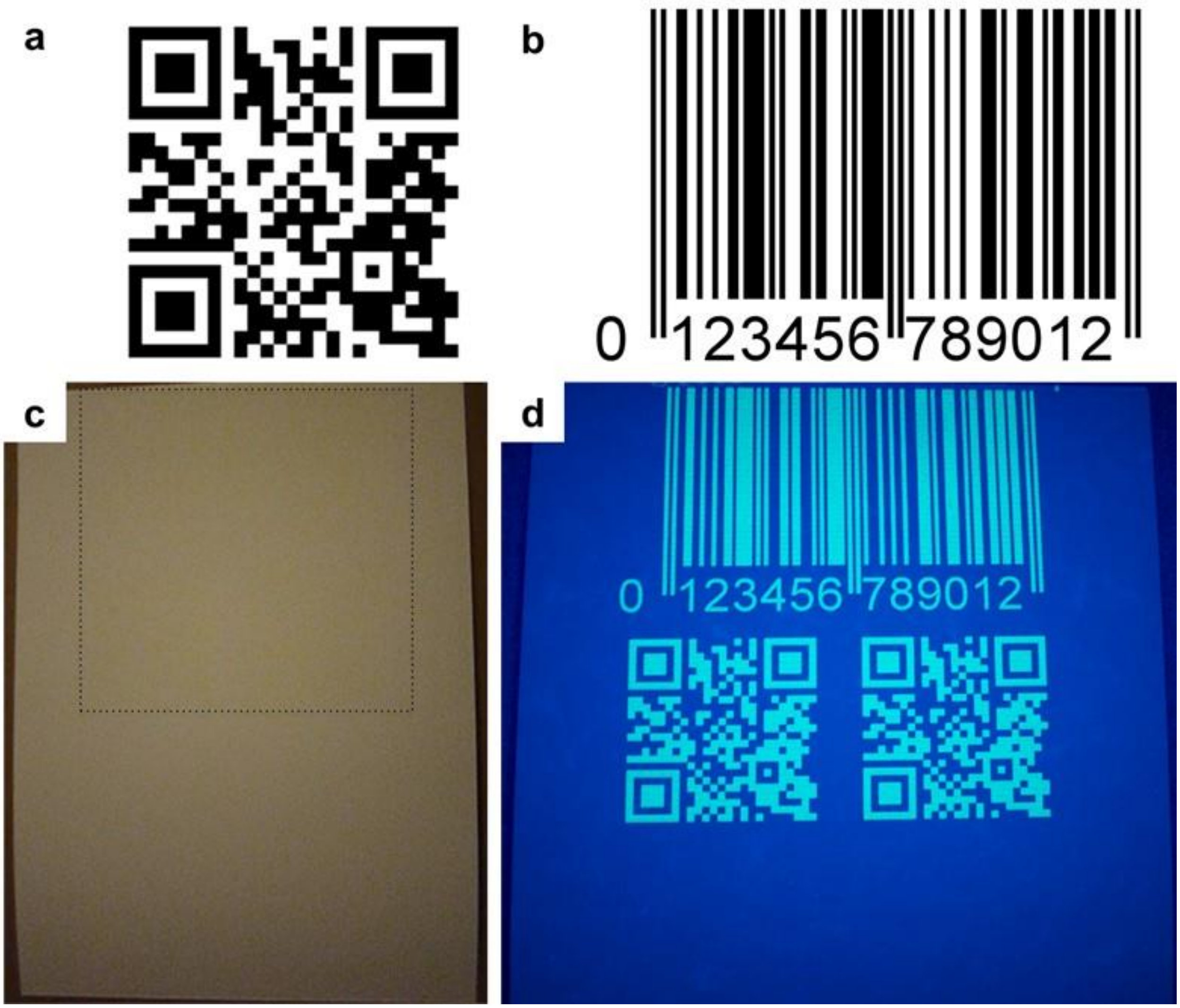

Supplementary Figure 71. Original digital images, QR code (a) and barcode (b), for print tests. (c) Photo images of the printed document containing two QR codes (identical) and one barcode under visible light. Dashed line area indicates the printed area. (d) Photo image of the QR codes and barcode under UV light (λ = 365 nm), printed with the $\mathbf{R4}^{4+}\subset\gamma\text{-CD}_2$ ink.

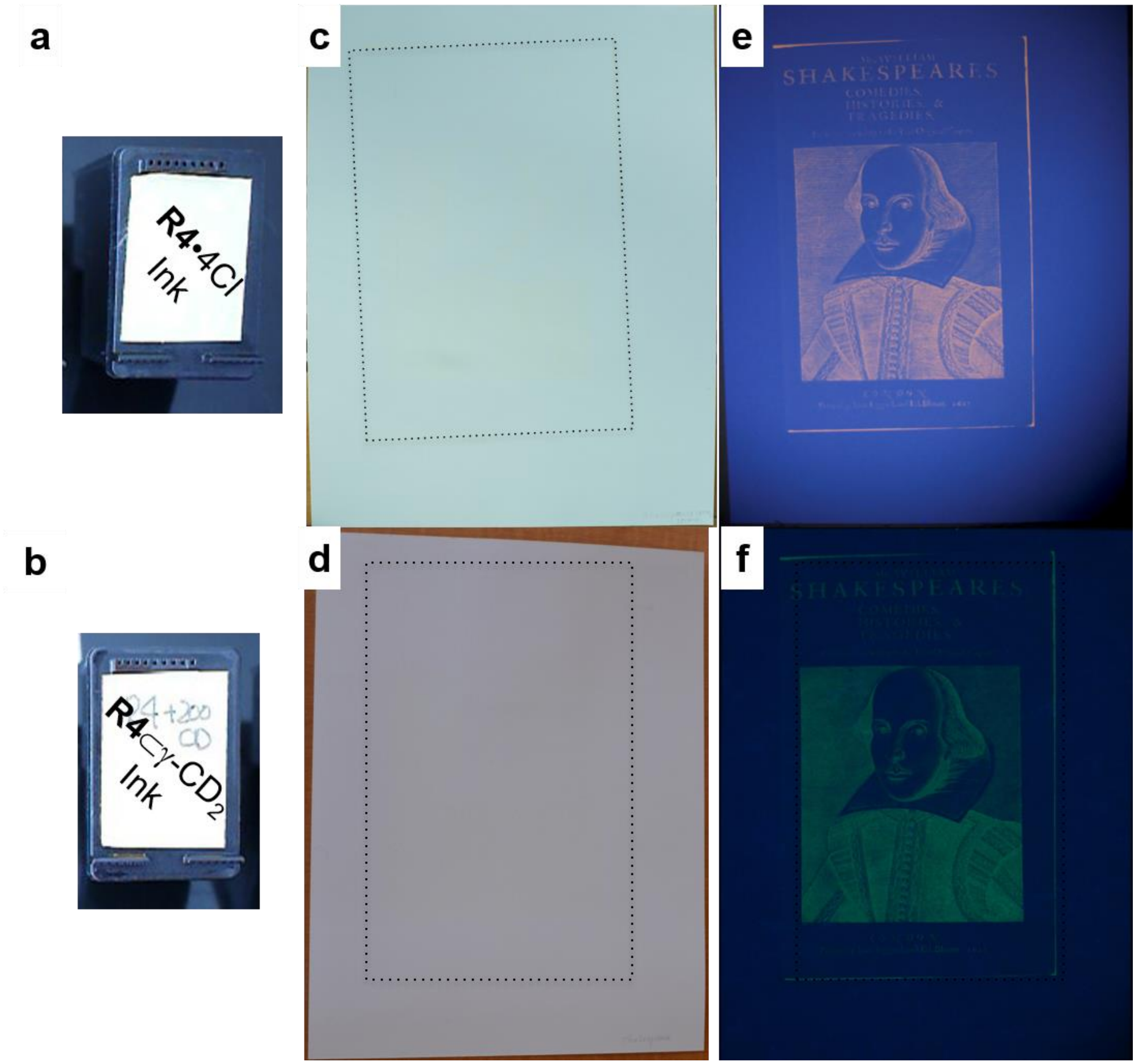

Supplementary Figure 72. Photo images of customised HP60 black ink cartridge filled with **R4**•4Cl (a) and **R4**$^{4+}$$\subset\gamma$-$CD_2$ ink (b), respectively. Photo images of the “Shakespeare cover” printed on rag paper with **R4**•4Cl ink cartridge under visible light (c) and UV light (e). Photo images of the “Shakespeare cover” printed on rag paper with **R4**$^{4+}$$\subset\gamma$-$CD_2$ ink cartridge under visible light (d) and UV light (f). Dashed line area indicates the printed area.

Polychromic Printing Experiments

RG (Red-Green) Printing

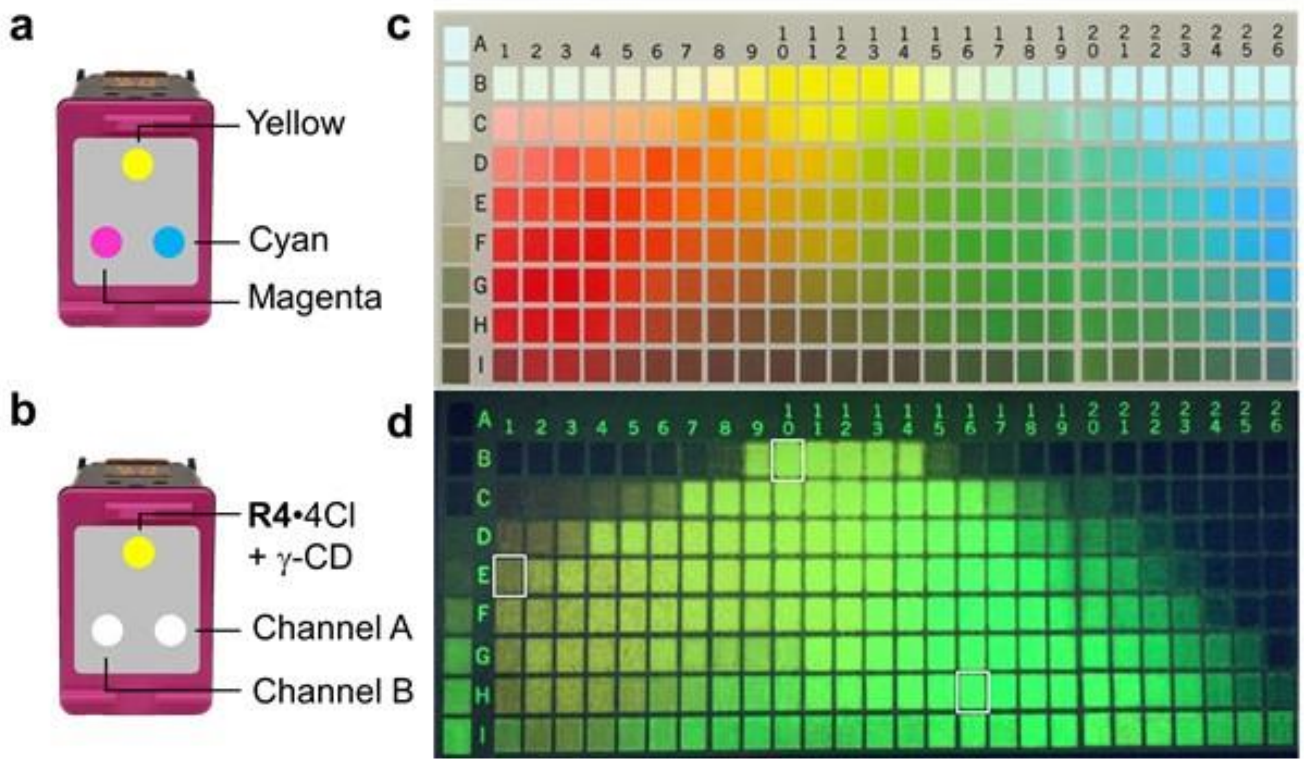

Supplementary Figure 73. Multi-coloured photo images of documents produced by an inkjet printer with customised inkjet cartridges. **a**, **b**, Graphical representation of a commercial 3-colour (yellow, magenta and cyan) ink cartridge (**a**), and a customised colour ink cartridge (**b**), in which the 3-colour channels filled by **R4**•4Cl/γ-CD (2mL, **R4**•4Cl: 0.5 mM, γ-CD: 25 mM), Ad•Cl (Channel B: 2 mL, 100 mM), and γ-CD (Channel A: 2 mL, 100 mM) respectively. **c**, Colour grid image printed using a commercial

inkjet printer with 3-colour ink cartridge. **d**, Colour grid image taken in UV (λ = 365 nm) produced by ink cartridge (**b**) using an inkjet printer.

RGB (Red-Green-Blue) Printing

In order to perform RGB printing, PyMe•Cl was selected to provide blue fluorescent emission upon UV irradiation. There are two ways to provide RGB printing shown as follows:

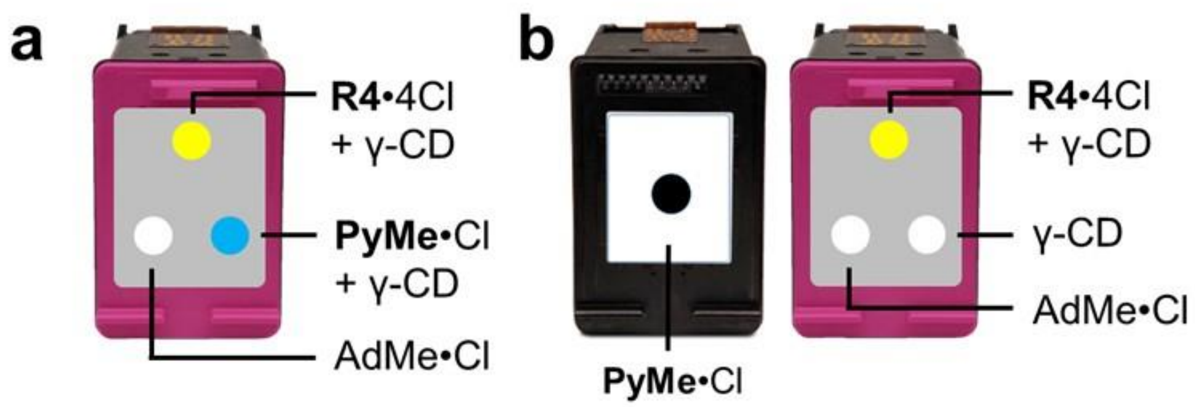

Supplementary Figure 74. RGB printing inkjet printer cartridges setup. (**a**) tri-colour inkjet catridge loaded with **R4**•4Cl/γ-CD (2 mL, **R4**•4Cl: 0.25 mM, γ-CD: 10 mM), AdMe•Cl (2 mL, 50 mM), and PyMe•Cl/γ-CD (2 mL, **PyMe**•Cl: 1 mM, γ-CD: 25 mM), in three channels respectively. (**b**) A combination use of black inkjet cartridge loaded with the aqueous solution of **PyMe**•Cl (1 mM) and tri-colour inkjet cartridge with **R4**•4Cl/γ-CD (2 mL, **R4**•4Cl: 0.25 mM, γ-CD: 10 mM), AdMe•Cl (2 mL, 50 mM), and γ-CD (2 mL, γ-CD: 25 mM), in three channels respectively.

In practice, both of the settings could provide a wide spectrum of colours, ranging from purple to red fluorescence upon UV irradiation. The colour distributions (Supplementary Figure 79 b and c) of the printed images are very different, however, by using these two types of RBG printing settings.

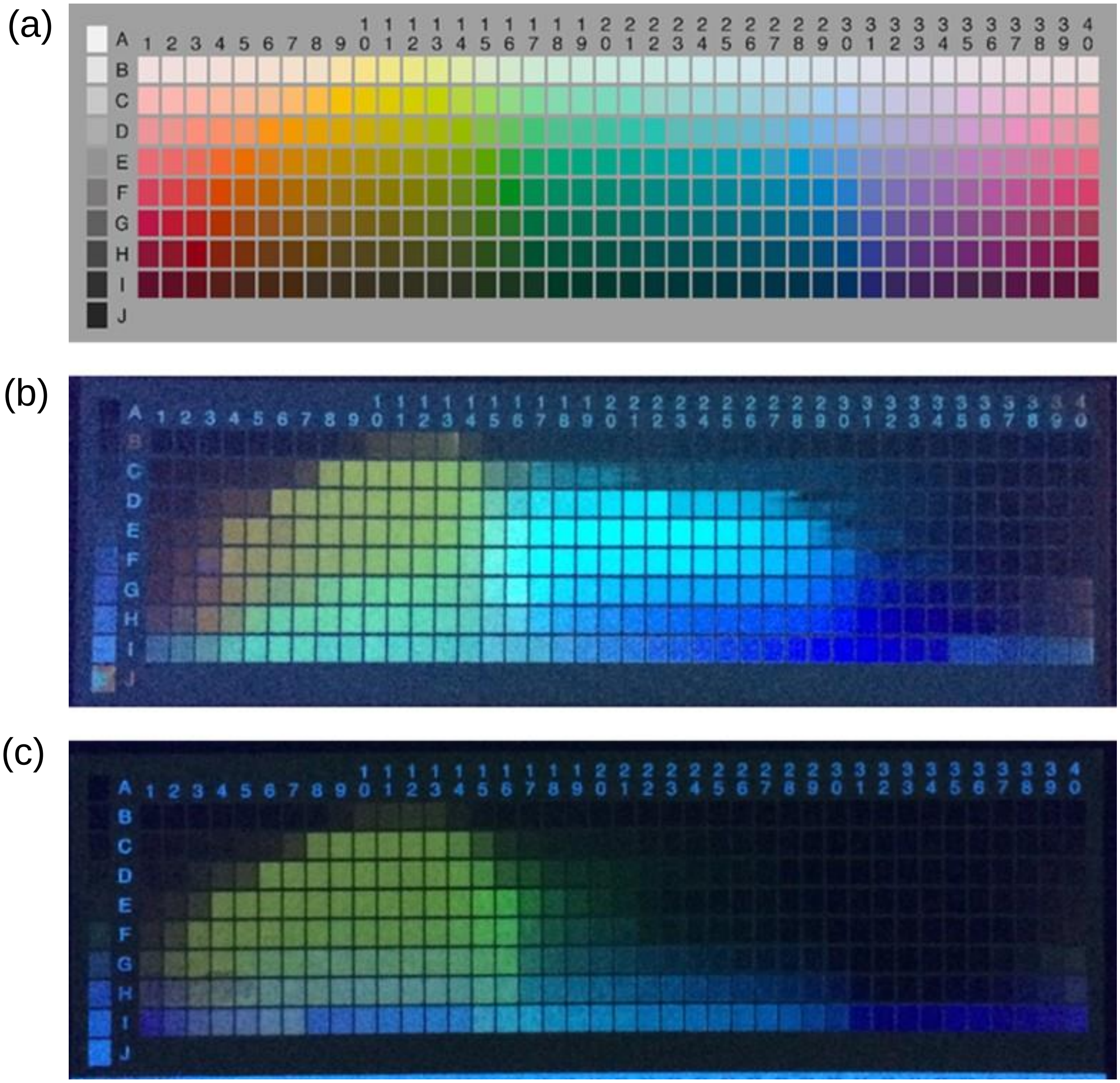

Supplementary Figure 75. (a) Digital Input files and the correspondent printed images under UV light (λ = 254 nm) using (b) the customised tri-colour inkjet cartridge and (c) the combination of a customised black and a tri-colour inkjet cartridge.

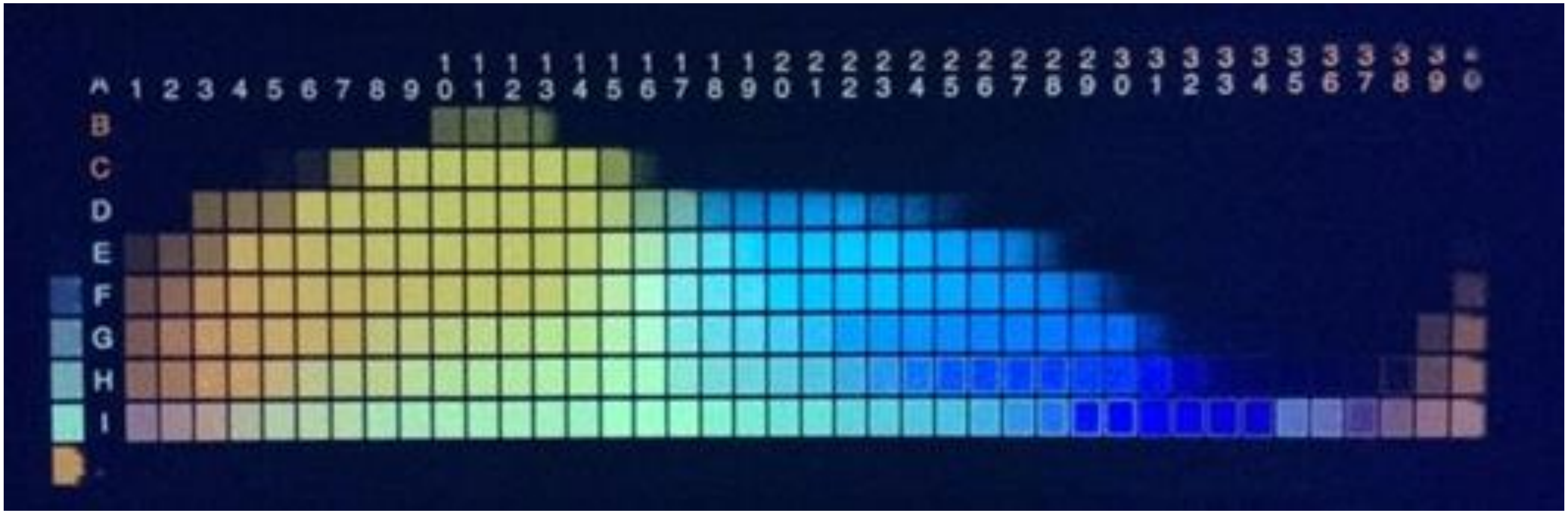

Supplementary Figure 76. Printed image under UV light (λ = 254 nm) using the customised tri-colour inkjet cartridge loaded with **R4**•4Cl/γ-CD (2 mL, **R4**•4Cl: 1 mM, γ-CD: 50 mM), AdMe•Cl (2 mL, 200 mM), and PyMe•Cl/γ-CD (2 mL, **PyMe**•Cl: 4 mM, γ-CD: 100 mM), in yellow, magenta, and cyan channels, respectively.

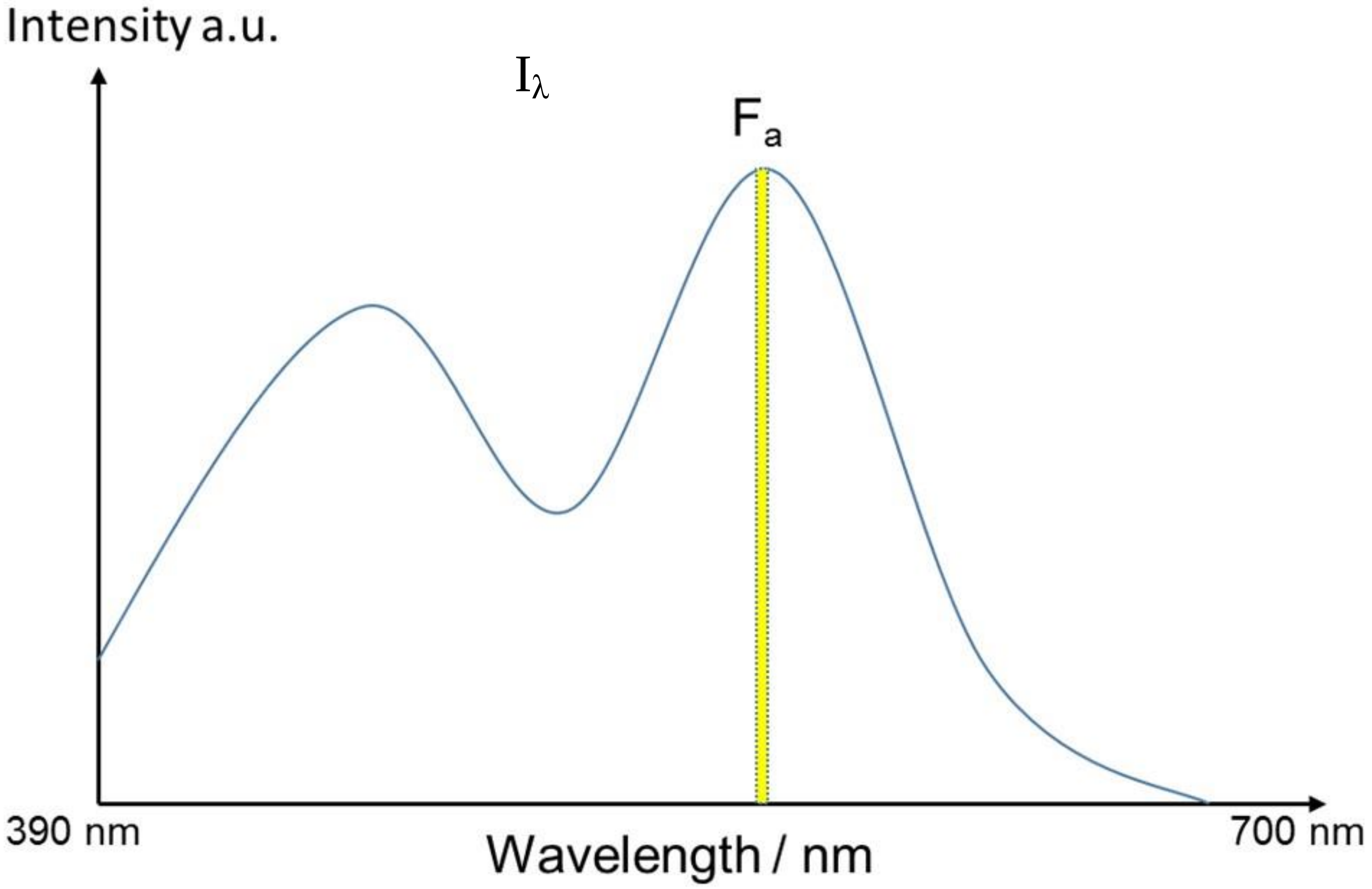

Supplementary Figure 77. Graphic illustration of the fluorescence emission of a printed dot at a particular wavelength.

Authentication Experiments

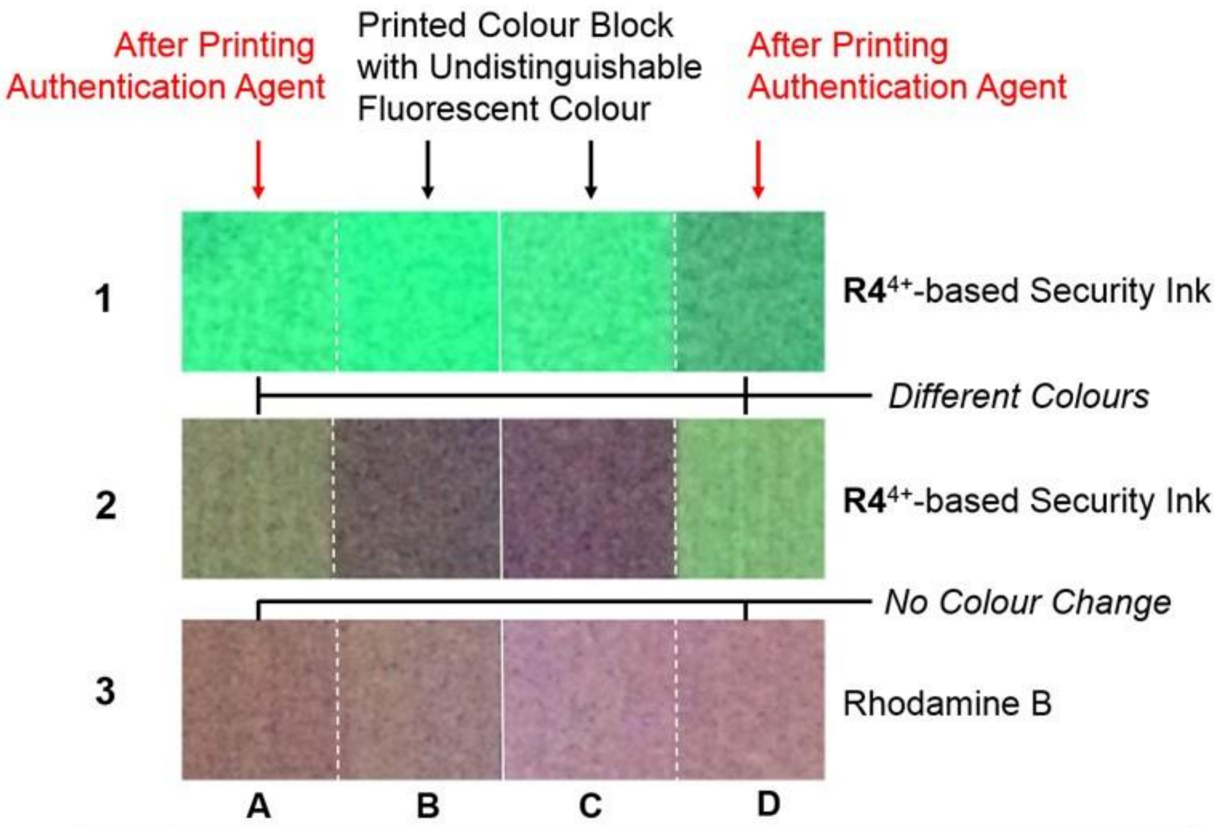

	A Authentication Agent	B	C	D Authentication Agent
		Ink Chemical Composition		
1	AdMe	$\mathbf{R4}^{4+}$ + 300 γ-CD	$\mathbf{R4}^{4+}$ + 800 γ-CD	AdMe
2	γ-CD	$\mathbf{R4}^{4+}$ + AdMe	$\mathbf{R4}^{4+}$	γ-CD
3	γ-CD	RhB + AdMe	RhB	γ-CD

Supplementary Figure 78. A demonstration of fluorescent colours produced by $\mathbf{R4}^{4+}$-based inks with different formulations that appear almost identical (cf. 1B and 1C, or 2B and 2C), but are distinguished (cf. 1A and 1D, or 2A and 2D) using an authentication agent. No distinguishable colour change is observed (line 3) using rhodamine B (RhB) as a reference compound.

Supplementary Tables

Supplementary Table 1. Yields of the hetero[3]rotaxane **R3**•4Cl and the hetero[4]rotaxane **R4**•4Cl synthesized from precursors **1**•Cl (2.2mM) and **2**•2Cl (1 mM), CB6 and γ-CD at various temperatures.

Entries	Temperature (^{o}C)	**1**•Cl (Equiv)	**2**•2Cl (Equiv)	CB6 (Equiv)	γ-CD (Equiv)	Yield / %	
						**R3**•4Cl	**R4**•4Cl
1	0	2.2	1.0	2.5	10	2 [a]	12 [a]
2	20	2.2	1.0	2.5	10	8 [a]	48 [a]
3	40	2.2	1.0	2.5	10	21 [a]	71 [a]
4	60	2.2	1.0	2.5	2.5	39 [b]	9 [b]
5	60	2.2	1.0	2.5	5	24[b]	21[b]
6	60	2.2	1.0	2.5	10	11 [a]	88 [a]
7	60	2.2	1.0	2.5	10	8 [b]	83 [b]
8	60	2.2	1.0	2.5	20	17 [b]	56 [b]
9	80	2.2	1.0	2.5	10	16 [a]	81 [a]

[a] Yields estimated by the integration of the corresponding peaks in the reverse-phase analytical HPLC traces. [b] Isolated yields.

Supplementary Table 2. Quantum yields of samples in this study in both aqueous solutions and solid state measured at room temperature.

	Quantum Yield	Φ = (%)
	Solution	Solid State
**1**•Cl	22.8 ± 0.25	4.0 ± 0.01
**2**•2Cl	51.3 ± 0.36	0.1
**R3**•4Cl	18.3 ± 0.11	1.7 ± 0.01
**R3**⊂CD complex 1:200		25.9 ± 0.04
**R4**•4Cl	52.4 ± 0.27	7.7 ± 0.01
**R4**⊂CD complex		
R4:CD =		
1:5		10.4 ± 0.02
1:20		12.1 ± 0.02
1:35		15.7 ± 0.03
1:50		19.6 ± 0.03
1:75		25.8 ± 0.03
1:100		31.0 ± 0.05
1:150		36.9 ± 0.06
1:200		45.7 ± 0.08
1:200 + 200 Ad•Cl		15.4 ± 0.03

Supplementary Table 3. Summary of writing tests using $\mathbf{R4^{4+}}\subset\gamma\text{-}CD_2$ ink on different media.

Writing media	Fluorescent colour under UV light
Copy paper (75 g/m^2, Office Depot)	Orange
Copy paper (75 g/m^2, Xerox)	Orange
Copy paper (80 g/m^2, HP)	Orange
Matte inkject print paper (120 g/m^2, HP)	Orange
Glossy inkject print paper (120 g/m^2, HP)	Orange
Newsprint paper (40 g/m^2)	Green
Rag paper (220 g/m^2, coated side)	Orange
Rag paper (220 g/m^2, uncoated side)	Green
Rag paper (320 g/m^2, coated side)	Orange
Rag paper (320 g/m^2, uncoated side)	Green
Filter paper (Whatman)	Green
Cigarette rolling paper	Green
Inkjet Photopaper (HP)	Green

As shown in Table S3, $\mathbf{R4^{4+}}\subset\gamma\text{-}CD_2$ ink is very sensitive to the paper media. Papers made of cellulose (rag paper and filter paper) as well as those made of plant fibers (newsprint and cigarette rolling paper) do not seems to interact with the $\mathbf{R4^{4+}}\subset\gamma\text{-}CD_2$ complex. Most of the daily-use copy papers contain a large number of additives, which include optical brightener, sizing agents, wet strength additive, dry strength additive, coating agents, etc. It is practically difficult to elucidate which additive(s) interacts with γ-CD, but this phenomenon has been observed consistently in all our writing tests. In addition, rag paper with a coated surface is able

to interact with γ-CD in the **R4**$^{4+}$⊂γ-CD$_2$ ink, resulting in a fluorescence colour change under UV light.

Supplementary Discussion

Supramolecular Encryption Theory

When mixing inks from each channel in the tri-colour inkjet cartridge, the colour of the printed dot is dependent to the amount of each ink deposited on the paper surface. In our studies in previous sections, we discovered that the behavior of the [4]rotaxane **R4**•4Cl in the presence of γ-CD or Ad•Cl in aqueous solution is very similar to its behavior in the solid state. Thus, we extend (Figure 2) the supramolecular equilibrium to illustrate the resulting ink colours on rag paper after printing.

In the tri-colour inkjet cartridge, the aqueous solutions of the [4]rotaxane **R4**•4Cl/γ-CD, γ-CD and Ad•Cl have been loaded separately into three channels. In our initial experiment, we chose Ad•Cl and AdMe•Cl as non-fluorescent competitive binding agent (**CBA**) to participate in the supramolecular equilibrium network. Thus, when the **CBA** is non-fluorescent, the aggregation equilibrium of the [4]rotaxane **R4**•4Cl in solution or in the solid state can be described using equation (6)

$$R4 \underset{}{\overset{K_{a1}}{\rightleftharpoons}} R4_2 \underset{}{\overset{K_{a2}}{\rightleftharpoons}} R4_3 \underset{}{\overset{K_{a3}}{\rightleftharpoons}} R4_4 \cdots \underset{}{\overset{K_{a(n-1)}}{\rightleftharpoons}} R4_n \tag{6}$$

If the aggregation of the [4]rotaxane **R4**•4Cl is homogenous and there is no cooperativity during the nucleation process, the aggregation process can be simplified as

$$nR4 \underset{}{\overset{n^n K_{agg}{}^{n-1}}{\rightleftharpoons}} R4_n \tag{7}$$

Assuming the extents of aggregation in each step are identical, and so

$$K_{a1} = K_{a2} = K_{a3} = \cdots = K_{a(n-1)} \tag{8}$$

The process can be simplified as

$$2R4 \underset{}{\overset{4K_{agg}}{\rightleftharpoons}} R4_2 \tag{9}$$

The aggregation constant $K_{agg} = \frac{[R4_2]}{4[R4]^2}$ (10)

Adding γ-CD to the [4]rotaxane **R4**•4Cl affords a stepwise binding event, where the γ-CD and **R4**•4Cl forms 1:1 and 2:1 complexes sequentially.

$$R4 + CD \underset{}{\overset{K_1}{\rightleftharpoons}} R4 \subset CD + CD \underset{}{\overset{K_2}{\rightleftharpoons}} R4 \subset CD_2 \tag{11}$$

where $K_1 \approx K_2$, or

$$R4 + 2CD \underset{}{\overset{K_{CD}^2}{\rightleftharpoons}} R4 \subset CD_2 \tag{12}$$

where $K_{\mathrm{CD}}^{2} = K_1 \bullet K_2$ (13)

Upon the addition of Ad•Cl or AdMe•Cl, the encapsulation of the competitive binding agent (**CBA**) by γ-CD can be described as

$$\mathrm{CBA} + \mathrm{CD} \xrightleftharpoons{K_{\mathrm{CBA}}} \mathrm{CBA} \subset \mathrm{CD} \quad (14)$$

and the binding constant $K_{\mathrm{CBA}} = \dfrac{[\mathrm{CBA} \subset \mathrm{CD}]}{[\mathrm{CBA}] \bullet [\mathrm{CD}]}$ (15)

At any particular wavelength, the fluorescent emission *F* can be described as

$$F = F_{\mathrm{R4}} + F_{\mathrm{R4 \subset CD}} + F_{\mathrm{R4 \subset CD_2}} + F_{\mathrm{R4_2}} + F_{\mathrm{CBA}} + F_{\mathrm{CD}} + F_{\mathrm{CBA \subset CD}} \quad (16)$$

The overall fluorescence colour output of a printed dot is determined by the fluorescence emission of the inks at every wavelength in the visible spectrum (390 ─ 700 nm).

If the CBA is not fluorescent

The fluorescence intensity I_λ at a given wavelength λ is the sum of the emission intensities of each component, which is given by

$$I_\lambda = F_{\mathrm{R4}} + F_{\mathrm{R4_2}} + F_{\mathrm{R4 \subset CD}} + F_{\mathrm{R4 \subset CD_2}} \quad (17)$$

Then

$$I_\lambda = F^\circ_{\mathrm{R4}}[\mathrm{R4}] + F^\circ_{\mathrm{R4_2}}[\mathrm{R4_2}] + F^\circ_{\mathrm{R4 \subset CD}}[\mathrm{R4} \subset \mathrm{CD}] + F^\circ_{\mathrm{R4 \subset CD_2}}[\mathrm{R4} \subset \mathrm{CD_2}] \quad (18)$$

where $F°_{R4}$, $F°_{R4_2}$, $F°_{R4\subset CD}$ and $F°_{R4\subset CD_2}$ are the molar fluorescence coefficients of each species.

Combining equations (9) − (14) and (18) results in,

$$I_\lambda = (F°_{R4} + F°_{R4\subset CD} \bullet K_{CD}[CD] + F°_{R4\subset CD_2} \bullet K_{CD}^2 \bullet [CD]^2) \bullet [R4] + F°_{R4_2} \bullet 4K_{agg} \bullet [R4]^2$$

(19)

Combining *Eqs. C*, *E* and *F* affords the overall equilibrium network as

$$3R4 + 3CD + CBA \xrightleftharpoons{4K_{agg} \bullet K_{CD}^2 \bullet K_{CBA}} R4_2 + R4 \subset CD_2 + CBA \subset CD \qquad (20)$$

Assuming *m* amount of **CBA** has been consumed in the reaction

Then, $$4K_{agg} \bullet K_{CD}^2 \bullet K_{CBA} = \frac{m^3}{([R4]_0 - 3m)^3([CD]_0 - 3m)^3([CBA]_0 - m)} \qquad (21)$$

Since $[R4] = [R4]_0 - 3m$ (22), and $[CD] = [CD]_0 - 3m$ (23),

Then,

$$I_\lambda = F°_{R4} + F°_{R4\subset CD} \bullet K_{CD}([CD]_0 - 3m) + F°_{R4\subset CD_2} \bullet K_{CD}^2 \bullet ([CD]_0 - 3m)^2 \bullet ([R4]_0 - 3m) + F°_{R4_2} \bullet 4K_{agg} \bullet ([R4]_0 - 3m)^2 \qquad (24)$$

If the CBA is fluorescent

The fluorescence intensity I_λ at a given wavelength λ can be described as

$$I_\lambda = F^\circ{}_{\mathrm{R4}} \bullet [\mathrm{R4}] + F^\circ{}_{\mathrm{R4_2}} \bullet [\mathrm{R4_2}]$$
$$+ F^\circ{}_{\mathrm{R4 \subset CD}} \bullet [\mathrm{R4 \subset CD}] + F^\circ{}_{\mathrm{R4 \subset CD_2}} \bullet [\mathrm{R4 \subset CD_2}]$$
$$+ F^\circ{}_{\mathrm{CBA}} \bullet [\mathrm{CBA}] + F^\circ{}_{\mathrm{CBA \subset CD}} \bullet [\mathrm{CBA \subset CD}] \qquad (25)$$

Because of equations (22) and (23),

and $[\mathrm{CBA}] = [\mathrm{CBA}]_0 - m$ (26), therefrore

$$I_\lambda = F^\circ{}_{\mathrm{R4}} + F^\circ{}_{\mathrm{R4 \subset CD}} \bullet K_{\mathrm{CD}} ([\mathrm{CD}]_0 - 3m)$$
$$+ F^\circ{}_{\mathrm{R4 \subset CD_2}} \bullet K_{\mathrm{CD}}^2 \bullet ([\mathrm{CD}]_0 - 3m)^2 \bullet ([\mathrm{R4}]_0 - 3m)$$
$$+ F^\circ{}_{\mathrm{R4_2}} \bullet 4K_{\mathrm{agg}} \bullet ([\mathrm{R4}]_0 - 3m)^2$$
$$+ F^\circ{}_{\mathrm{CBA}} \bullet ([\mathrm{CBA}]_0 - m) + F^\circ{}_{\mathrm{CBA \subset CD}} \bullet m \qquad (26)$$

Authentication Experiments

Theory: Anti-counterfeiting technology and counterfeiting is a continuous battle. The [4]rotaxane-based security ink provides several ‘keys’ to encrypt the printed image by means of polychromic printing. By employing these keys, which are (i) the initial concentrations of inks, (ii) the binding constants, and (iii) the choice of the **CBA**s, one can easily manipulate (iv) the fluorescent colour distribution, (v) the colour spectrum width and (vi) the colour intensity of the output file. These security features embody the first level security of the [4]rotaxane-based security ink.

Benefiting from the rich properties of the [4]rotaxane, we can further add another level of security through a post-printing authentication process. Post-printing a layer of authentication reagent on top of a printed polychromic image results in further colour changes. Even if a counterfeiter could reverse-engineer to match the fluorescence colour of the original printed polychromic image, reproducing a colour-change process of the polychromic fluorescent image is nigh impossible. Firstly, the colour changing process is dynamic – depending on the amount of authentication reagents applied — i.e., printed or sprayed onto the image — the final colour distribution will be different. Secondly, there are more than 10 post-print authentication reagents that can be applied to give fingerprint-like colour changes. They can be used either individually or combined together to verify the suspect product. This method, therefore, creates a large number of colour changing combinations, which can be used to verify the authenticity of the document.

Methods. Aqueous solutions of post-printing reagents were loaded into empty black inkjet cartridges and test rag paper with a previously printed colour palette was loaded into the paper tray of an inkjet printer. A homogenous layer of the ink was printed across the sheet of rag paper in order to coat the colour palette. Images were taken under UV light (λ = 254) in a dark room.

Strategies. Post-print authentication can be carried out using four different strategies, namely, (i) fluorescent colour changing using authentication reagents that are themselves non-fluorescent,

(ii) the introduction of new colours using fluorescent authentication reagents, (iii) addition of counterion exchange reagents, and (iv) washing with water.

- *(i) Fluorescence colour change using non-fluorescent authentication reagents*. Post-printing solutions of binding reagents (e.g., γ-CD) or **CBA**s (Ad•Cl, AdMe•Cl and etc) on top of images results in a characteristic colour change. For example, post-printing a γ-CD solution on top of an existing multi-chromic print will cause hypsochromic shifts in the fluorescene emission colours of areas that were initially red, towards more green emission, without affecting colours that were initially green or blue. Post-printing AdMe•Cl solution will affect several colour regions. The solutions of the authentication reagents are transparent and visibly undistinguishable from one another. When performing the authentication test, a consumer could easily use a spray bottle (for example, the bottles used to dispense perfumes) to spray the authentication reagents on top of the printed information and verify the colour change.

- *(ii) Fluorescence colour change by printing fluorescent authentication reagents*. Instead of using non-fluorescent compounds to perform the authentication test, many water soluble fluorescent dyes can also be used to verify the authentication of a given document. These fluorescent dyes not only act as a **CBA** to shift the supramolecular equilibrium, but they also contribute to the colour intensity and colour spectrum width.

- *(iii) Fluorescence colour changing by counterion exchanging reagents*. Exchanging the chloride counterions of the [4]rotaxane by the addition of fluorescent or non-fluorescent salts changes the solid-state fluorescence of the printed image. Unlike strategies (i) and (ii), after the counterion exchange process, the [4]rotaxane ink is nearly insoluble in water, thus fixing the colour change.

- *(iv) Fluorescence colour changing by water washing*. The simplest way to assess the authenticity of a printed image is by rinsing with water. Both the binding reagents (e.g. γ-CD) and the **CBA**s (Ad•Cl, AdMe•Cl and etc) are water soluble, so can be removed easily by washing. Surprisingly, although the [4]rotaxane ink is water soluble when first prepared, it stains paper media very strongly and is resistant to aqueous washing. After soaking the printed documents in water over 24 h, most of the fluorescence colour is lost, but the orange emission from [4]rotaxane still remains.

Supplementary Methods

Materials and Methods

All reagents were purchase from commercial suppliers (Aldrich, Fisher and Wacker) and used as received. Thin layer chromatography (TLC) was performed on silica gel 60 F254 (E. Merck).

Column chromatography was carried out on silica gel 60F (Merck 9385, 0.040–0.063 mm). UV/Vis Absorption spectra were measured on a Shimadzu 3600 UV/Vis/NIR spectrometer with a temperature control system, employing cuvettes with path lengths of 1 and 10 mm. Steady-state fluorescence spectra of liquid samples were measured on a Horiba FluoroMax-4 spectrofluorometer. Steady-state fluorescence spectra of solid samples were measured on an ISS PC1 Fluorimeter. Absolute fluorescence quantum yields of were measured on a Horiba FluoroMax-4 spectrofluorometer with a Quanta-φ integrating sphere attachment, and the resulting data was analyzed by the 4-curve method using the FluorEssence 3.5 software. Circular dichroism spectra were measured on a Jasco J-815 Circular Dichoism spectrometer with a temperature control system. Dynamic light scattering data was obtained on a Malvern Zetasizer Nano with a 633 nm He-Ne laser light source. Nuclear magnetic resonance (NMR) spectra were recorded on Bruker Avance 500 or 600 spectrometers with working frequencies of 500 or 600 MHz for ^{1}H and 125 or 150 MHz for ^{13}C nuclei, respectively. Chemical shifts are reported in ppm relative to the signals corresponding to the residual non-deuterated solvents ($CDCl_3$: δ = 7.26 ppm, CD_3CN: δ = 1.94 ppm, D_2O: δ = 4.79 ppm and $(CD_3)_2SO$: δ = 2.50 ppm). Low-resolution electrospray ionization mass spectra (ESI-MS) were recorded on either an Agilent 1100 MSD or a Thermo Finnegan LCQ instrument. High-resolution electrospray ionization mass spectra (HR-ESI-MS) were recorded on an Agilent 6210 LC-TOF instrument with Agilent 1200 HPLC introduction. Writing tests were performed using Pilot Parallel pens, which were filled with customised inks cartridges. Printing tests were performed using a HP Photosmart CP4780 inkjet printer with HP60 black and tricolour ink cartridges, which were filled with customised inks.

Synthetic Methods

Synthesis of 1•Cl

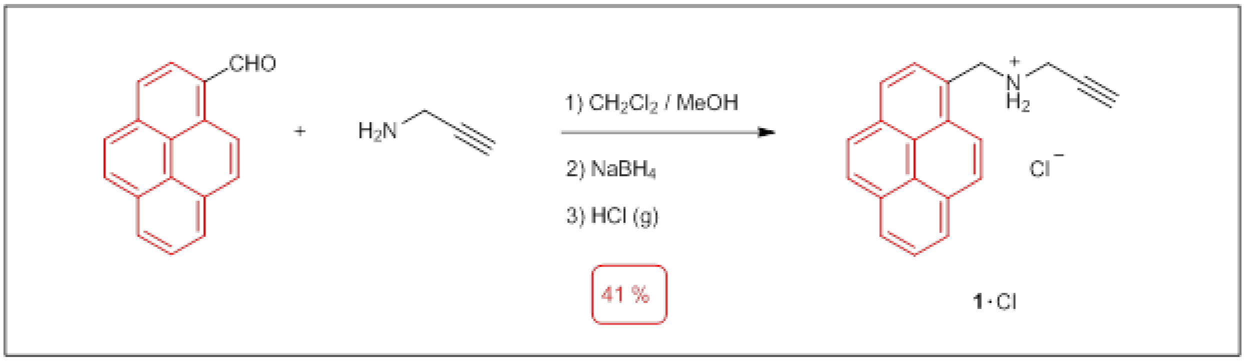

1•Cl[2]: 1-Pyrenecarboxaldehyde (2.00 g, 8.69 mmol) was dissolved in CH_2Cl_2/MeOH (v/v, 4:1, 50 mL) and propargylamine (0.62 mL, 9.6 mmol) was added to the solution. The reaction mixture was heated under reflux in an atmosphere of nitrogen for 18 h. After all the 1-pyrene-carboxaldehyde had been consumed (monitored by TLC), the reaction mixture was cooled to 0 °C then $NaBH_4$ (1.50 g) was added in portions to the reaction mixture. After stirring for 6 h, an aqueous HCl solution (37% w/w, 6 mL) was added to quench the excess of $NaBH_4$ in the reaction mixture. The solvent was removed under the reduced pressure and then an aqueous 2 M NaOH solution (50 mL) was added. The reaction mixture was extracted with CH_2Cl_2 (3 × 100 mL) and the organic phase was dried (Na_2SO_4). The CH_2Cl_2 solution was purged with an excess of HCl gas (HCl gas was generated by adding saturated aqueous HCl solution to anhydrous $CaCl_2$), whereupon precipitation occurred. The precipitate was collected by filtration and washed with an excess of CH_2Cl_2 to afford **1**•Cl as an light yellow powder (1.10 g, 41 %). ^{1}H NMR (500 MHz, D_2O, 298 K): δ = 8.18 (d, J = 7.6 Hz, 1H), 8.13 (d, J = 7.6 Hz, 1H), 8.02 (t, J = 7.7 Hz, 1H), 8.00 (d, J = 8.8 Hz, 1H), 7.89 (d, J = 7.6 Hz, 1H), 7.89 (d, J = 10.0 Hz, 1H), 7.86 (d, J = 8.9 Hz, 1H), 7.73 (d, J = 9.2 Hz, 1H), 7.69 (d, J = 7.9 Hz, 1H), 4.60 (s, 2H), 3.89 (d, J = 2.6 Hz, 2H), 3.07 (t, J = 2.5 Hz, 1H). ^{13}C NMR (125 MHz, D_2O, 298 K): δ = 131.7, 130.5, 129.8, 128.6, 128.5, 128.4, 128.1, 127.0, 126.5, 125.8, 125.7, 124.6, 123.4, 123.1, 122.3, 121.0, 78.4, 73.1, 46.8, 35.9. ESI-MS: calcd for $[M - Cl]^+$ m/z = 270.1, found m/z = 270.0.

Synthesis of **2**•2Cl

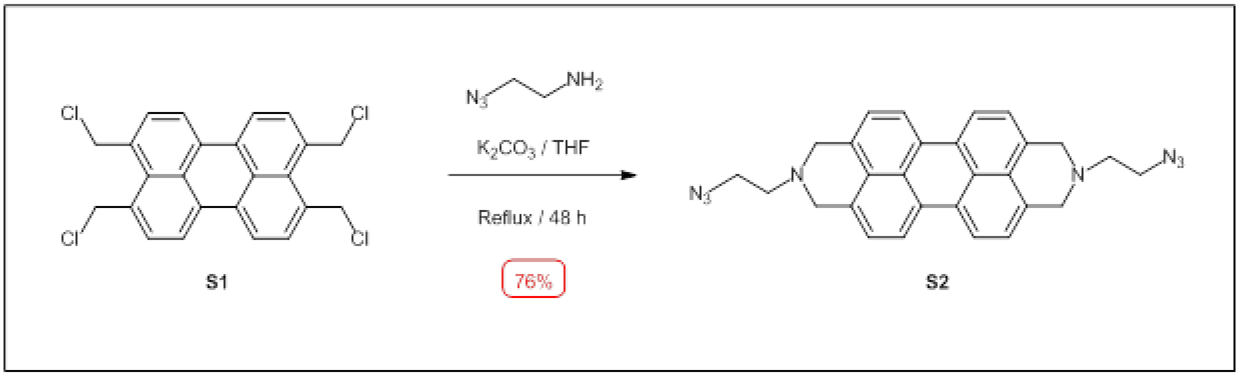

S2: Compound **S2** was synthesized using a protocol based on a previously reported procedure[3] with slight modifications. Compound **S1** (0.50 g, 1.12 mmol), 2-azidoethylamine[3] (1.46 g, 17.0 mmol) and K_2CO_3 (1.00 g, 7.24 mmol, anhydrous) in anhydrous THF (50 mL) was heated under reflux in an atmosphere of nitrogen for 48 h. The solvent was then removed under reduced pressure and the residue was purified by column chromatography (SiO_2, gradient CH_2Cl_2 — 10% Me_2CO in CH_2Cl_2 (m/v), R_f = 0.4) to afford the product **S2** as an orange-yellow powder (402 mg, 76%). *Caution: In the previously reported procedure,*[4] *simple washing by H_2O and Et_2O is not sufficient to remove all the byproducts of the reaction. The product* ***S2****, purified after column chromatography, is highly soluble in common organic solvents.* ^1H NMR (500 MHz, $CDCl_3$, 298 K): δ = 8.08 (d, J = 7.6 Hz, 4H), 7.22 (d, J = 7.6 Hz, 4H), 4.05 (s, 8H), 3.57 (t, J = 6.2 Hz, 4H), 2.87 (t, J = 6.2 Hz, 4H). ^{13}C NMR (125 MHz, $CDCl_3$, 298 K): δ = 132.1, 130.2, 129.3, 128.4, 123.5, 119.6, 56.9, 55.6, 49.3. ESI-MS: calcd for $[M + H]^+$ m/z = 473.2, found m/z = 473.2.

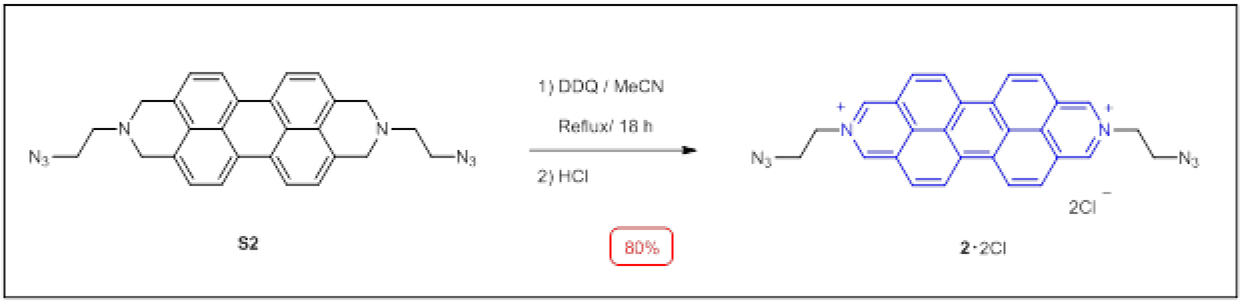

2•2Cl: The diazide **2**•2Cl was synthesized based on a previously reported method[5] with slight modifications. Compound **S2** (0.50 g, 1.06 mmol) and 2,3-dichloro-5,6-dicyano-1,4-benzo-quinone (DDQ) in anhydrous degassed MeCN (80 mL) was heated under reflux in an atmosphere of nitrogen for 18 h. The reaction mixture was cooled to room temperature, followed by adding 6 mL of aqueous HCl solution (37% w/w), which generated a precipitate. This precipitate was collected by filtration and washed with 1:1 Et_2O/MeCN (4 × 50 mL) to afford a crude product as a dichloride salt. The impurities in the crude product were removed effectively by initiating two cycles of a counterion exchange procedure. Firstly, an excess of NH_4PF_6 was added to the aqueous solution of the crude product in order to exchange the counterion from Cl^- to PF_6^- and generate **2**•$2PF_6$ as an orange precipitate. The precipitate was collected by filtration and washed with an excess of H_2O. The second counterion exchange process involved adding a large excess of tetrabutylammonium chloride (TBACl) to a MeCN solution of **2**•$2PF_6$. The precipitate which formed was collected by filtration and washed with an excess of MeCN to afford pure **2**•2Cl as a dark yellow powder (455 mg, 80%). ^{1}H NMR (500 MHz, $(CD_3)_2SO$, 298 K): δ = 10.39 (s, 4H), 10.07 (d, *J* = 9.4 Hz, 4H), 9.05 (d, *J* = 9.2 Hz, 4H), 5.37 (t, *J* = 5.5 Hz, 4H), 4.37 (t, *J* = 5.3 Hz, 4H). ^{13}C NMR (125 MHz, $(CD_3)_2SO$, 298 K): δ = 139.8, 128.8, 128.6, 127.5, 127.0, 121.3, 61.0, 50.8. ESI-MS: calcd for $[M - Cl]^+$ *m/z* = 530.2, found *m/z* = 530.2.

Synthesis of [3]Rotaxane **R3**$^{4+}$ and [4]Rotaxane **R4**$^{4+}$

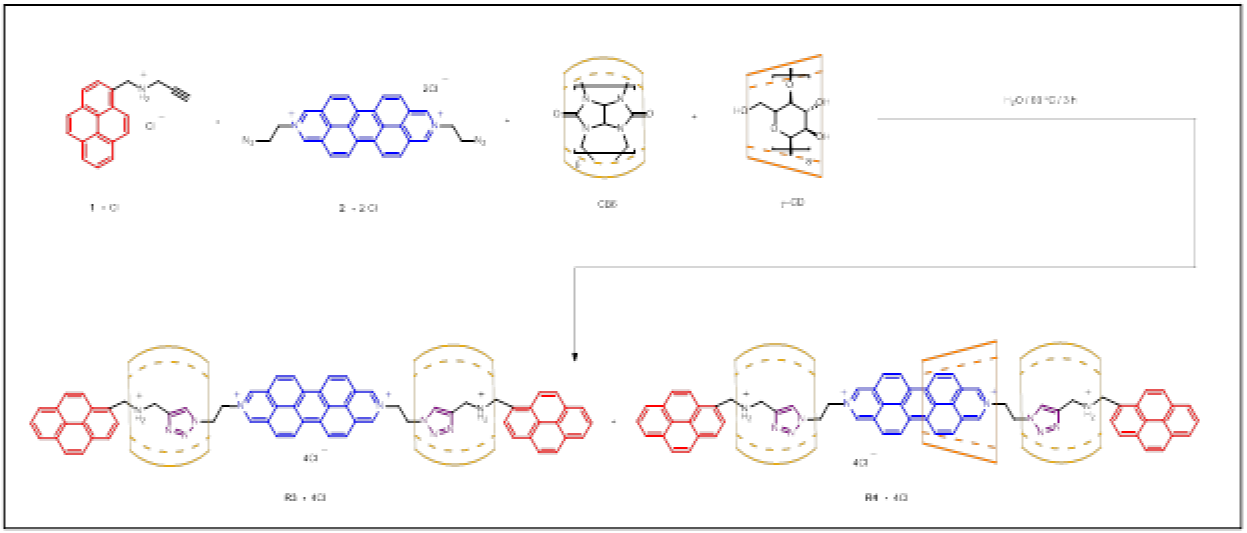

General procedure for the synthesis of [3]rotaxane R3^{4+} **and the [4]rotaxane R4**$^{4+}$. The stopper precursor **1**•Cl (2.2 equiv), the dumbbell precursor **2**•2Cl (1.0 equiv), and γ-CD (2.5 – 20 equiv) were mixed in H_2O and stirred at 60 °C for 10 min before CB6 (2.5 equiv) was added. The reaction mixture was stirred at 60 °C for a further 18 h. Afterwards, insoluble residues were filtered off from the reaction mixture and an excess of NH_4PF_6 was added to the filtrate. The precipitate which formed was collected by filtration and washed with an excess of H_2O. This mixture of products was dissolved in MeCN and subjected to reverse-phase analytical HPLC analysis (C18 column, 5 μm packing, length 150 mm, diameter 4.6 mm, eluent: 0 min 0.1% TFA in H_2O 99.9% to 40 min 0.1% TFA in 99.9% MeCN, flow rate: 1 mL/min). An excess of Bu_4NCl was added to this MeCN solution to ensure that all the counterions are exchanged to Cl^- ions. The precipitate which formed was collected, washed with an excess of MeCN and purified on a reverse-phase C18 column (150 gram, RediSep Rf Gold C18Aq) on an automatic column chromatography system (Combiflash Rf200, Teledyne Isco, eluent: H_2O / MeCN (0.1 % TFA) = 100 / 0 to 0 / 60 in 40 mins, flow rate: 85 mL/min) to afford **R3**$^{4+}$ and **R4**$^{4+}$as their TFA salts.

The appropriate fractions containing **R3**$^{4+}$ were collected and the counterion of the **R3**$^{4+}$ was exchanged first of all to PF_6^- on addition of an excess of NH_4PF_6 to the collected fractions. After removal of MeCN under reduced pressure, the product **R3**•4PF_6 (3 − 41%) precipitated out from the solution and it was collected by vacuum filtration with extensive washing with H_2O.

R3•4PF_6: ^{1}H NMR (500 MHz, $(CD_3)_2SO$, 273 K): δ = 10.84 (s, 4H), 10.15 (d, *J* = 8.8 Hz, 4H), 9.04 (d, *J* = 8.8 Hz, 4H), 8.84 (d, *J* = 9.3 Hz, 2H), 8.48 – 8.23 (m, 18H), 8.14 (t, *J* = 7.6 Hz, 2H), 6.62 (s, 2H), 5.75 (t, *J* = 8.5 Hz, 4H), 5.65 (d, *J* = 14.8 Hz, 12H), 5.51 (d, *J* = 14.8 Hz, 12H), 5.42 (s, 24H), 5.08 (t, *J* = 6.0 Hz, 4H), 4.72 (d, *J* = 6.0 Hz, 4H), 4.39 (t, *J* = 8.5 Hz, 4H), 4.33 (d, *J* = 14.9 Hz, 12H), 4.27 (d, *J* = 14.9 Hz, 12H). On account of the poor solubility of **R3**•4PF_6, the signals for the ^{13}C NMR spectrum of **R3**•4PF_6 in $(CD_3)_2SO$ were too weak to be assigned. HR-ESI-MS: calcd for $[M – 3PF_6]^{4+}$ *m/z* = 1048.9971, found *m/z* = 1048.9984; $[M – 2PF_6]^{3+}$ *m/z* = 1645.9780, found *m/z* = 1645.9751.

The [3]rotaxane **R3**•4Cl was obtained after a second counterion exchange procedure wherein an excess of Bu_4NCl was added to a MeCN solution of **R3•**4PF_6, resulting in the formation of a precipitate, which was collected by vacuum filtration, washed with excess of MeCN and dried under vacuum to afford **R3**•4Cl (2 − 39%) as an orange powder.

R3•4Cl: ^{1}H NMR (600 MHz, D_2O, 343 K): δ = 10.61 (s, 4H), 9.86 (d, *J* = 8.2 Hz, 4H), 9.07 (d, *J* = 8.2 Hz, 4H), 8.93 (d, *J* = 9.1 Hz, 2H), 8.62 (d, *J* = 7.8 Hz, 2H), 8.51 – 8.38 (m, 8H), 8.31 (q, *J* = 8.6, 7.6 Hz, 4H), 8.19 (t, *J* = 7.7 Hz, 2H), 6.77 (s, 2H), 5.86 (d, *J* = 15.4 Hz, 16H), 5.72 (d, *J* = 15.4 Hz, 12H), 5.50 (s, 24H), 5.35 (s, 4H), 4.74 (s, 4H), 4.66 (t, *J* = 9.3 Hz, 4H), 4.26* (d, *J* = 15.4 Hz, 12H), 4.26* (d, *J* = 15.4, 12H). (* These signals were obscured by the solvent residue peak at 4.26 ppm). On account of the poor solubility of **R3**•4Cl, the signals for the ^{13}C NMR spectrum of **R3**•4Cl in D_2O were too weak to be assigned. HR-ESI-MS: calcd for $[M – 4Cl]^{4+}$

m/z = 750.5067, found *m/z* = 750.5081; [*M* – H – 4Cl]$^{3+}$ *m/z* = 1000.3398, found *m/z* = 1000.3422; [*M* – 3Cl]$^{3+}$ *m/z* = 1012.6657, found *m/z* = 1012.6674.

Optimized synthesis of the hetero[4]rotaxane R4•4Cl. The stopper precursor **1**•Cl (67 mg, 0.22 mmol), the dumbbell precursor **2**•2Cl (54 mg, 0.10 mmol), and γ-CD (1287 mg, 1.00 mmol) were mixed together in H_2O (35 mL) and stirred at 60 °C for 10 min before CB6 (250 mg, 0.25 mmol) was added. The reaction mixture was stirred for a further 3 h at 60 °C. Thereafter, the insoluble residues were filtered off from the reaction mixture. The filtrate was loaded directly onto a reverse-phase C18 column (150 gram, RediSep Rf Gold C18Aq) on an automatic column chromatography system (Combiflash Rf200, Teledyne Isco) (eluent: H_2O / MeCN (0.1 % TFA) = 100 / 0 to 0 / 60 in 40 mins, flow rate: 85 mL/min). The appropriate fractions containing **R4**$^{4+}$ were collected and the counterion of the **R4**$^{4+}$ was exchanged to PF_6^- on addition an excess of NH_4PF_6 to the collected fractions. After removal of MeCN under reduced pressure, the product **R4**•4PF_6 (416.4 mg, 85%) precipitated out from the solution: it was collected by vacuum filtration with extensive washing with H_2O.

R4•4PF_6: ^{1}H NMR (500 MHz, CD_3CN, 298 K): δ = 11.02 (s, 2H), 10.86 (s, 2H), 10.07 (d, 2H, *J* = 9.1 Hz), 10.06 (d, *J* = 9.4 Hz, 2H), 9.35 (d, *J* = 9.1 Hz, 2H), 8.99 (d, *J* = 9.4 Hz, 2H), 8.87 (d, *J* = 9.3 Hz, 2H), 8.48 (dd, *J* = 7.8, 4.4 Hz, 2H), 8.43 – 8.26 (m, 12H), 8.22 (q, *J* = 8.9 Hz, 4H), 8.12 (t, *J* = 7.6 Hz, 2H), 6.68 (s, 1H), 6.67 (s, 1H), 6.17 – 5.97 (m, 2H), 5.91 (d, *J* = 15.4 Hz, 6H), 5.84 – 5.88 (m, 2H), 5.81 (d, *J* = 15.2 Hz, 6H), 5.69 (d, *J* = 15.1 Hz, 12H), 5.34 (s, 12H), 5.28 (s, 12H), 5.14 (d, *J* = 6.4 Hz, 2H), 5.13 (d, *J* = 6.4 Hz, 2H), 5.07 (d, *J* = 2.7 Hz, 8H), 4.83 (d, *J* = 3.7 Hz, 8H), 4.78 (d, *J* = 9.7 Hz, 2H), 4.77 (d, *J* = 9.4 Hz, 2H), 4.62 (d, *J* = 6.7 Hz, 8H), 4.47 – 4.40 (m, 2H), 4.38 – 4.24 (m, 2H), 4.20 (d, *J* = 15.3 Hz, 6H), 4.11 (d, *J* = 15.3 Hz, 6H), 4.06 (d, *J* = 15.4ß Hz, 6H), 3.63 (t, *J* = 9.7 Hz, 8H), 3.50 – 3.31 (m, 32H), 3.27 (t, *J* = 9.3 Hz,

8H), 2.81 (t, J = 5.3 Hz, 8H). ^{13}C NMR (125 MHz, CD_3CN, 298 K): δ = 156.9, 156.6, 156.5, 156.4, 142.5, 142.2, 140.5, 133.1, 132.1, 131.6, 131.5, 131.4, 129.6, 129.4, 129.2, 128.7, 128.5, 128.3, 127.9, 127.4, 126.6, 126.5, 126.1, 126.0, 125.6, 125.3, 125.0, 120.1, 103.5, 82.9, 74.1, 73.6, 70.8, 70.7, 61.6, 58.8, 52.4, 52.2, 52.1, 52.0, 51.1, 51.0, 50.0, 49.5, 44.9, 44.7. HR-ESI-MS: calcd for $[M - 4PF_6]^{4+}$ m/z = 1074.8634, found m/z = 1074.8631; $[M - 3PF_6]^{3+}$ m/z = 1481.4728, found m/z = 1481.4707.

The [4]rotaxane **R4**•4Cl was obtained after a second counterion exchange procedure wherein an excess of Bu_4NCl was added to a MeCN solution of **R4•**$4PF_6$, resulting in the formation of a precipitate, which was collected by vacuum filtration, washed with excess of MeCN and dried under vacuum to afford **R4**•4Cl (371 mg, 83 %) as an orange powder.

R4•4Cl: ^{1}H NMR (600 MHz, D_2O, 353 K): δ = 10.92 (s, 2H), 10.63 (s, 2H), 9.73 (d, J = 9.5 Hz, 2H), 9.68 (d, J = 9.4 Hz, 2H), 9.11 (d, J = 9.3 Hz, 2H), 9.05 (d, J = 9.3 Hz, 2H), 8.81 (dd, J = 12.4, 9.3 Hz, 2H), 8.50 (dd, J = 11.5, 7.9 Hz, 2H), 8.40 – 8.22 (m, 8H), 8.18 (d, J = 8.9 Hz, 4H), 8.08 – 7.98 (m, 2H), 6.67 (s, 1H), 6.63 (s, 1H), 5.96 (t, J = 8.8 Hz, 2H), 5.86 (d, J = 15.3 Hz, 6H), 5.80 (m, 2H), 5.76 (d, J = 15.5 Hz, 6H), 5.62 (d, J = 15.5, 6H), 5.60 (d, J = 15.5, 6H), 5.40 (s, 12H), 5.39 (s, 12H), 5.26 (s, 4H), 4.86 (d, J = 3.8 Hz, 8H), 4.66 (s, 4H), 4.53 (t, J = 9.0 Hz, 2H), 4.29 (t, J = 8.9 Hz, 2H), 4.10 (m, 24H), 3.65 (t, J = 9.5 Hz, 8H), 3.50 (dd, J = 10.0, 3.8 Hz, 8H), 3.41 – 3.20 (m, 32H). ^{13}C NMR (150 MHz, D_2O, 353 K): δ = 156.9, 156.8, 156.6, 140.1, 140.0, 130.9, 128.3, 128.1, 127.8, 127.6, 125.6, 125.3, 124.5, 120.3, 109.2, 103.1, 82.3, 81.4, 73.8, 73.5, 72.9, 72.8, 70.8, 70.7, 60.4, 58.2, 57.8, 52.4, 52.3, 52.0, 51.9, 50.3, 48.8, 44.1. HR-ESI-MS: calcd for $[M - 4Cl]^{4+}$ m/z = 1074.8630, found m/z = 1074.8623; $[M - H - 4Cl]^{3+}$ m/z = 1432.8149, found m/z = 1432.8123; $[M - 3Cl]^{3+}$ m/z = 1445.1406, found m/z = 1445.1369.

Synthesis of [4]Rotaxane **SR4**•4Cl

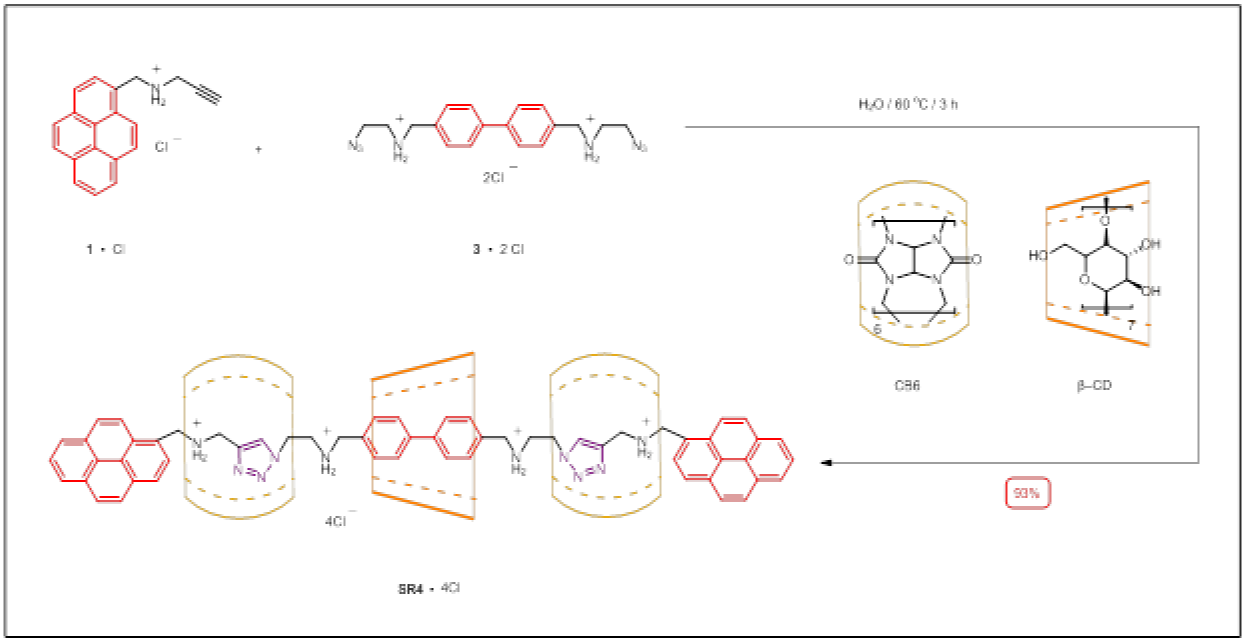

The stopper precursor **1**•Cl (110.2 mg, 0.36 mmol), the dumbbell precursor **3**•2Cl (50.7 mg, 0.12 mmol), and γ-CD (272.6 mg, 0.36 mmol) were mixed in H_2O (50 mL) and stirred at 60 °C for 10 min before CB6 (359.2 mg, 0.24 mmol) was added. The reaction mixture was stirred at 60°C for a further 3 h. Thereafter, the insoluble residues were filtered off from the reaction mixture. The filtrate was loaded directly onto a reverse-phase C18 column (150 gram, RediSep Rf Gold C18Aq) on an automatic column chromatographic system (Combiflash Rf200, Teledyne Isco) in order to isolate the desired product (eluent: H_2O / MeCN (0.1 % TFA) = 100 / 0 to 0 / 60 in 40 mins, flow rate: 85 mL/min). The appropriate fractions containing **SR4**$^{4+}$ were collected, and the solvent was removed under reduced pressure. The residue was dissolved in $MeNO_2$, and the

counterion of the **SR4**$^{4+}$ was exchanged to Cl^{-} on addition of an excess of tetrabutylammonium chloride. The resulting white precipitate was collected by vacuum filtration, washed with an excess of $MeNO_2$ and dried under vacuum to afford **SR4**•4Cl (464.3 mg, 93 %) as a white powder.

^{1}H NMR (500 MHz, D_2O, 298 K): δ = 8.89 (d, *J* = 9.3 Hz, 1H), 8.84 (d, *J* = 9.3 Hz, 1H), 8.53 (d, *J* = 7.9 Hz, 1H), 8.50 (d, *J* = 8.0 Hz, 1H), 8.35 (dt, *J* = 10.1, 4.7 Hz, 8H), 8.26 – 8.11 (m, 6H), 7.99 (d, *J* = 7.9 Hz, 2H), 7.90 (d, *J* = 8.0 Hz, 2H), 7.67 (d, *J* = 7.9 Hz, 2H), 7.53 (d, *J* = 7.9 Hz, 2H), 6.63 (s, 1H), 6.61 (s, 1H), 5.78 (d, *J* = 6.2 Hz, 12H), 5.75 (d, *J* = 6.8 Hz, 12H), 5.66 (d, *J* = 9.5 Hz, 12H), 5.63 (d, *J* = 9.8 Hz, 12H), 5.48 (s, 12H), 5.47 (s, 12H), 5.23 (d, *J* = 7.1 Hz, 4H), 5.02 (d, *J* = 3.1 Hz, 7H), 4.63 (s, 2H), 4.56 (s, 2H), 4.33 (t, *J* = 6.0 Hz, 2H), 4.27 (dd, *J* = 15.5, 4.3 Hz, 12H), 4.23 (s, 2H), 4.19 (dd, *J* = 15.7, 3.4 Hz, 12H), 4.00 – 3.85 (m, 2H), 3.81 (t, *J* = 6.7 Hz, 2H), 3.76 – 3.44 (m, 42H). ^{13}C NMR (125 MHz, D_2O, 298 K): δ = 156.5, 156.4, 156.2, 156.1, 140.5, 140.0, 139.2, 139.0, 132.5, 132.1, 130.9, 130.7, 130.5, 130.4, 130.3, 129.9, 128.37, 128.1, 128.0, 127.4, 126.6, 126.3, 125.9, 125.7, 124.9, 124.3, 124.0, 123.7, 123.6, 120.6, 119.8, 102.2, 81.1, 73.4, 71.8, 70.1, 62.5, 59.6, 51.5, 51.4, 51.2, 50.5, 50.1, 50.0, 46.4, 46.2, 45.8, 45.5, 43.2, 43.0. HR-ESI-MS: calcd for $[M - H - 4Cl]^{3+}$ *m/z* = 1340.1410, found *m/z* = 1340.1417; $[M - 4Cl]^{4+}$ *m/z* = 1005.3576, found *m/z* = 1005.3576.

Quantum Yield Measurements

Absolute fluorescence quantum yields were measured using a Horiba FluoroMax-4 spectrofluorometer with a Quanta-φ integrating sphere attachment. Data were analyzed by the 4-curve method using the FluorEssence 3.5 software. Typically, the quantum yield was calculated by

$$\varphi = \frac{E_s - E_b}{L_b - L_s}$$

where E_s is the integrated fluorescence profile of the sample caused by direct excitation, E_b is the integrated fluorescence profile of an empty integrating sphere (without the sample, only a blank), L_b is the integrated Rayleigh excitation peak of an empty integrating sphere (only a blank), and L_s is the integrated Rayleigh excitation peak of the sample when it is directly excited by the incident beam.

When acquiring the sample and blank fluorescence profiles, the number of accumulated scans was increased from 1 to 20 summed scans on account of the weak emission in comparison to the strong Rayleigh scattering. This factor of 20 was accounted for in the calculation of the quantum yield by dividing ($E_s - E_b$) by 20.

Ink Writing Tests

Four type of inks for pen writing were prepared using **R4**•4Cl (0.5 mM), Ad•Cl (100 mM), γ-CD (100 mM) and **R4**$^{4+}$⊂γ-CD_2 (**R4**•4Cl = 0.5 mM, γ-CD = 100 mM) solutions, respectively. Typically, 0.5 mL of the ink was loaded into a fountain pen for writing tests. A wide selection of paper-based printing media have been tested, including copy paper of various brands and models, matte presentation paper (HP), glossy presentation paper (HP), newsprint papers, rag paper (100% cotton, without optical brightener) and cigarette rolling paper. Banknote identification tests were performed on genuine or counterfeit banknotes of US Dollars, British Pounds Sterling, Euros, Chinese Yuan and Japanese Yen. In these tests, the corresponding currency symbols ($, £, €, and ¥) were drawn on the banknotes using the fountain pen filled with **R4**$^{4+}$⊂γ-CD_2 ink. The colour of the symbols on fake banknotes quickly changed from green to orange under the UV LED light, while the symbol on genuine banknotes retain their green colour. To simulate fake banknotes, images of the different banknotes were downloaded from the world wide web. One side of the fake banknotes was printed on different copy papers at 200% of its original size for use in the writing tests, after which the fake notes were destroyed.

The paper used in the writing test can generally be divided into two categories: 1) paper with optical brightener and 2) paper without optical brightener. Optical brighteners have been widely used in paper industry to make papers looks whiter under natural light. These optical brighteners are fluorescent materials which usually absorbs light in the UV region (340–370 nm) and emit blue fluorescence at around 420–470 nm. Optical brightener can interfere with the ink performance by reducing the contrast level. The widely used optical brightener includes stilbene derivatives, coumarins, imidazolines, etc. Many of those optical brighteners used in the paper industry can be encapsulated by γ-CD, resulting in a fluorescence colour change after writing.

Ink Printing Tests

Printing tests were performed on a HP inkjet printer (Photosmart CP4780 model, Supplementary Figure 71a) with customised ink cartridges. Rag papers (Canson Infinity Rag Photographique, 100% cotton, without optical brightener, no surface-coating side) were used for the printing tests based on the ink writing tests.

Ink cartridges for printing test were customised from the HP black and tri-colour cartridges (HP60 model). The filled inks were removed from the cartridge and washed extensively with H_2O and EtOH. The aqueous solutions of **R4**•4Cl (4 mL, 0.25 mM) and $\mathbf{R4}^{4+}\subset\gamma$-$CD_2$ (4 mL, **R4**•4Cl = 0.25 mM, γ-CD = 50 mM) were loaded into two cleaned black ink cartridges, respectively, to perform the monochrome printing tests. In the polychrome printing tests, the aqueous solutions of Ad•Cl (2 mL, 100 mM), $\mathbf{R4}^{4+}$/γ-CD (2 mL, **R4**•4Cl = 0.25 mM, γ-CD = 10 mM) and γ-CD (2 mL, 50 mM) were loaded into the magenta, yellow, and cyan channels of the cleaned tri-colour ink cartridge, respectively. Fluorescent colour under UV light was tuned by controlling the proportion of three inks in the customised tri-colour ink cartridge. In order to index the colours from a digital input file for printing with the desired fluorescent ink colours, two colour palettes were printed using the original tri-colour ink-cartridges and the customised ink cartridges. Translating the fluorescent ink colours to the correspondent visible colour in the standard colour palette in the digital input file accurately reproduces the desired fluorescent ink colours in the printed documents.

Supplementary References

1. T. Forster, Zwischenmolekulare energiewanderung und fluoreszenz. *Ann. Phys. (Berlin)* **2**, 55−75 (1948)

2. The neutral form of compound **S3** has been reported: A. Okamoto, K. Kanatani, I. Saito, Pyrene-labeled base-discriminating fluorescent DNA probes for homogeneous SNP typing. *J. Am. Chem. Soc.* **126**, 4820–4827 (2004).

3. M. Takahashi, Y. Suzuki, Y. Ichihashi, M. Yamashita, H. Kawai, 1,3,8,10-Tetrahydro-2,9-diazadibenzo[cd,lm]perylenes: synthesis of reduced perylene bisimide analogues. *Tetrahedron Lett*. **48**, 357–359 (2007).

4. S. Angelos, Y.-W. Yang, K. Patel, J. F. Stoddart, J. F. Zink, pH-Responsive supramolecular nanovalves based on cucurbit[6]uril pseudorotaxanes. *Angew. Chem. Int. Ed*. **47**, 2222–2226 (2008).

5. A. N. Basuray *et al.*, The chameleonic nature of diazaperopyrenium recognition processes. *Angew. Chem. Int. Ed.* **51**, 17436–17439 (2012).
